# Longitudinal connections and the organization of the temporal cortex in macaques, great apes, and humans

**DOI:** 10.1371/journal.pbio.3000810

**Published:** 2020-07-31

**Authors:** Lea Roumazeilles, Nicole Eichert, Katherine L. Bryant, Davide Folloni, Jerome Sallet, Suhas Vijayakumar, Sean Foxley, Benjamin C. Tendler, Saad Jbabdi, Colin Reveley, Lennart Verhagen, Lori B. Dershowitz, Martin Guthrie, Edmund Flach, Karla L. Miller, Rogier B. Mars

**Affiliations:** 1 Wellcome Centre for Integrative Neuroimaging, Department of Experimental Psychology, University of Oxford, Oxford, United Kingdom; 2 Wellcome Centre for Integrative Neuroimaging, Centre for Functional MRI of the Brain (FMRIB), Nuffield Department of Clinical Neurosciences, John Radcliffe Hospital, University of Oxford, Oxford, United Kingdom; 3 Donders Institute for Brain, Cognition and Behaviour, Radboud University Nijmegen, Nijmegen, the Netherlands; 4 Zoological Society of London, London, United Kingdom; University of Zürich, SWITZERLAND

## Abstract

The temporal association cortex is considered a primate specialization and is involved in complex behaviors, with some, such as language, particularly characteristic of humans. The emergence of these behaviors has been linked to major differences in temporal lobe white matter in humans compared with monkeys. It is unknown, however, how the organization of the temporal lobe differs across several anthropoid primates. Therefore, we systematically compared the organization of the major temporal lobe white matter tracts in the human, gorilla, and chimpanzee great apes and in the macaque monkey. We show that humans and great apes, in particular the chimpanzee, exhibit an expanded and more complex occipital–temporal white matter system; additionally, in humans, the invasion of dorsal tracts into the temporal lobe provides a further specialization. We demonstrate the reorganization of different tracts along the primate evolutionary tree, including distinctive connectivity of human temporal gray matter.

## Abstract 2

A comparison of the organization of connections in the temporal association cortex sheds light on specializations underlying the unique behavioral abilities of monkeys, chimpanzee, gorillas and humans.

## Introduction

Understanding the organization and function of the brain requires an appreciation of its phylogenetic context. In the study of primate brain evolution, the temporal lobe is of particular interest. Indeed, the temporal cortex as found in Primates is thought to be unique to this order and distinct from expansions of the lateral brain surface seen in Proboscidea, Cetacea, and Carnivora [1]. Within the Primate order, the external morphology of the temporal lobe differs substantially, ranging from nearly lissencephalic, possessing only the Sylvian fissure, to highly gyrified with multiple longitudinal sulci in great apes [1]. Functionally, the temporal association cortex is involved in some of the most high-level behaviors of primates, including categorization and conceptual processing [2,3], social cognition [4,5], and in the human semantic processing for language [6]. However, how the internal organization of the temporal lobe differs across species and how this relates to their different behavioral repertoires remains unknown.

To reliably investigate the organization of the temporal cortex in different species, one must first be able to identify precisely how one brain relates to the other. Most comparative studies used to focus only on generic measures, such as whole brain size, but brains can differ in many aspects of their organization [7–9]. Ideally, one would create maps of brain organization of a large range of species that can be formally compared with one another [10]. However, such maps are laborious to create and difficult to compare using traditional anatomical techniques. More recently, advances in neuroimaging techniques allow the investigation of gray matter organization and connectivity. Such data can be obtained in a reliable fashion and in a relatively short time [11,12]. Although neuroimaging does not always have the resolution of the gold-standard techniques, it does have the potential to allow direct comparison of brain organization across species on a macroscopic scale.

One way to compare temporal lobe architecture between species is by comparing the longitudinal white matter connections that can be reliably detected using neuroimaging, both in vivo and ex vivo [13]. Earlier work has identified a major difference between humans and other primates in the extension of the arcuate fascicle (AF), a major longitudinal tract running mostly dorsally to the Sylvian fissure between the frontal and posterior temporal cortex [14,15]. However, it has been suggested that other aspects of temporal cortex also differ across primates. One example concerns the relocation of areas due to differential cortical expansion between macaques and humans [16,17]. More recently, it has also been suggested that connectivity of temporal association cortex from tracts originating from primary visual and auditory cortices increased in humans and chimpanzees compared with macaques [18]. Recent data from both Klingler dissections and tractography indicate that the inferior longitudinal fascicle (ILF), which connects inferior temporal cortex with occipital regions, is composed of multiple subcomponents in humans [19]. This could suggest that this fiber system has undergone further specialization in humans in comparison with macaques, in which only one component has been described. Therefore, to understand temporal lobe organization, it is necessary to systematically compare the architecture of temporal tracts across a range of primate species.

To understand how the expansion of the temporal cortex in the ape and human lineages is reflected in changes in its macroscale connectivity, we here compared the longitudinal white matter tracts running along the temporal lobe and the dorsal AF between the human, chimpanzee and gorilla great apes, and macaque monkey brains using diffusion-weighted magnetic resonance imaging (MRI). We start by demonstrating that diffusion MRI tractography in macaques is able to reconstruct white matter tracts close to their definition by other methods such as tracers and dissection. Then, we use the same procedure to reconstruct the same tracts in the other species so that we can reliably compare their organization.

Importantly, any differences in the longitudinal tracts between species are likely to result in differences in the connectivity profile of parts of the temporal cortex gray matter. It has been suggested that each cortical area has a unique set of connections with the rest of the brain, the so-called connectivity fingerprint, and that this fingerprint is a crucial determinant of an area’s function [20,21]. Therefore, we also use the reconstructed white matter tracts to undertake a systematic comparison of the temporal lobe gray matter between the macaque and the human, with the aim to investigate how observed white matter differences between species affect their connectivity fingerprints.

## Results

### Longitudinal tracts in macaque and human

We used connectivity-based parcellations of coronal regions of interest (ROIs) through the temporal white matter to identify the bodies of the main longitudinal tracts of the temporal lobe, which subsequently informed the position of the masks used to perform probabilistic tractography in order to reconstruct the course of the tracts.

**Fig 1:**
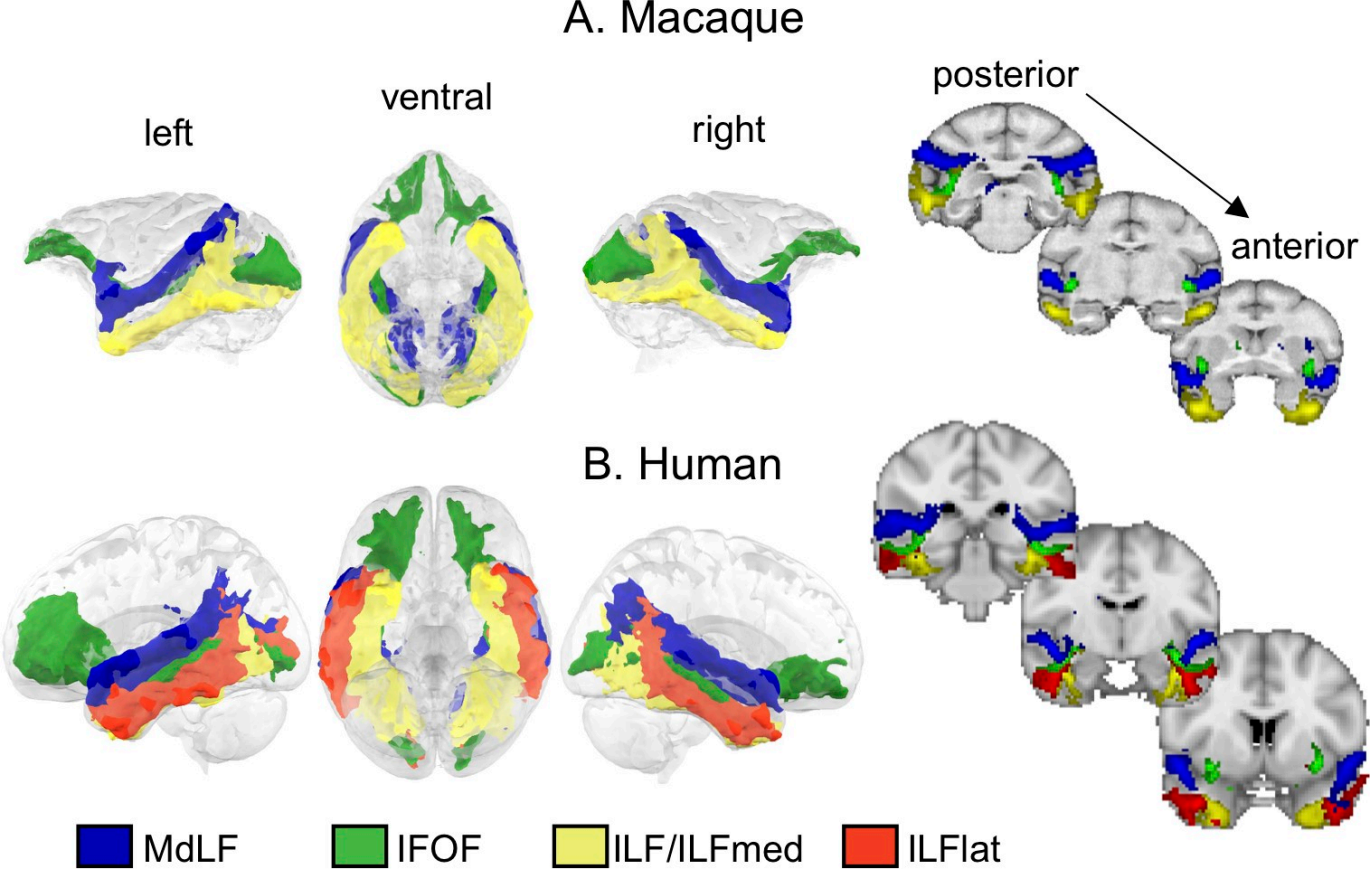
**A 3D representation and coronal sections of longitudinal temporal tracts in macaques (A) and humans (B).** Tractograms were log transformed and normalized for display. The coronal sections from left to right are taken from posterior to anterior. Blue, MdLF; green, IFOF; yellow, ILF (macaque) or ILFmed (human); red, ILFlat. Thresholds for the tracts are as follows: 0.7 for MDLF; 0.75 for IFOF; and 0.7 for ILF, ILFmed, and ILFlat. Human data are available from the Human Connectome Project (www.humanconnectome.org). Macaque postmortem data are available from the PRIME-DE repository (http://fcon_1000.projects.nitrc.org/indi/PRIME/oxford2.html) [24]. IFOF, inferior fronto-occipital fascicle; ILF, inferior longitudinal fascicle; ILFlat, inferior longitudinal fascicle lateral; ILFmed, inferior longitudinal fascicle medial; MdLF, middle longitudinal fascicle; PRIME-DE, Primate Data Exchange.

In macaques, we were able to reconstruct tracts similar to earlier studies [13,20,22] (Fig 1A, S1 Fig). The superior temporal gyrus contained a single cluster, from which we reconstructed a tract that can be identified as the middle longitudinal fascicle (MdLF) because of its course through the superior temporal gyrus and termination points mainly in the inferior parietal lobe but also branching toward occipital regions. The inferior temporal gyrus contained a single cluster, from which we reconstructed a tract similar to the ILF, terminating in inferior occipital and inferior parietal regions. Medial to these tracts, we identified a tract that is reminiscent of the inferior fronto-occipital fascicle (IFOF), running from the occipital cortex medially through the temporal cortex at the level of the inferior temporal gyrus. Among the tracts we identified, it was the only one to have very clear projections to the frontal lobe. The IFOF as identified here is likely to be a multisynaptic pathway because we are using the method of tractography and not tract tracing. Indeed, tract tracing identifies a monosynaptic section of the IFOF termed the extreme capsule [22]. Importantly, a recent dissection study identified an IFOF in the macaque similar to our tractography results, demonstrating a biological basis of our finding [23]. We provide a more in-depth comparison of these tracts as obtained using different methodologies in the Supporting information section (S1 Fig, S1 Text).

**Fig 2:**
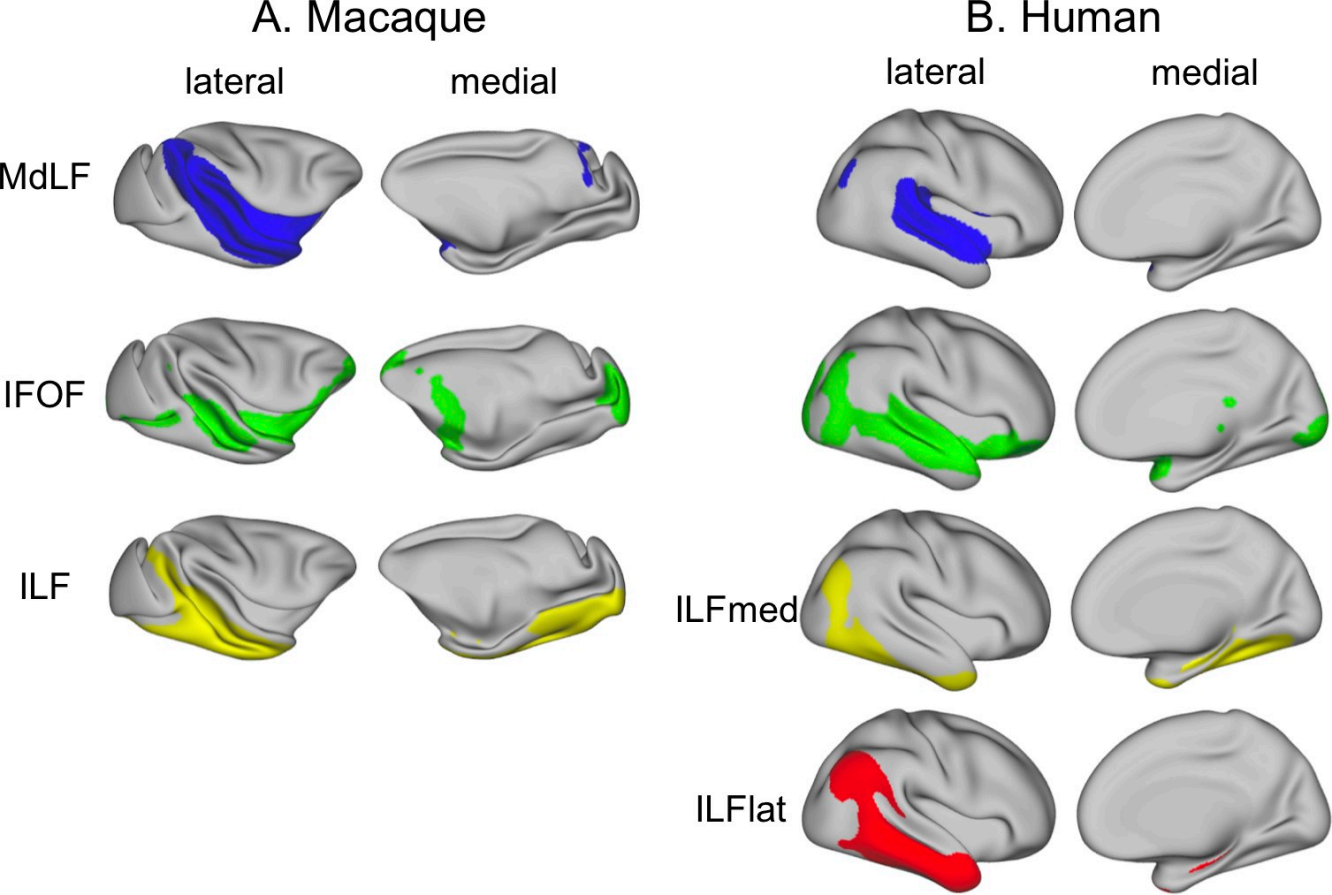
**Surface projection of longitudinal temporal tracts in macaques (A) and humans (B).** Shown are the group averages of the normalized, log-transformed, and thresholded tracts in the right hemisphere only (left hemisphere results can be found in S2 Fig). Blue, MdLF; green, IFOF; yellow, ILF (macaque) or ILFmed (humans); red, ILFlat. Thresholds for the tracts are as follows: 0.7 for MDLF; 0.75 for IFOF; and 0.7 for ILF, ILFmed, and ILFlat. Human data are available from the Human Connectome Project (www.humanconnectome.org). Macaque postmortem data are available from the PRIME-DE repository (http://fcon_1000.projects.nitrc.org/indi/PRIME/oxford2.html). IFOF, inferior fronto-occipital fascicle; ILF, inferior longitudinal fascicle; ILFlat, inferior longitudinal fascicle lateral; ILFmed, inferior longitudinal fascicle medial; MdLF, middle longitudinal fascicle; PRIME-DE, Primate Data Exchange.

The same procedure in humans identified a tract through the superior temporal gyrus similar to the MdLF (Fig 1B). In the inferior temporal gyrus, the clustering could reliably identify two clusters with differential connectivity to the rest of the brain where we would expect the territory of the ILF to be. We suggest that the higher number of clusters found in humans reflects a higher complexity in the temporal lobe white matter organization of this species. We interpret this in the light of previous studies supporting a subdivision of the ILF into two subcomponents [19,25]. Accordingly, the tractography identified different tractograms for the two subcomponents. One of them runs more laterally in the inferior temporal gyrus and slightly invades the middle temporal gyrus, whereas the other runs more medially and ventrally, mainly in the fusiform gyrus, and reaches more inferior occipital regions. We refer to these tracts as the ILF lateral (ILFlat) and ILF medial (ILFmed) subcomponents of the ILF. We identified a tract very similar to the IFOF that runs predominantly through the middle temporal gyrus, although it is located more medially than the other two tracts. Indeed, from the surface projections (Fig 2A and 2B, S2 Fig), it can be seen that the middle temporal gyrus in humans is mainly reached by the ILFlat and the IFOF.

In summary, compared with the macaque, the human IFOF is located more lateral and runs predominantly along the middle temporal gyrus (which is not present in the macaque). Also, unlike macaques, human ILF can be subdivided into two subcomponents. The MdLF has a similar path in both species, which indicates that its main connectivity pattern across species should not be fundamentally different.

### Longitudinal tracts in great apes

**Fig 3:**
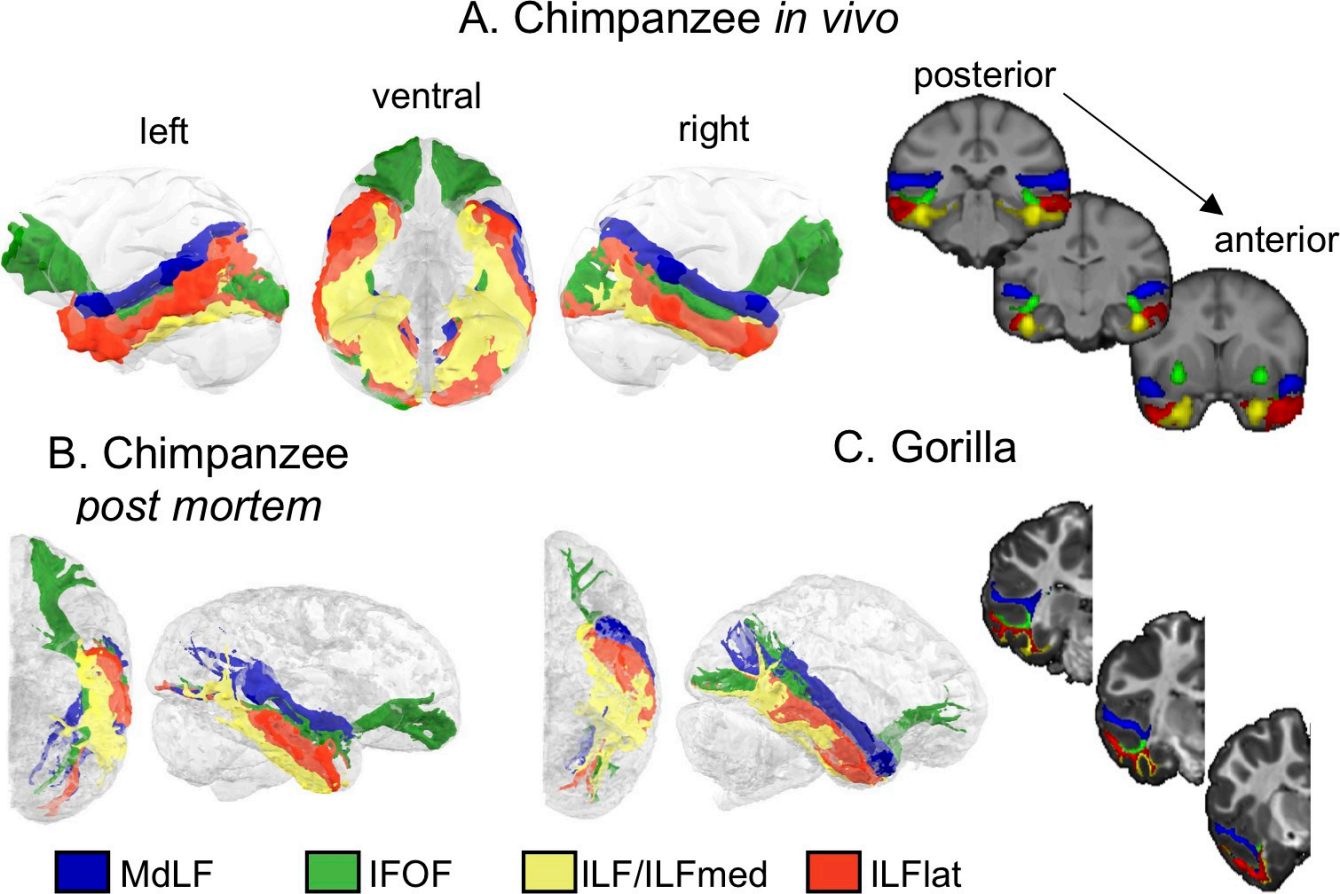
**A 3D representation of longitudinal temporal tracts in chimpanzees in vivo (A) postmortem (B) and gorilla (C).** Tractograms were log transformed and normalized for display. The coronal sections from left to right are taken from posterior to anterior. Blue, MdLF; green, IFOF; yellow, ILFmed; red, ILFlat. Thresholds for the tracts are as follows: 0.7 for MDLF; 0.75 for IFOF; and 0.7 for ILFmed and ILFlat. In vivo chimpanzee data are available from the National Chimpanzee Brain Resource (www.chimpanzeebrain.org). Gorilla and chimpanzee postmortem data are available from https://doi.org/10.5281/zenodo.3901205. IFOF, inferior fronto-occipital fascicle; ILF, inferior longitudinal fascicle; ILFlat, inferior longitudinal fascicle lateral; ILFmed, inferior longitudinal fascicle medial; MdLF, middle longitudinal fascicle.

Using the same procedure of connectivity-based clustering followed by tractography as for humans and macaques, we identified longitudinal temporal tracts in the chimpanzee and gorilla. We used both in vivo and ex vivo data in the chimpanzee and obtained comparable results (Fig 3A and 3B), indicating that our results generalize across the different imaging protocols. However, because the left hemisphere in the postmortem sample was slightly flattened in the extraction protocols, it is hard to interpret the results obtained in that hemisphere.

We identified the MdLF running through the superior temporal lobe following a similar course as human and macaque MdLF, reinforcing the argument for a conserved tract across all studied anthropoid primates (Fig 3A, 3B and 3C). In the inferior temporal lobe, the territory occupied by the ILF was similar to that in humans and could similarly be divided into two subcomponents. We therefore will refer to them as ILFlat and ILFmed as well. Although the differentiation is strong in the chimpanzee, specifically in the right hemisphere, the separation of the ILF into two subcomponents in gorilla, although present, is less pronounced. Different methods to assess the number of clusters in the temporal white matter did not reach a consistent conclusion in the gorilla (S2 Text, S8 Fig), suggesting that the gorilla temporal lobe white matter might not be as complex as in chimpanzees and humans. We suggest that this is related to the anatomy of its temporal lobe, with a less prominent fusiform gyrus and a less distinct difference between the inferior and middle temporal gyrus in the gorilla, which is associated with a shallower inferior temporal gyrus, compared with the chimpanzee. The IFOF was also present in great apes, and its course was similar to the human IFOF, running more medially than other tracts as well as in the middle temporal gyrus. In chimpanzee in vivo, it seems that the IFOF does not reach as lateral as in the other great ape data. In gorilla, it can be observed that the IFOF is most prominent in its posterior section, whereas it is quite limited in its frontal projections. However, having only one sample for the gorilla because of the presence of a frontal lesion on the left hemisphere, we cannot draw too-strong conclusions from this observation.

**Fig 4:**
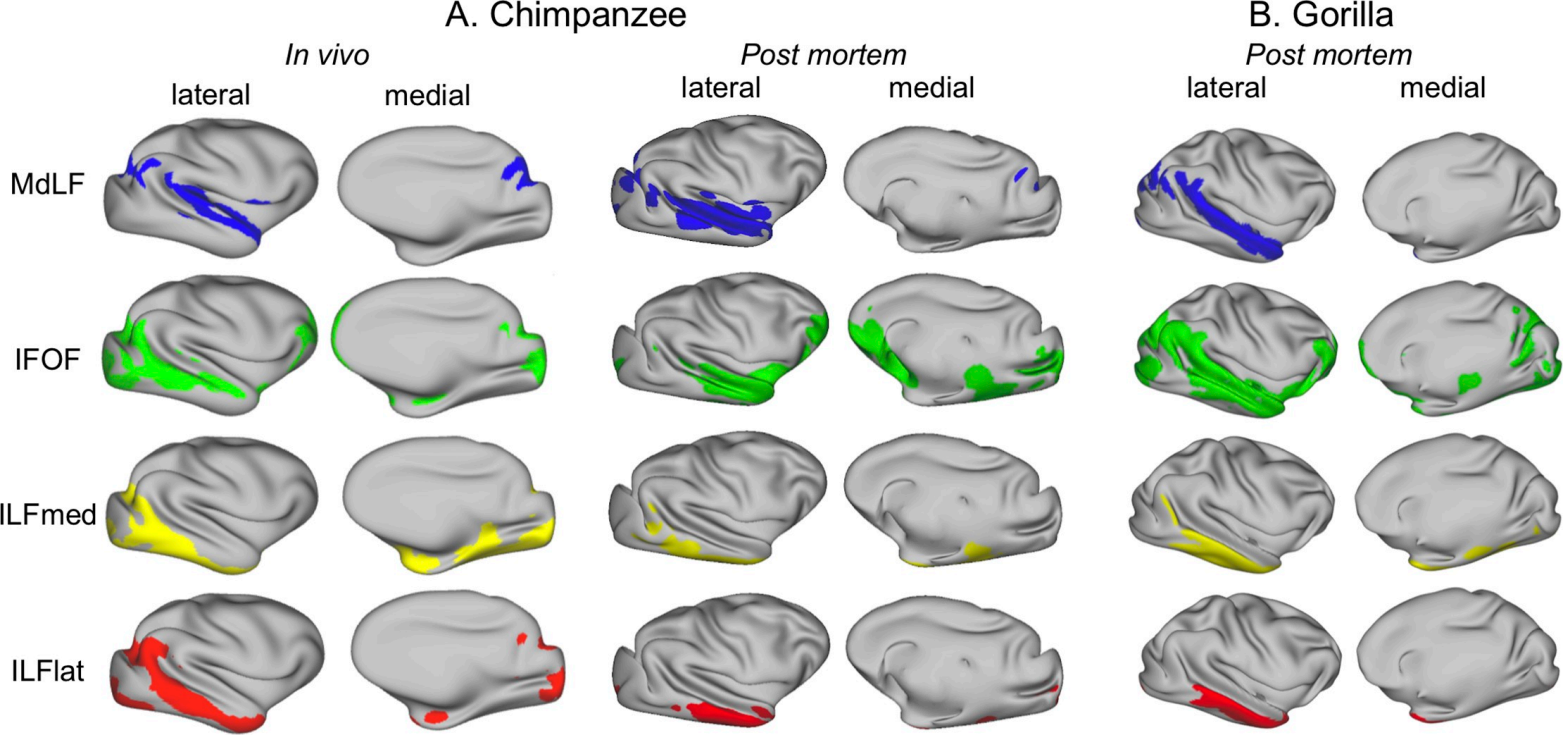
**Surface representation of longitudinal temporal tracts in chimpanzees (A) and gorilla (B).** Shown are the group average (chimpanzee in vivo) or individual results (chimpanzee postmortem and gorilla) of the normalized, log-transformed, smoothed, and thresholded right tracts (left hemisphere results can be found in S3 Fig for chimpanzee in vivo). Blue, MdLF; green, IFOF; yellow, ILFmed; red, ILFlat. Thresholds for the tracts are as follows: 0.7 for MDLF; 0.75 for IFOF; and 0.7 for ILFmed and ILFlat. In vivo chimpanzee data are available from the National Chimpanzee Brain Resource (www.chimpanzeebrain.org). Gorilla and chimpanzee postmortem data are available from https://doi.org/10.5281/zenodo.3901205. IFOF, inferior fronto-occipital fascicle; ILFlat, inferior longitudinal fascicle lateral; ILFmed, inferior longitudinal fascicle medial; MdLF, middle longitudinal fascicle.

To further illustrate these tracts in the great apes, we represented their tractograms on the cortical surface (Fig 4, S3 Fig). In other words, we examined where the tractogram approached the gray/white matter border. Although one should be cautious in interpreting such results because of the presence of superficial white matter systems [26] and possible gyral bias of tractography toward the surface, it provides an illustration of these fiber tracts in the great ape, in which they are currently underexplored. It allows one to represent not only the differences in tract organization with other species but also the sulcal and gyral patterns of these brains, which are not well known. This showed that for both species, the MdLF reaches mostly superior temporal gyrus areas as it does in the macaque and human. The ILF subcomponents are similar to those in the human, with the majority of the lateral part of the inferior temporal cortex reached predominantly by the ILFlat and the medial part reached predominantly by the ILFmed. The IFOF is the only one of these tracts that has projections to the frontal cortex.

Thus, great apes (including humans) seem to display a similar organization regarding the subcomponents of the ILF running in inferior and fusiform gyri, respectively. The ILFmed reaches an area reminiscent of the fusiform gyrus in all species, which is absent in the macaque, in which we did not detect the subdivision of the ILF. In line with this argument, we notice that these ILF subcomponents are less evident in the gorilla, in which the fusiform gyrus is less prominent than in chimpanzees and humans.

### Dorsal longitudinal tract

**Fig 5:**
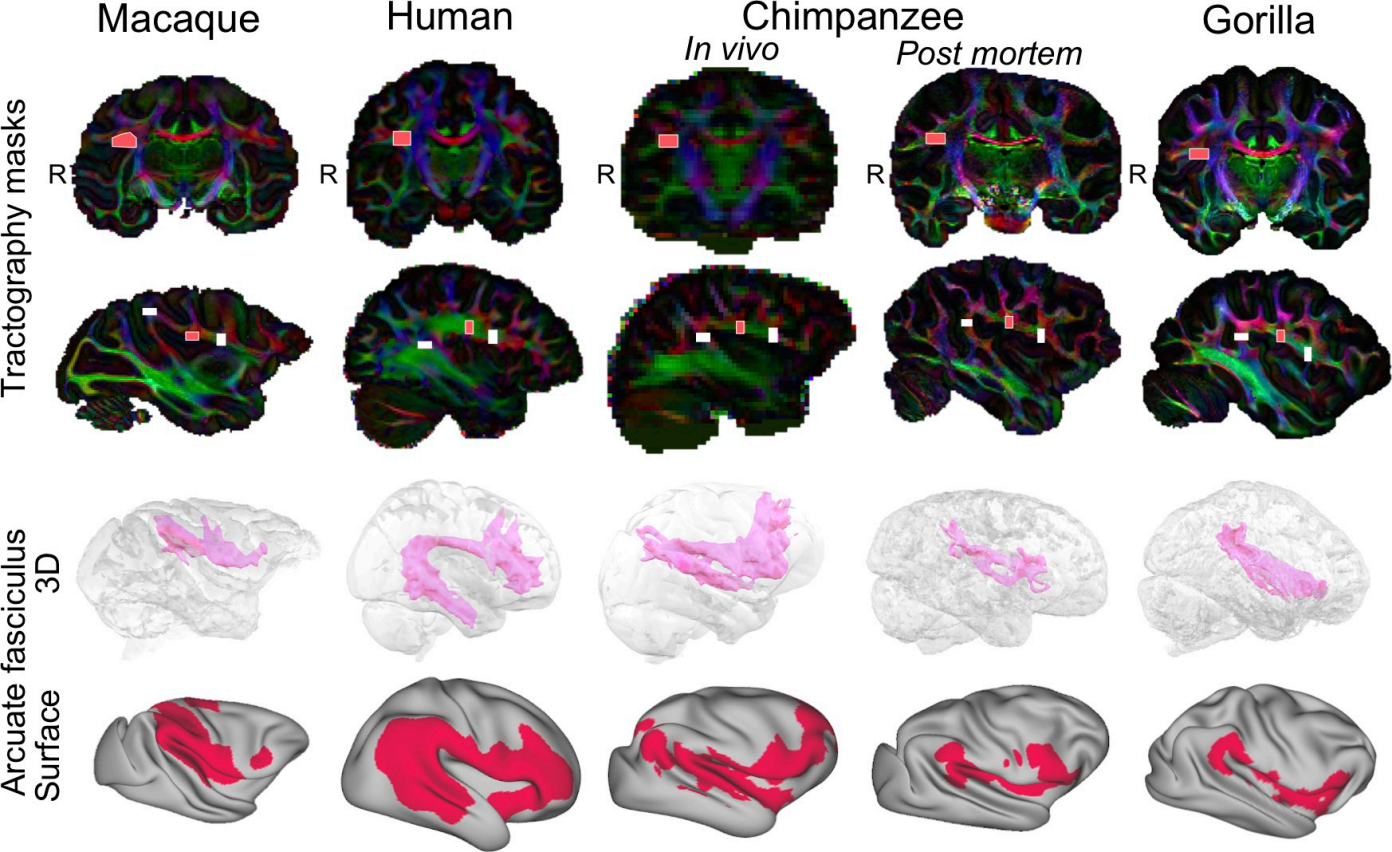
AF tractography protocols and results. Top panel: AF tractography masks example for one individual macaque, human, chimpanzee (in vivo and postmortem), and gorilla, represented on the principal eigenvector (V1) map weighted by the fractional anisotropy map. The light-pink mask represents the seed, and the white masks represent the anterior and posterior waypoints. Bottom panel: 3D and surface representation of the right tractogram obtained for AF. (Left hemisphere results can be found in S4 Fig) Threshold of 0.75. R denotes right hemisphere. Human data are available from the Human Connectome Project (www.humanconnectome.org). Macaque postmortem data are available from the PRIME-DE repository (http://fcon_1000.projects.nitrc.org/indi/PRIME/oxford2.html). In vivo chimpanzee data are available from the National Chimpanzee Brain Resource (www.chimpanzeebrain.org). Gorilla and chimpanzee postmortem data are available from https://doi.org/10.5281/zenodo.3901205. AF, arcuate fascicle; PRIME-DE, Primate Data Exchange.

We have concentrated thus far on the major ventral longitudinal tracts along the temporal cortex. However, a major dorsal longitudinal tract, the AF, runs between the frontal and temporal cortex. Previous studies have developed tractography protocols for this tract in the human, chimpanzee, and macaque, although the details differ between studies and groups [15,27]. Here, we used a consistent protocol to track the AF in all four species, creating a comparable result for the three species in which the AF has previously been defined and in the gorilla (Fig 5, S4 Fig).

In macaques, the AF consisted of only a dorsal component, whereas in humans, its course exhibited a sharp curve to end up in the temporal lobe running mainly in the middle temporal gyrus, although quite medially. In chimpanzees, we observed a small curve as well, with AF reaching the superior temporal gyrus but not the middle temporal gyrus, as in humans. An arcuate path similar to that of the chimpanzee could be observed in gorilla. Thus, the AF expansion to the temporal lobe seems to be unique to humans and not present in other great apes studied here.

### Divergence in macaque and human temporal cortex connectivity profiles driven by longitudinal tracts

The goal of the present work is to establish similarities and differences between the long-range connections of the temporal lobe across primate species, with the macaque and the human as the two most studied samples. If differences in these long-range connections exist, they should result in different connectivity profiles of temporal cortex gray matter between the two species. To establish whether this is the case, we compared the connectivity profiles of each gray matter vertex of the temporal lobe—in other words, the temporal lobe connectivity blueprint—across species. The logic of the approach is that for each vertex in each species, we quantify how well its connectivity fingerprint with the temporal cortex white matter tracts resembles that of any vertex in the other species. Thus, vertices with a low divergence score have a connectivity fingerprint also present in the other species, whereas vertices with a high divergence score are more likely to have a connectivity that is unique to that species. The connectivity fingerprint profile of a given vertex in this analysis is represented by the probability that the vertex is reached by each tract.

**Fig 6:**
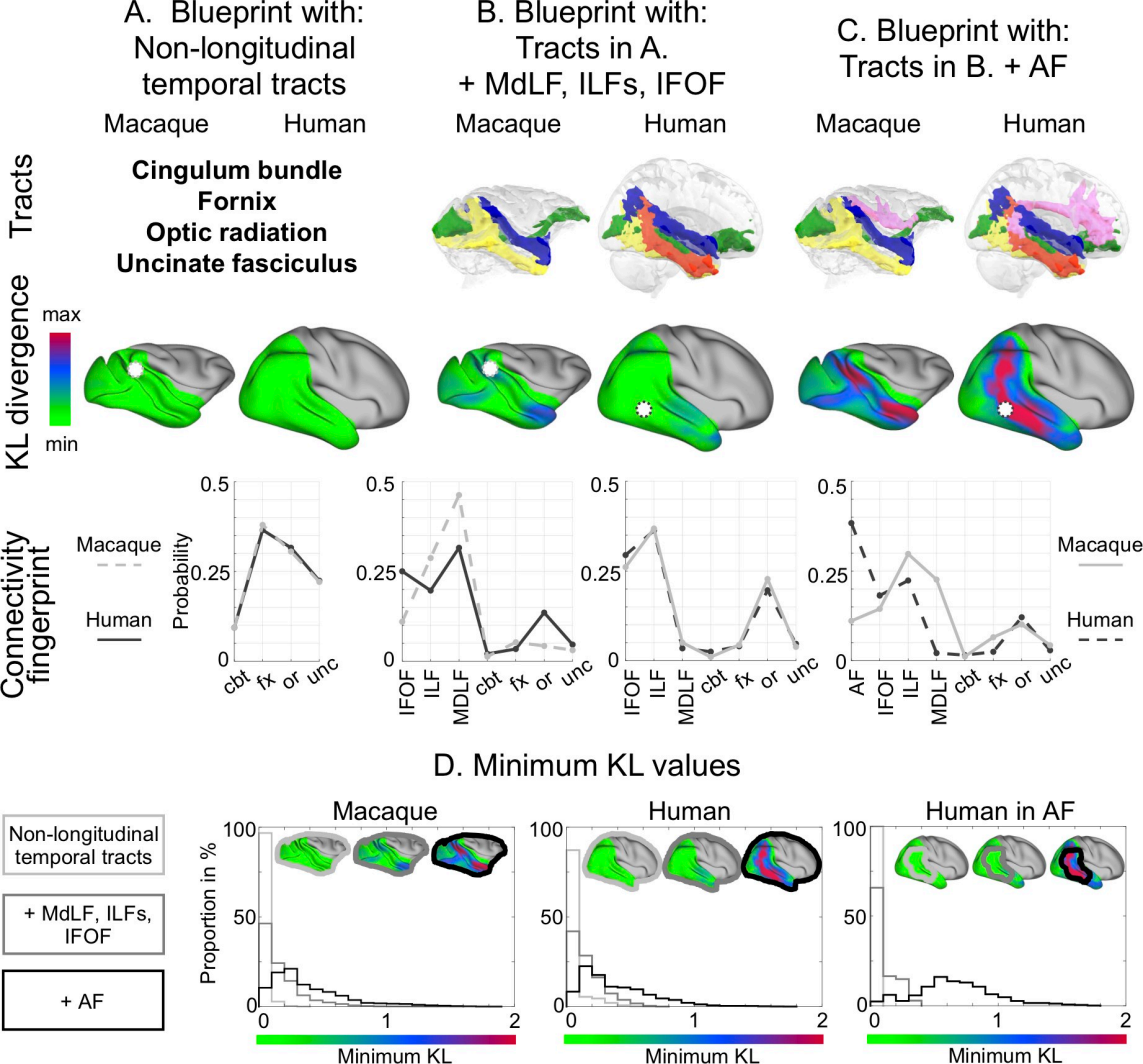
Results of the blueprint analysis. The figure reports results on the right hemisphere (left hemisphere results can be found in S6 Fig) and shows the tracts used (top row), the resulting KL divergence (middle row) for human predicting macaque (left) and macaque predicting human (right), and the connectivity fingerprints (bottom row). The greener the vertices of the KL divergence map in one brain, the more their connectivity profile is similar to that of vertices in the other brain. The connectivity fingerprints show how likely it is for the vertex, highlighted by a white sphere in the brain above, to be reached by each tract (dotted line). The solid line represents how likely each tract is to reach the average of the 10 best-matching vertices in the other species. (A) Blueprints established using the cbt, the fx, the or, and the unc. (B) Blueprints established with the tracts in (A) and adding the MdLF, ILF (both subcomponents combined in humans), and IFOF. (C) Blueprint established with the tracts in (B) and adding the AF. Blue, MdLF; green, IFOF; yellow, ILF (macaque) or ILFmed (human); red, ILFlat; pink, AF. (D) Distribution of minimum KL values obtained for each blueprint. From left to right for the macaque, the human, and masked with the human AF. Light gray, nonlongitudinal tracts; gray, adding MdLF, ILFs, and IFOF; black, adding AF. Human data are available from the Human Connectome Project (www.humanconnectome.org). Macaque postmortem data are available from the PRIME-DE repository (http://fcon_1000.projects.nitrc.org/indi/PRIME/oxford2.html). AF, arcuate fascicle; cbt, temporal part of the cingulum bundle; fx, fornix; IFOF, inferior fronto-occipital fascicle; ILF, inferior longitudinal fascicle; ILFlat, inferior longitudinal fascicle lateral; ILFmed, inferior longitudinal fascicle medial; KL, Kullback–Liebler; MdLF, middle longitudinal fascicle; or, optic radiation; unc, uncinate fascicle; PRIME-DE, Primate Data Exchange.

We first restricted the blueprint to four temporal tracts that have been well established in both humans and macaques—namely, the temporal part of the cingulum bundle, the fornix, the optic radiation, and the uncinate fascicle [27–30]. These tracts are known to be conserved across species, and hence we expect all vertices to have low divergence scores. Indeed, in this blueprint, we can observe that both human and macaque brain organization are predictable from one another (Fig 6A, S6A Fig). With those same tracts, but also adding the MdLF, the combined ILF, and the IFOF in the blueprint, we notice a small increase of the divergence score in some areas of the temporal lobe in both species (Fig 6B, S6B Fig). The example connectivity fingerprints in Fig 6A and 6B illustrate how the increase in divergence scores (computed as Kullback–Liebler [KL] divergence) is due to differences in the connections of these parts of the cortical surface with the underlying white matter. When only using the nonlongitudinal tracts (Fig 6A), all vertices have a low value because the pattern of connections of any part of the surface can be found in the other species. When adding the ventral longitudinal fibers, some differences are noticeable, and indeed a vertex with a high divergence score has a connectivity profile that differs even from its best matches in the other species (Fig 6B). Finally, when adding the AF, the profiles of connections of parts of the cortex are very different even from their best matches in the other species, indicating that this tract contributes most to the distinctive organization of the human temporal lobe (Fig 6C, S6C Fig).

These results are confirmed by the distribution of the divergence scores obtained with each blueprint (Fig 6D, S6D Fig). We can observe a shift toward higher minimum divergence scores in the distributions when adding the AF, which is confirmed by the distributions being statistically different (as assessed by the two-sample Kolmogorov–Smirnov test with *p* < 0.01). This shift is even more dramatic when focusing the human blueprint on the AF territory, confirming its influence on the increase of KL divergence.

**Fig 7:**
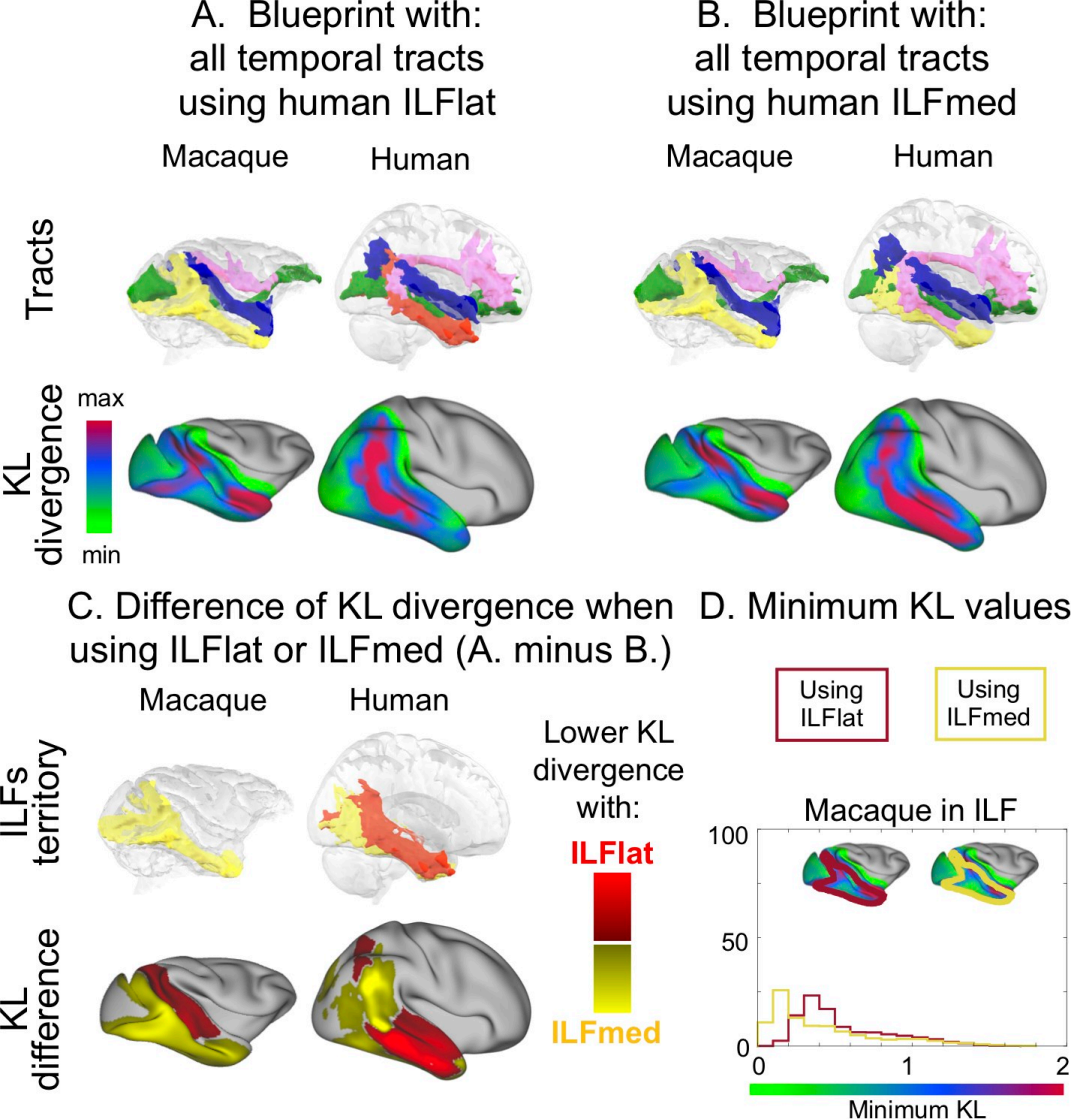
Blueprint method to determine differences between the human ILFs. **(**A) Blueprint established as in Fig 6C but with the ILFlat for humans (no ILFmed). (B) Blueprint established as in (A) but with the ILFmed for humans (no ILFlat). (C) The different ILF tractograms are represented on the top row. On the bottom row is shown the KL difference between the two maps established in (A) and (B). More yellow vertices means that using ILFmed in humans as macaques’ ILF homologous results in lower KL divergence between the two species at these vertices, whereas more red applies to ILFlat. Blue, MdLF; green, IFOF; yellow, ILF (macaque) or ILFmed (human); red, ILFlat; pink, AF. (D) Distribution of minimum KL values in the macaque’s ILF territory obtained for the blueprints with the different human ILF subcomponents. Red, with human ILFlat; yellow, with human ILFmed. Human data are available from the Human Connectome Project (www.humanconnectome.org). Macaque postmortem data are available from the PRIME-DE repository (http://fcon_1000.projects.nitrc.org/indi/PRIME/oxford2.html). IFOF, inferior fronto-occipital fascicle; ILF, inferior longitudinal fascicle; ILFlat, inferior longitudinal fascicle lateral; ILFmed, inferior longitudinal fascicle medial; KL, Kullback–Liebler; MdLF, middle longitudinal fascicle; PRIME-DE, Primate Data Exchange.

One difference we found between the macaques and all the great apes was the dissociability of the two components of the ILF. Using the blueprint method, we next investigated the different impact of the two subcomponents of the ILF on similarity of macaque and human temporal lobe organization. When we ran the same analysis comparing macaque temporal lobe with a human temporal lobe in which the ILF either consists solely of the ILFlat or consists solely of the ILFmed, we observed that macaque temporal lobe contains substantially more vertices with a high KL divergence in the territory of the ILF when we use ILFlat. In other words, macaque and human temporal lobes are more similar if we assume equivalence of macaque ILF and human ILFmed than if we assume equivalence of macaque ILF and human ILFlat (Fig 7A and 7B, S7A Fig, S7B Fig). Computing the difference between the two divergence maps analyzed with the two different subcomponents thus allows one to investigate the putative homology (Fig 7C, S7C Fig). On this difference map, we can also notice that when using the ILFmed, the human brain better predicts the areas where the macaque ILF runs, but the macaque brain does not predict the part where the ILFlat runs anymore. When using the ILFlat in humans, it better predicts an area situated more in the domain of the IFOF in macaques. The distribution of the divergence scores in the territory of the macaque ILF, obtained when using one or the other human ILF, also shows a shift toward higher value when using the human ILFlat compared with the human ILFmed (Fig 7D, S7D Fig). The two distributions are statistically different (as assessed by the two-sample Kolmogorov–Smirnov test with *p* < 0.01).

## Discussion

**Fig 8:**
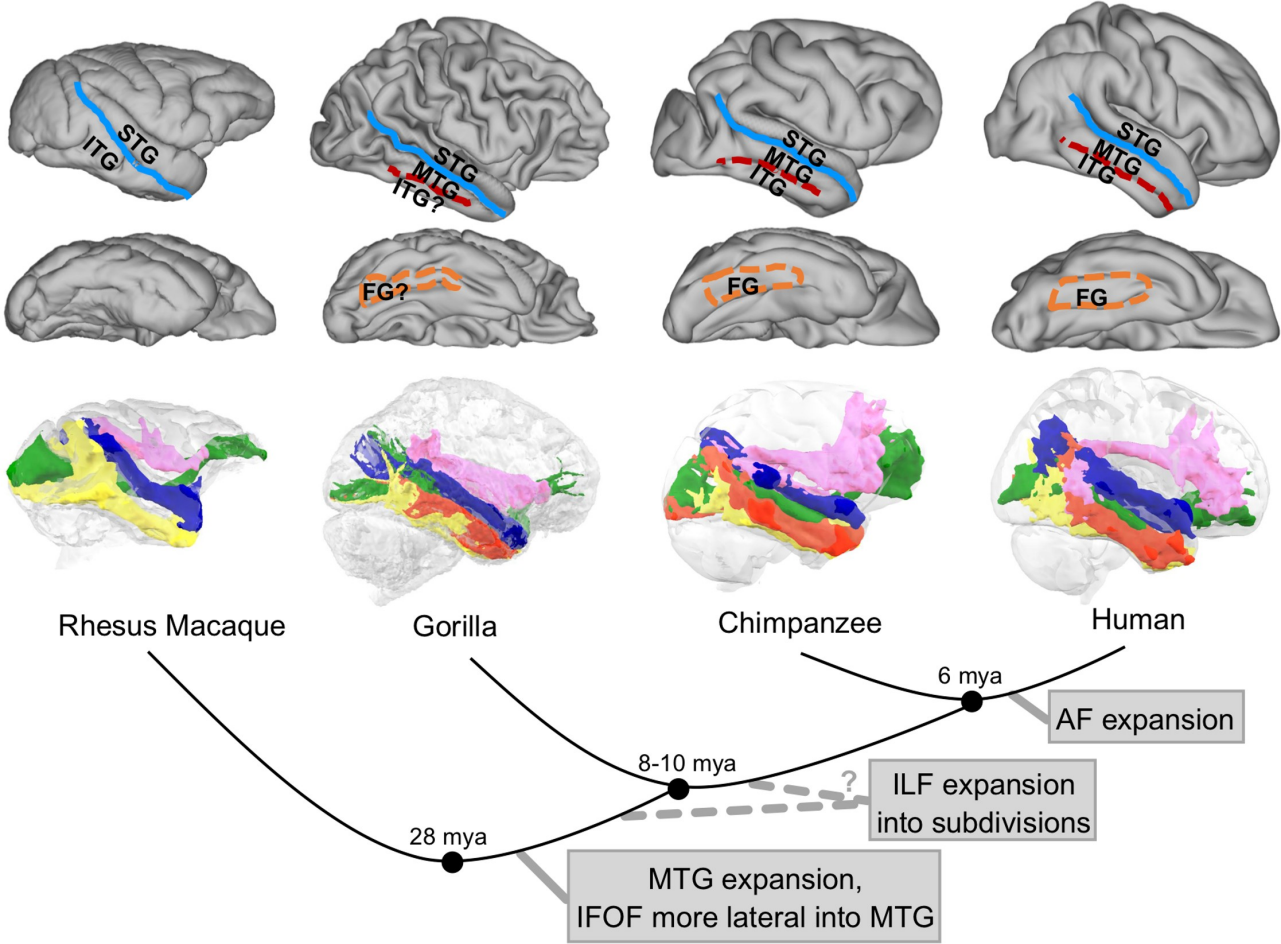
Suggested evolutionary trajectory for the longitudinal temporal lobe tracts. In the top panel are represented the major temporal anatomical landmarks in rhesus macaque, human, chimpanzee, and gorilla (brains not to scale). In the bottom panel, we represented the temporal tracts obtained in the different species. Blue, MdLF; green, IFOF; yellow, ILF (macaque) or ILFmed (human); red, ILFlat; pink, AF. Human data are available from the Human Connectome Project (www.humanconnectome.org). Macaque postmortem data are available from the PRIME-DE repository (http://fcon_1000.projects.nitrc.org/indi/PRIME/oxford2.html). In vivo chimpanzee data are available from the National Chimpanzee Brain Resource (www.chimpanzeebrain.org). Gorilla postmortem data are available from https://doi.org/10.5281/zenodo.3901205. AF, arcuate fascicle; FG, fusiform gyrus; IFOF, inferior fronto-occipital fascicle; ILF, inferior longitudinal fascicle; ILFlat, inferior longitudinal fascicle lateral; ILFmed, inferior longitudinal fascicle medial; ITG, inferior temporal gyrus; MdLF, middle longitudinal fascicle; MTG, middle temporal gyrus; PRIME-DE, Primate Data Exchange; STG, superior temporal gyrus.

Association cortex volume scales differently in monkeys and apes [31–33], which can be taken to indicate a grade change with respect to this aspect of brain organization because a grade designates a group of species based on similar morphological traits. Here, we investigate how these differences in volume are accompanied by differences in long-range connectivity. We show that the main longitudinal tracts connecting the temporal lobe are present in the most often studied monkey “model” (the macaque), in two separate great ape species (chimpanzee and gorilla), and in the human brain. Importantly, we identify modifications of these bundles across the different lineages (Fig 8). First, the ILF in chimpanzees and humans is reliably separated into subcomponents that were not observed in the macaque. This result is less strong in the gorilla. Second, the middle temporal gyrus, which is present in all species except the macaque, seems to be most heavily connected by the IFOF, which is located more laterally than in the macaque. Finally, the AF’s extension into the temporal cortex seems to be a uniquely human specialization, with the lack of expansion previously identified in the chimpanzee replicated here in the gorilla. Although consistent with previous reports of differences in the organization of macaque and human temporal cortex [15,20,34], our report crucially demonstrates that differences between the two species are not due to a single change at a single moment in time but rather to a series of changes in white matter architecture.

We studied examples of two nonhuman great apes, the gorilla and the chimpanzee. Although their temporal lobe organization seems generally similar, some differences were apparent. Because it seems that the sulcal anatomy of the gorilla and chimpanzee differs in how clearly defined their fusiform gyrus is, we could expect differences in their long-range connectivity as well, but because of the scarcity of the gorilla’s data consisting of one hemisphere, we should be careful in interpreting them. One observation concerns the gorilla’s ILF, which seems less clearly subdivided than in the chimpanzee. We can also notice that the IFOF reaches the frontal lobe to a lesser extent and is more biased toward the posterior projections, as well as not invading the lateral middle temporal gyrus as much, as seen in chimpanzee. Finally, we can also raise the point that the AF shows even less invasion of the temporal lobe than in chimpanzee, looking more similar to that of the macaque. Overall, this study would suggest that the limited expansion of the temporal lobe in the gorilla leads to a less complex pattern of white matter organization than that observed in the chimpanzee.

The IFOF carries fibers from occipital cortex through the length of the temporal cortex into the frontal cortex. This tract is frequently discussed in the human literature, but its existence in the macaque monkey has been controversial [35]. It should be noted, however, that most macaque studies are based on tracer injections, which identified monosynaptic pathway, whereas the human results are based on tractography, which identified multisynaptic pathway and therefore usually results in larger white matter bundle. Recent studies using tractography for both species suggested the presence of an IFOF-like bundle extending all the way posteriorly to occipital cortex in both species [13], but it has been argued that this reflects an artifact of the tractography method [36]. This issue now seems resolved by recent Klingler dissections in the macaque monkey that provide evidence for a posterior extension of IFOF even in the macaque [23] and by similar reports in another Old World monkey species [37].

However, differences in IFOF across species are notable, with human IFOF showing more extended prefrontal projection than macaque IFOF. We also noticed some differences between the two species regarding which frontal areas are mostly reached by the IFOF. The macaques’ IFOF projects more to medial frontal regions than the human IFOF, which reaches mainly lateral ones. Concerning the path taken by the IFOF in the temporal lobe, a recent exploratory study provided evidence of IFOF in the chimpanzee and suggested that most connections seeded from the middle temporal gyrus reach this fiber bundle [38]. The present results confirm this finding, showing that in all species that have a middle temporal gyrus, the IFOF is the main longitudinal tract reaching it and that it is placed more laterally than in the macaque. This is interesting, given that IFOF has been suggested to carry information between language-related areas in the human brain [39], especially those involved in semantic processing [40]. A previous study subdividing the human IFOF has also shown that it was the more lateral component of the IFOF that was actually connected to language-related areas as opposed to its medial components, which seem to carry more visual information [41]. The present results, showing similarity between human and great ape in possessing a lateral IFOF, suggest that an anatomical substrate enabling cognitive functions related to language existed in the common ancestor of humans and great apes but not in macaques. This demonstrates that cortical features supporting behavior commonly associated only or especially with humans cannot be assumed to be unique to the human brain.

Evolution of linguistic abilities has often been associated with another longitudinal tract, the AF. Our results confirmed previous studies that have shown differences between primates species [14,15]. In humans, chimpanzees, and gorilla, we observed that the arcuate is innervating the temporal gyrus, whereas it targets only dorsal regions in macaques. Nevertheless, the extent of fibers reaching the middle temporal gyrus is substantially more elaborate in humans than in any other primate species studied to date, accounting for most of the distinctive organization of the human temporal lobe. However, because we are focusing only on the right hemisphere for the gorilla, we should be careful in generalizing this result, particularly because of the left asymmetry of the arcuate observed in humans [14].

We have reported that the great ape ILF can be subdivided into two subcomponents, the medial and the lateral. The medial ILF of humans seems to be most similar to the macaque ILF as classically defined in tracer data [29]. We can try to interpret the relevance of these subcomponents for brain function, although of course, we do not claim to ascribe a function to any white matter bundle as one can for a gray matter area. A recent review of neuropsychological studies [42] sheds light on some possible contribution of the ILF to functional networks, with one of the best described being face recognition. Damage to the ILF has indeed been associated with impairment in face tasks in humans [43]. Although face-responsive areas have been identified in both humans and macaques [44], their locations in the temporal lobe differ between the two species. In humans, the most often described face area is the fusiform face area (FFA) [45]. This is located in the fusiform gyrus—a gyrus that monkeys do not possess but which is reached by the ILFmed in the present study. The main face areas in monkeys are scattered throughout the middle superior temporal sulcus and more ventral areas such as temporal area TE [46]. Therefore, the finding of ILFmed being the most similar to the macaque ILF is consistent with the observation that ILFmed reaches the fusiform gyrus as macaque ILF reaches homologs of the FFA [29,46]. This argument is also in line with the knowledge of the chimpanzee face-processing system. Indeed, it is located in a similar location to that in humans (in the fusiform gyrus) [47], and the current study shows that the chimpanzee ILF could also be subdivided with the ILFmed reaching the fusiform gyrus.

Neuropsychological work also associates the medial part of ILF to other networks, such as the visual memory, scene perception, and the emotion recognition and valuation networks [42], whereas lateral parts of ILF seem to be more associated with reading, object recognition, and semantic networks. Although we need to be careful with interpreting these linkages, we can observe that the reading and semantic networks, which are more associated with uniquely human cognition, are associated to the ILFlat; the one that we showed here was the most dissimilar to the macaques’ ILF.

We have used diffusion MRI data in combination with probabilistic tractography to reconstruct white matter tracts. It should be noted that there have been critiques on certain aspects of the tractography method in recent years. Mostly, these critiques pointed out that tractography can lead to the identification of false positives and that tracking toward the cortical surface is difficult because of the presence of superficial white matter systems near the gray/white matter border [26,48]. It is important to point out that the manner with which the tractography was applied here mitigates these concerns. Rather than tracking from a point on the cortical surface to another point at the cortical surface, which indeed can be problematic for tractography [49], we focus here predominantly on the body of the tracts, which we define in a data-driven manner using anatomical priors. Reconstructing the bodies of cortical fiber bundles is generally quite reliable [27]. Furthermore, in the one analysis in which we rely on the tracts’ connections to the gray matter—namely, the diversion score analyses at the end of the results section—we do not track toward the surface but use a procedure that combines tracking from the body of the tract and tracking from all of the gray matter toward the white matter, which is less susceptible to the problems outlined above [20].

In this study, we have used a greater range of species than is common in comparative diffusion MRI studies [13,36,50,51], which allowed us to build confidence in our conclusions. However, we acknowledge that studies using a wider range of species are still needed. We have studied two nonhuman great ape species, the chimpanzee and gorilla, but our study neglected the Asian great apes and lesser apes, as well as taking the macaque monkey as the sole representative of the Old World monkeys. The scarcity of data in this field means that other apes and especially other Old World monkey as well as other primate species remain to be studied, but the accelerating pace of comparative neuroimaging, of which this study is an example, means that these studies can now be considered imminent. Studies on gray matter architecture in a large number of primate species present promising examples [52].

This study does not address the evolutionary pressures that gave rise to the cortical specializations described here. It has been suggested that cortical specializations in the prefrontal cortex along the anthropoid lineage arose from increasingly complex foraging challenges that came with new ecological niches [53]. In this framework, humans have further reduced foraging errors by relying more than other surviving great apes on mental trial and error based on mental time travel. A similar argument for temporal cortex is advanced in another monograph [54], which suggests temporal lobe modifications might have occurred in great apes and humans when they adapted to their environment by changing their social behavior. Although it is quite difficult to relate these findings to our study, we are nevertheless able to illustrate here that any structural specialization is derived from a modification of existing machinery present in the ancestral state. Hence, to understand any one brain, understanding the organization of closely related species provides essential information. We must be cautious, however, when inferring evolutionary pressure from brains that come from distant species because they have all continued to evolve since their common ancestor.

When observing differences in white matter anatomy across species, such as those reported here, it is important to ask whether these are differences related to more general changes in brain structure, such as increased cortical folding due to a larger brain size, or whether they represent specific adaptations to a particular niche. These different types of changes are often difficult to disentangle [8,55]. It is known that expansion of temporoparietal cortex has occurred many times in the primate lineage [56], and this might underlie some of the so-called uniquely human organization of the temporal cortex, such as the ventral location of the middle temporal area [57]. However, such general mechanisms cannot describe the phenomenon of “mosaic evolution,” which demonstrates that expansion can be selective to certain brain structures in certain lineages [58]. Similarly, these general mechanisms cannot predict the results observed here, i.e., increased complexity of the ILF in great apes and extension of the AF only in humans. Indeed, the AF extension in humans has been generally interpreted as a unique adaptation rather than a result of general mechanisms in the language literature [59–61]. Of course, this does not mean that the developmental mechanisms that give rise to these adaptations need not be the same generic mechanisms at work in all primates, but it does imply that the specific tracts can be a unique adaptation [62].

In sum, we show a more elaborate picture of temporal cortex white matter organization across selected species of the order Primates than previously demonstrated. The human expansion of the AF has become one of the most established results of early comparative diffusion MRI studies, but here we show the beginning of a more complete picture, focusing in particular on occipital–temporal systems subserved by the IFOF and ILF. The changes in these tracts result in unique connectivity profiles of the temporal cortex gray matter in the humans and other great apes, which has important implications for differences in their behavioral abilities.

## Materials and methods

### Ethics statement

All in vivo data (human and chimpanzee) are from publicly available databases. The human data were obtained from the Human Connectome Project (HCP), and the approved consent procedure is fully described in the core HCP literature referenced here (www.humanconnectome.org). The chimpanzee data were obtained from chimpanzeebrain.org and collected at the Yerkes Primate Center under procedures that were carried out in accordance with protocols approved by YNPRC and the Emory University Institutional Animal Care and Use Committee (approval no. YER-2001206). All in vivo chimpanzee scans were done prior to 2012 and thus prior to the 2015 implementation of United States Fish and Wildlife Service and National Institutes of Health regulations governing research with chimpanzees. The ex vivo data were acquired from deceased animals that died of causes unrelated to this research project. As such, the research did not require a Home Office License as defined by the Animals (Scientific Procedures) Act 1986 of the United Kingdom.

### Data acquisition and preprocessing

The acquisition and preprocessing of the diffusion MRI, structural, and surface data are described in detail in the Supporting information (S2 Text). Key parameters and information on the diffusion data are summarized in Table 1.

**Table 1:** Overview of diffusion data.

	Number of samples	Type of sample	Protocol of acquisition	Resolution (mm^3^)	b-values (s/mm^2^) or q-values (cm^−1^)
**Macaque**	4	Postmortem	2D diffusion-weighted spin-echo multislice (single shell)	0.6 × 0.6 × 0.6	b = 4,000
**Gorilla**	1	Postmortem	3D DW-SSFP	0.6 × 0.6 × 0.6	q = 300
**Chimpanzee postmortem**	1	Postmortem	3D DW-SSFP	0.6 × 0.6 × 0.6	q = 300
**Chimpanzee** **in vivo**	3	In vivo	Single-shot spin-echo echo planar (single shell)	1.8 × 1.8 × 1.8	b = 1,000
**Human**	10	In vivo	Slice-accelerated gradient echo EPI (multishell)	1.25 × 1.25 × 1.25	b = 1,000, 2,000, 3,000

Abbreviations: DW-SSFP, diffusion-weighted steady-state free precession; EPI, echo planar imaging

### Reconstruction of ILF, MdLF, and IFOF

**Fig 9:**
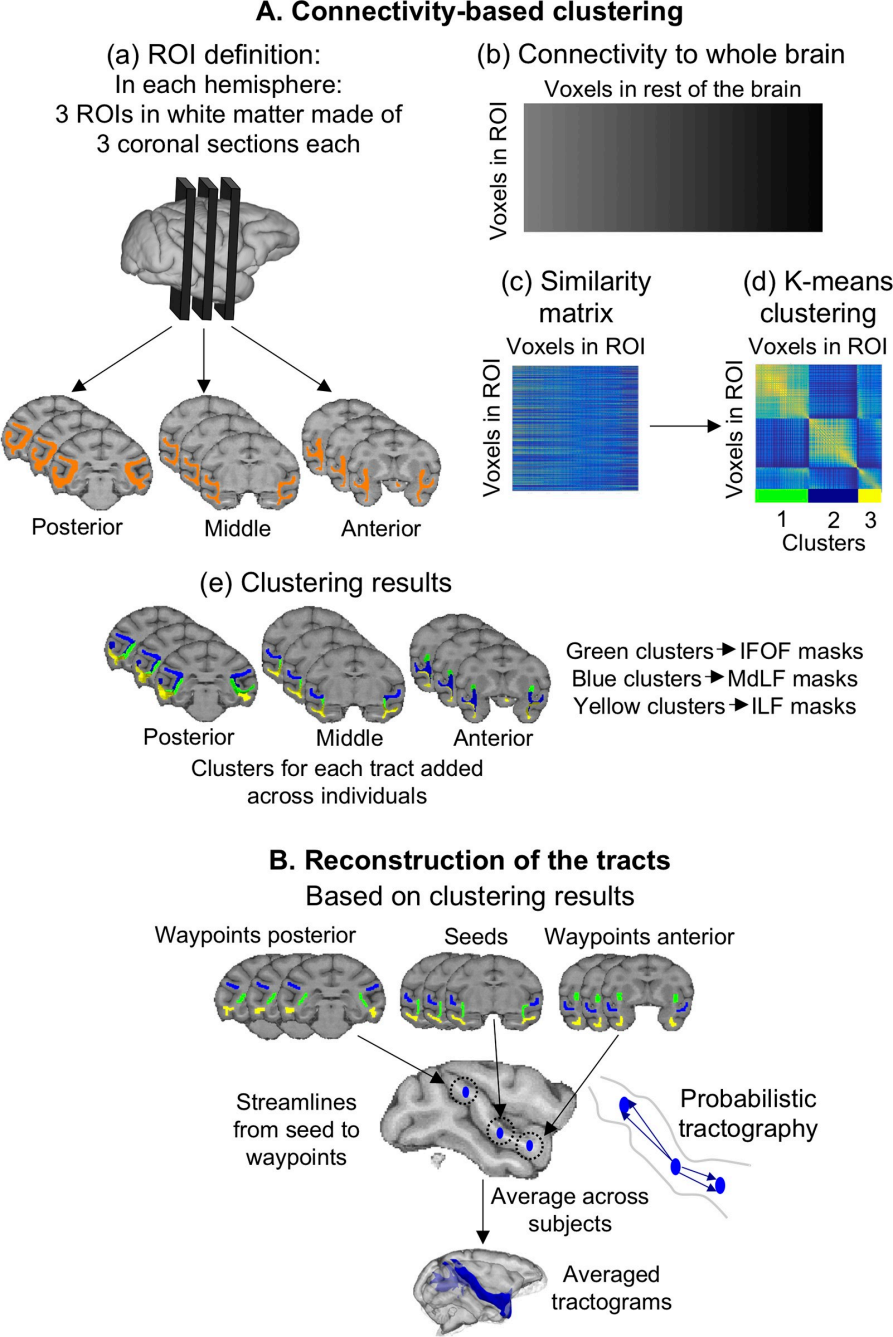
Methods overview. The macaque is shown as the example; the same methods were used for the other species, except that postmortem analyses were fully performed in subject space. (A) Connectivity-based clustering: (a) In each hemisphere, we defined three coronal ROIs in the white matter of the temporal lobe (excluding the area of the cingulum bundle). We transformed these ROIs to the diffusion space of each individual. (b) We then performed tractography from all the voxels in the ROI to the rest of the voxels in the whole brain. (c) We then computed the similarity matrix, representing the similarity in whole-brain connectivity of all voxels’ in each ROI. (d) We clustered the similarity matrix using the k-means algorithm, which resulted in clusters in the white matter ROI showing which voxels have similar connectivity to the rest of the brain. (e) These clusters were back projected onto each individual brain and transformed to standard space to give what we refer to as the “clustering results” (detailed in Figs 10 and 11). (B) Probabilistic tractography: from the clusters obtained at the clustering step, we established the masks for the tract reconstruction (detailed in Figs 10 and 11). Probabilistic tractography was performed as follows: streamlines were sent from the seed and were kept only if they went through one of the waypoints. The resulting tractograms were averaged across individuals. IFOF, inferior fronto-occipital fascicle; ILF, inferior longitudinal fascicle; MdLF, middle longitudinal fascicle; ROI, region of interest.

Our first aim was to reconstruct the three major longitudinal tracts running through the temporal cortex in all four species: the ILF, MdLF, and IFOF (sometimes referred to as extreme capsule fiber complex [13]). Given that these tracts have not been previously described in chimpanzee and gorilla, we developed an unbiased connectivity-based clustering approach to define tractography seed and waypoint masks in the temporal lobe white matter of all species. We established and validated the approach using human and macaque data because the tracts have been previously identified in these two species [19,22,63]. Briefly, our approach was divided into two steps. We first used a connectivity-based parcellation of three coronal ROIs in the temporal lobe white matter of each individual to identify the bodies of each of the longitudinal tracts based on their connectivity profile to the rest of the brain (Fig 9A). The clusters obtained, belonging to our tracts of interests, were subsequently used in the second step of tract reconstruction as seed and waypoint masks (Fig 9B).

The clustering step was first applied in the macaque and human to validate the approach and was performed as follows. For both species, we defined three ROI masks (anterior, middle, and posterior) in standard space (MNI152 1 mm for humans and F99 0.5 mm for macaques), each composed of three consecutive coronal sections and containing only the temporal lobe white matter (Fig 9Aa). The area corresponding to the body of the cingulum bundle was excluded. These ROI masks were transformed to each subject’s diffusion space prior to performing probabilistic tractography using FSL’s probtrackx from all the voxels in the ROI to all the voxels in the brain. This completely unconstrained tractography is part of the clustering step and is not intended as a method to define the whole tracts but to set up constraints for the actual tractography performed in the later tract reconstruction step. We thus obtained for each ROI a matrix of dimensions “number of voxels in ROI” × “number of voxels in the brain,” representing the connectivity of each voxel in the white matter ROI to the rest of the brain, and transformed it back to standard space (Fig 9Ab). From this matrix, we derived a similarity matrix of dimensions “number of voxels in ROI” × “number of voxels in ROI,” representing the similarity between each white matter ROI voxels regarding their connectivity to the rest of the brain (Fig 9Ac). The three similarity matrices (one for each ROI: anterior, middle, and posterior) were each submitted to k-means clustering to group together those voxels that have a similar profile of connections to the rest of the brain (Fig 9Ad) [64,65]. The logic of this approach is that all voxels belonging to the same fiber tract will show a similar connectivity profile, which enables us to infer each tract’s most likely location in the temporal white matter ROIs.

From the current knowledge of tract anatomy in the temporal lobe [19,22,27], we could then determine which cluster belonged to each tract for each subject. In macaques, we set three clusters in the middle and posterior ROI, which was presumed to reconstruct MdLF, ILF, and IFOF, except for the most anterior slice, in which we set it to four clusters to account for the presence of the uncinate fascicle. These assumptions are based on previous tract tracing literature [66]. In humans, we set one more cluster in each ROI because it has been shown that the ILF could be subdivided [19,25]. We justify a lower number of clusters in macaques by hypothesizing a more complex organization of temporal lobe white matter in humans than in macaque. This hypothesis has been verified by various measures that we detail in S2 Text and illustrate in S8 Fig.

**Fig 10:**
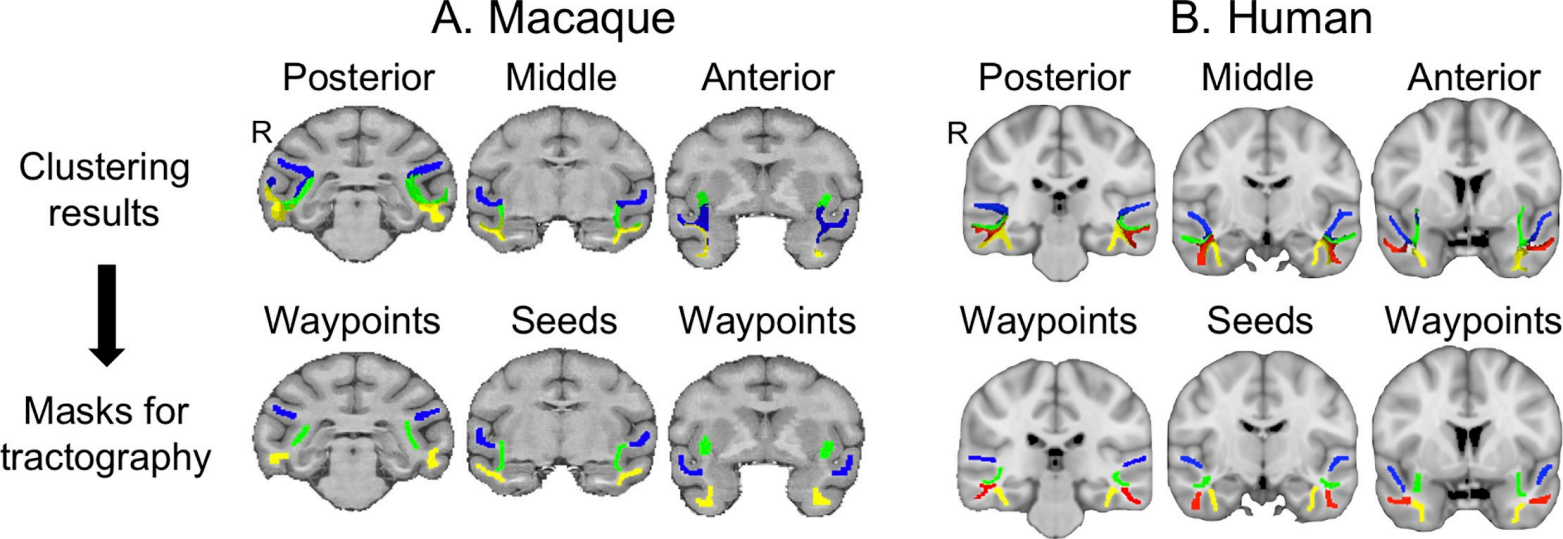
**Tractography masks (bottom row) derived from clustering results (top row) in macaques and humans.** (A) In macaques, the clustering results are thresholded to show the overlap between at least two subjects out of four. (B) In humans, the clustering results are thresholded to show the overlap between at least four subjects out of 10. Blue, MdLF; green, IFOF; yellow, ILF (macaque) or ILFmed (humans); red, ILFlat. R denotes right hemisphere. Human data are available from the Human Connectome Project (www.humanconnectome.org). Macaque postmortem data are available from the PRIME-DE repository (http://fcon_1000.projects.nitrc.org/indi/PRIME/oxford2.html). IFOF, inferior fronto-occipital fascicle; ILF, inferior longitudinal fascicle; ILFlat, inferior longitudinal fascicle lateral; ILFmed, inferior longitudinal fascicle medial; MdLF, middle longitudinal fascicle; PRIME-DE, Primate Data Exchange.

After identifying which cluster belonged to each tract for all individuals in one species, we then added the clusters belonging to the same tract in each coronal ROI to obtain a probability map of cluster position for each tract and ROI. Thus, we obtained probability maps for anterior, middle, and posterior positions of each tract. We refer to these probability maps as “clustering results” (Figs 9Ae and 10, top row). Based on these maps, we defined the seed (from middle position) and waypoint masks (from anterior and posterior positions) for the subsequent tract reconstruction (Fig 10, bottom row). All the masks for a given species were made of the same number of voxels across tracts, and masks from different tracts did not overlap.

Following the clustering step to identify the bodies of all tracts in three different positions along the anteroposterior axis, we reconstructed, in a second step, each tract by performing probabilistic tractography informed by the clustering step. For each tract, the tractography started from the middle seed mask defined in the previous step and was constrained by two waypoints that were anterior and posterior to the seed and also defined in the clustering step. Probabilistic tractography, as implemented in FSL’s probtrackx algorithm, started streamlines in the seed, and each streamline then followed local orientations sampled from the posterior distribution given by bedpostX [67]; only streamlines that passed through one or the other waypoint were retained. To further constrain the tractography, we also defined exclusion masks based on the anatomical description of the tracts in previous literature. Streamlines hitting these exclusion masks were deleted (Fig 9B). The exclusion masks were drawn as follows: for all tracts, we used sagittal midline sections to avoid tract crossing to the other hemisphere; a dorsal exclusion mask to avoid self–back projection from the longitudinal tracts; and exclusion masks for the fornix, cingulum bundle, and AF seed (defined as described below), as well as the medial section of the optic radiation and the temporal section of the uncinate fascicle. For each tract, waypoints and seeds of the other tracts were also used as exclusion masks. Subcomponents of ILF, however, were not used as exclusion masks for each other. To avoid tracts erroneously crossing gray matter, we also used a termination mask consisting of the entire gray matter defined in subject space (using FAST from FSL [68] for macaques and using the individual surface for humans). When reaching these termination masks, the streamlines were terminated but not deleted. Because the macaques’ gray matter masks were less precise, we added an exclusion mask in insular cortex and putamen. The resulting tractogram was normalized for each subject by dividing its path distribution by the total number of generated streamlines not rejected by waypoint or exclusion mask criteria. We then averaged the tract distribution over all subjects and down-sampled the average tractogram to 1-mm resolution (isotropic voxels) in F99 space for macaques and 2 mm in MNI152 space for humans. For visualization, 3D renderings of the log-transformed and thresholded average tractograms were generated using in-house Matlab code.

Having performed these analyses for the human and the macaque, we then applied the same procedure to the chimpanzee and gorilla data. The in vivo chimpanzee data were treated exactly as the human and macaque data. We performed a clustering procedure by setting the number of clusters to four because of the similar anatomy of the temporal lobe gyri and sulci between humans, gorillas, and chimpanzees. Indeed, although previous studies have shown differences in the gyral pattern between hemisphere, including notably an asymmetry of the Sylvian fissure and occipital bending between chimpanzees and humans [69], their overall sulci pattern remains more similar than compared with the macaque, which lacks defined middle temporal and fusiform gyri [1]. We performed a similar analysis as in humans and macaques to confirm that this was an optimal number of clusters (S2 Text, S8 Fig). For the chimpanzees, the measures used pointed toward the solution using a high number of clusters. However, for the gorilla, the different measures do not uniformly point toward solutions using a high or low number of clusters, and having only one hemisphere available for this species, we cannot strongly conclude on this issue (S2 Text, S8 Fig).

**Fig 11:**
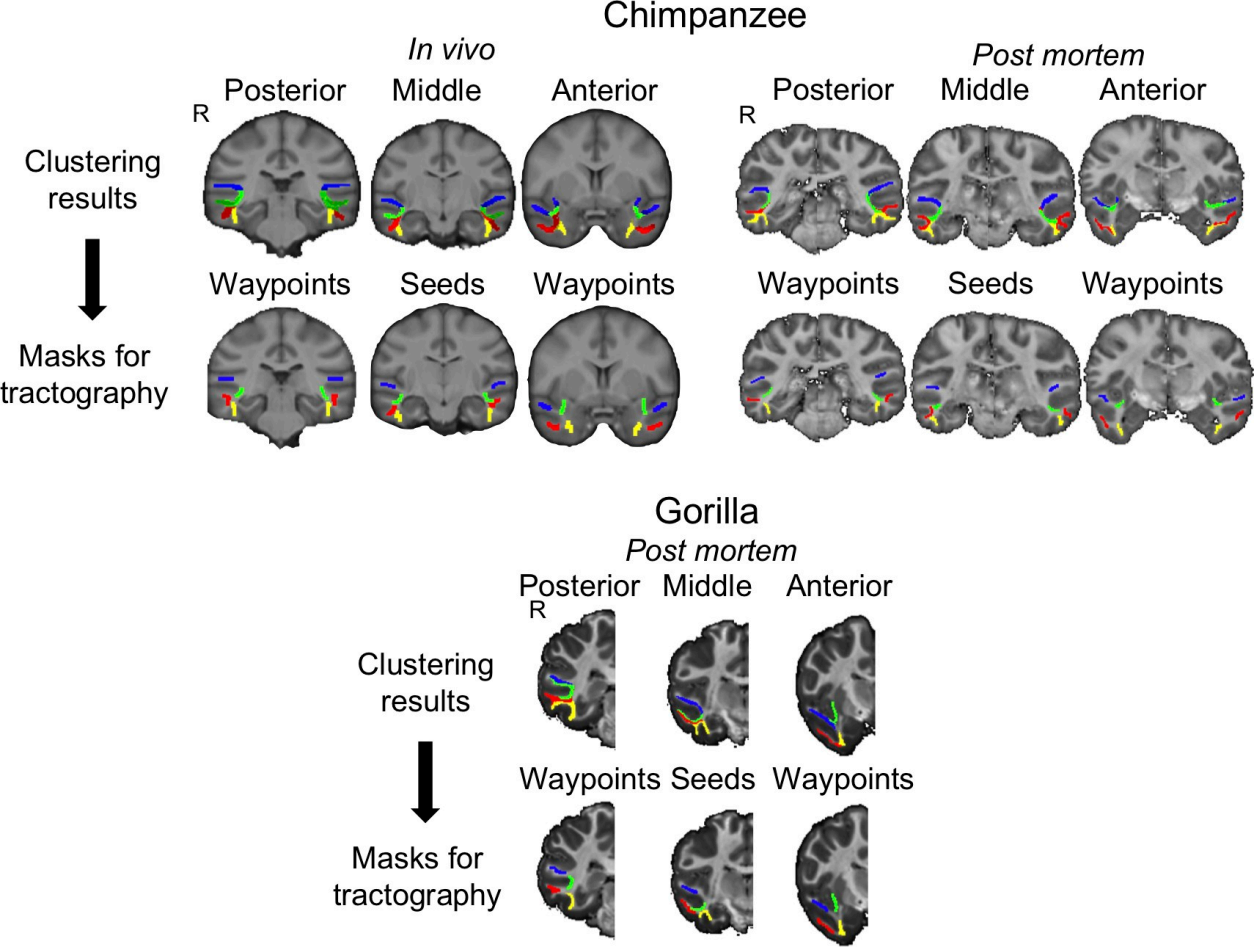
**Tractography masks (bottom row) derived from clustering results (top row) in great apes.** In chimpanzee in vivo, the clustering results show the three subjects overlapped (no thresholding is applied). One subject was available for each of the chimpanzee and gorilla postmortem samples. Blue, MdLF; green, IFOF; yellow, ILFmed; red, ILFlat. R denotes right hemisphere. In vivo chimpanzee data are available from the National Chimpanzee Brain Resource (www.chimpanzeebrain.org). Gorilla and chimpanzee postmortem data are available from https://doi.org/10.5281/zenodo.3901205. IFOF, inferior fronto-occipital fascicle; ILF, inferior longitudinal fascicle; ILFlat, inferior longitudinal fascicle lateral; ILFmed, inferior longitudinal fascicle medial; MdLF, middle longitudinal fascicle.

In this manner, we were able to define four tracts in the temporal lobe: the IFOF, MdLF, ILFlat, and ILFmed. From the clustering results (Fig 11, top rows), we defined the masks used in the tract reconstruction (Fig 11, bottom rows). The probabilistic tractography algorithm used the same exclusion masks as described for the macaques as well as the gray matter termination masks. The tract distributions obtained were averaged over three subjects and down-sampled to 1-mm resolution in Yerkes29 space. For the two postmortem ape samples (gorilla and chimpanzee), we used the same procedure as described above, except that all the analyses were only performed in the diffusion space of each subject and not transformed to a standard space because only one sample per species was available. Again, for visualization, 3D renderings of the log-transformed and thresholded average tractograms were generated using Matlab code made in-house. We also generated a surface representation of the tracts in great apes using tools from Connectome Workbench [70]. For the in vivo chimpanzees, we projected the averaged tracts to the Yerkes29 template (previously created from 29 chimpanzee scans) and we used the respective individual surfaces in T1 space for the postmortem gorilla and chimpanzee.

For the tract reconstruction, we used the same tractography settings in all species (10,000 samples, 2,000 steps, curvature threshold 0.2), except for the step length to account for differences in brain size and relative resolution (0.5 mm in humans and chimpanzees in vivo, 0.3 in gorilla and chimpanzee postmortem, and 0.25 in macaque postmortem).

### Reconstruction of AF

The AF is a dorsal longitudinal tract that runs from the inferior frontal cortex in a posterior direction and, in the human brain, curves ventrally to terminate in the temporal cortex. Because of its variability between species [15], we could not use the temporal cortex coronal ROI sections to identify it in all species with the clustering approach outlined above. We therefore adapted the method used previously by Eichert and colleagues [14] to reconstruct the tract from a manually defined seed outside the temporal cortex, just posterior to the level of the central sulcus (Fig 5 top rows). We also placed an axial mask as a posterior waypoint in the parietal–temporal white matter posterior to the caudal end of the Sylvian fissure. The mask was placed in a comparable location in all species to not bias the tract’s posterior course (Fig 5, top rows). To make the tractography comparable to that of the other longitudinal tracts outlined above, we added an anterior waypoint to the protocol of Eichert and colleagues [14], allowing us to track from a middle seed to anterior and posterior waypoints as well. We used the same exclusion masks for all species as in Eichert and colleagues [14]: a midline mask, two subcortical white matter and extreme/external capsule masks, and one mask through the superior parietal cortex.

In the great apes, we examined where the tractogram approached the gray/white matter border to represent their tractograms on the cortical surface (Fig 4, S3 Fig). We normalized, log transformed, smoothed with a kernel of 2 mm full-width half maximum (FWHM), and thresholded these surface tractograms.

The clustering and tract reconstruction steps have also been performed with alternative tractography techniques to validate our approach (S2 Text, S10–S14 Figs).

### Comparison of macaque and human temporal lobe connectivity blueprints

**Fig 12:**
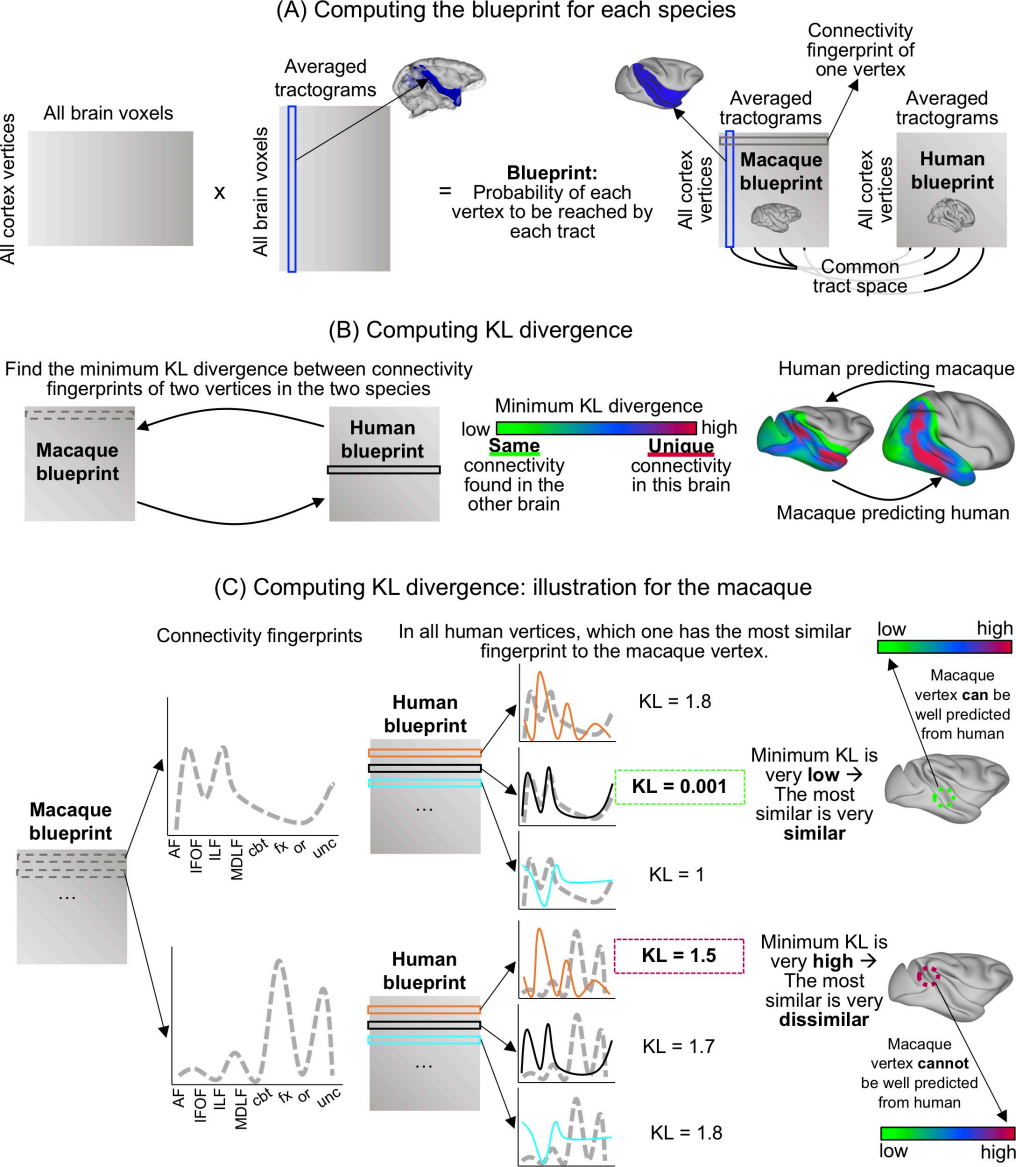
Blueprint method to assess connectivity divergence between macaque and human temporal cortex. (A) To compute the blueprint, we multiplied a matrix representing the connectivity of each cortical vertex (surface space) to each brain voxel (volume space) with a matrix representing how each brain voxel (volume space) is reached by each tract. Because we reconstructed the same tracts in both macaques and humans, the columns of the blueprint define a common space to compare the connectivity of the temporal cortex. The rows of the blueprint correspond to the connectivity fingerprints of each vertex. The columns of the matrix correspond to the surface tractograms. (B) Using the KL divergence measure to compare every vertex in one brain to every vertex in the other brain, we can identify vertices in the two brains in which the smallest divergence is found. If this smallest divergence is very low (green on the scale), it means that the same connectivity profile can be found at these vertices for both brains. If the smallest divergence is very high (pink on the scale), it means that the connectivity profile at this vertex is very different to any connectivity profile of the other brain vertices. (C) Illustration of the KL divergence computation for two macaque vertices. For each of the vertices, a connectivity fingerprint representing how the vertex is connected to each tract was computed. Using a KL divergence measure, this connectivity fingerprint was compared with connectivity fingerprints of all human vertices. The vertex with the connectivity fingerprint most similar to the macaque one, therefore with the smallest KL divergence, is picked, and this KL divergence value is assigned to the monkey vertex. AF, arcuate fascicle; cbt, temporal part of the cingulum bundle; fx, fornix; IFOF, inferior fronto-occipital fascicle; ILF, inferior longitudinal fascicle; KL, Kullback–Liebler; MdLF, middle longitudinal fascicle; or, optic radiation; unc, uncinate fascicle.

To establish the effects of any differences in the longitudinal temporal tracts across species on the connectivity profiles of temporal cortex gray matter, we established the white matter connectivity of each part of the temporal cortex gray matter, the so-called “connectivity blueprint,” using the method established by Mars and colleagues (2018) [20]. The connectivity blueprint is in effect a matrix that describes how well each vertex of the temporal lobe gray/white matter border, as defined in the surface, is reached by each of the white matter tracts of the temporal lobe. Each row of this matrix describes the “connectivity fingerprint” of each vertex of the temporal lobe with each white matter tract (Fig 12A). Therefore, the connectivity fingerprint is a subset of the connectivity blueprint focusing on a particular vertex instead of the whole brain. Each column of the blueprint matrix corresponds to one tract. Because the tracts are defined in a similar way in the different species, they present a common space that allows us to compare connectivity fingerprints across the two brains, testing whether there are unique connectivity fingerprints in one brain compared with the other.

We directly compared these blueprints between the macaque and the human brain (Fig 12). We focused on these two species because they are the most studied species among the ones presented in the current study. Furthermore, the course and connectivity of other nonlongitudinal tracts reaching the temporal lobe, such as the uncinate fascicle, the cingulum bundle, and the fornix, have been well established in these species [20,22].

We established the connectivity blueprint using the tractograms of ILF, MdLF, IFOF, and AF as established above and supplemented them with tractograms of the other major white matter tracts in the temporal lobe—namely, the temporal part of the cingulum bundle, the fornix, the optic radiation, and uncinate fascicle—using established tractography protocols [20]. A tractogram can be represented as a column vector describing how well each voxel is reached by the tract (Fig 12A). By concatenating the vectors corresponding to each tract, we obtained a matrix representing how each voxel (row) is reached by each tract (column). Second, we also calculated whole-hemisphere vertexwise connectivity matrices obtained by tracking from all vertices in the gray/white matter border of the surface file to all voxels in the brain in volumetric space (Fig 12A). These matrices were computed for both hemispheres and for each subject individually before creating an average for each hemisphere and species. Then, we multiplied the tractogram’s matrix with this averaged matrix in both species, obtaining the blueprint matrix (Fig 12A). We also individually extracted each column of this matrix to obtain a surface representation of the different tracts, which we will refer to as “surface projections.”

The connectivity blueprints of different species can be compared by calculating the KL divergence between the connectivity profiles of each vertex in one brain with that of each vertex in the other brain (Fig 12B). The KL divergence measures how the probability distribution of likelihoods of one vertex to be reached by each of the temporal lobe tracts is different in one brain from the probability distribution of each vertex in the other brain. For each vertex in one brain, we could then determine another vertex in the other brain that had the minimum KL divergence—in other words, the most similar connectivity profile. If a vertex in one species has a very similar connectivity profile to that of a vertex in the other brain, meaning that they are reached by a similar combination of tracts, the minimum KL divergence will be low. If, on the other hand, a vertex’s fingerprint connectivity profile is unique in one brain, its minimum KL divergence will be high (Fig 12C).

We first defined the blueprints based on the well-established tracts: the cingulum bundle, the fornix, the optical radiation, and the uncinate fascicle. Because we did not expect these tracts to be massively different between the two species, we expected the KL to be very low and, therefore, this comparison to serve as a reference point for the following blueprint comparisons. Then, we added the tracts of interest: MdLF, ILF, IFOF, and AF. To assess the reorganization of the human ILF subcomponents, we generated blueprints with both ILF subcomponents combined or either one or the other while keeping the macaque ILF constant. This manipulation enabled us to compare the ILF subcomponents’ distinct prediction of the macaque brain organization. We compared these maps visually by subtracting the KL divergence scores spatial map established with human ILFmed from the one established with human ILFlat. Furthermore, we masked the blueprint to only focus on the temporal, occipital, and inferior parietal lobes. To assess the divergence between species more quantitively, we obtained the distribution of the minimum KL divergence scores obtained with each blueprint: with nonlongitudinal tracts; adding MdLF, ILF, and IFOF; adding the AF; with the human ILFlat; and finally, with the human ILFmed (Fig 6D). We included only nonzero values, which are the vertices with differences in their connectivity fingerprints. Each distribution of minimum KL divergence scores indicates how similar the connectivity fingerprints of this brain are with that of another brain. All distributions were then compared using the two-sample Kolmogorov–Smirnov test (with *p* < 0.01).

For illustration, but not for establishing the blueprint, we smoothed the surface tracts with a kernel of 2 mm FWHM for macaques and 4 mm for humans. We then log transformed and normalized the surface tract distributions to facilitate the threshold determination.

## Supporting information

S1 Fig Comparison of the macaque tractography results with the tract tracing and dissection literature. (A) Comparison with tract tracing results redrawn from Schmahmann and Pandya (2006) [29]. Tracts to be compared are highlighted in similar colors. Blue, MdLF; green, IFOF; yellow, ILF; pink, AF. (B) Comparison with the dissection results redrawn from Decramer and colleagues (2018) [23]; the green crosses illustrate the IFOF pathway. Macaque postmortem data are available from the PRIME-DE repository (http://fcon_1000.projects.nitrc.org/indi/PRIME/oxford2.html). AF, arcuate fascicle; IFOF, inferior fronto-occipital fascicle; ILF, inferior longitudinal fascicle; MdLF, middle longitudinal fascicle; PRIME-DE, Primate Data Exchange.
(TIF)

S2 Fig **Surface projection of longitudinal temporal tracts in macaques (A) and humans (B).** Shown are the group averages of the normalized, log-transformed, and thresholded tracts in the left hemisphere only (for space-saving purpose). Blue, MdLF; green, IFOF; yellow, ILF (macaque) or ILFmed (humans); red, ILFlat. Thresholds for the tracts are as follows: 0.7 for MDLF; 0.75 for IFOF; and 0.7 for ILF, ILFmed, and ILFlat. Human data are available from the Human Connectome Project (www.humanconnectome.org). Macaque postmortem data are available from the PRIME-DE repository (http://fcon_1000.projects.nitrc.org/indi/PRIME/oxford2.html). IFOF, inferior fronto-occipital fascicle; ILF, inferior longitudinal fascicle; ILFlat, inferior longitudinal fascicle lateral; ILFmed, inferior longitudinal fascicle medial; MdLF, middle longitudinal fascicle; PRIME-DE, Primate Data Exchange.
(TIF)

S3 Fig Surface representation of longitudinal temporal tracts in chimpanzees. Shown are the group averages of the normalized, log-transformed, smoothed and thresholded left tracts. Blue, MdLF; green, IFOF; yellow, ILFmed; red, ILFlat. Thresholds for the tracts are as follows: 0.7 for MDLF; 0.75 for IFOF; and 0.7 for ILFmed and ILFlat. In vivo chimpanzee data are available from the National Chimpanzee Brain Resource (www.chimpanzeebrain.org). IFOF, inferior fronto-occipital fascicle; ILFlat, inferior longitudinal fascicle lateral; ILFmed, inferior longitudinal fascicle medial; MdLF, middle longitudinal fascicle.
(TIF)

S4 Fig AF tractography protocols and results. Top panel: AF tractography masks example for one individual macaque, human, and chimpanzee, represented on the principal eigenvector (V1) map weighted by the fractional anisotropy map. The light-pink mask represents the seed, and the white masks represent the anterior and posterior waypoints. Bottom panel: 3D and surface representation of the left tractogram obtained for AF. Threshold of 0.75. R denotes right hemisphere. Human data are available from the Human Connectome Project (www.humanconnectome.org). Macaque postmortem data are available from the PRIME-DE repository (http://fcon_1000.projects.nitrc.org/indi/PRIME/oxford2.html). In vivo chimpanzee data are available from the National Chimpanzee Brain Resource (www.chimpanzeebrain.org). AF, arcuate fascicle; PRIME-DE, Primate Data Exchange.
(TIF)

S5 Fig **MRtrix results showing tractography streamlines for temporal lobe tracts in macaques (A) humans (B).** Tractograms were log transformed and normalized for display. Blue, MdLF; green, IFOF; yellow, ILF (macaque) or ILFmed (human); red, ILFlat; pink, AF. Thresholds for the tracts are as follows: 0.82 for MDLF; 0.82 for IFOF; 0.8 for ILF, ILFmed, and ILFlat; and 0.8 for AF. Human data are available from the Human Connectome Project (www.humanconnectome.org). Macaque postmortem data are available from the PRIME-DE repository (http://fcon_1000.projects.nitrc.org/indi/PRIME/oxford2.html). AF, arcuate fascicle; IFOF, inferior fronto-occipital fascicle; ILF, inferior longitudinal fascicle; ILFlat, inferior longitudinal fascicle lateral; ILFmed, inferior longitudinal fascicle medial; MdLF, middle longitudinal fascicle; PRIME-DE, Primate Data Exchange.
(TIF)

S6 Fig Results of the blueprint analysis. The figure reports results on the left hemisphere. Shown are the tracts used (top row), the resulting KL divergence (middle row) for human predicting macaque (left) and macaque predicting human (right), and the connectivity fingerprints (bottom row). The greener the vertices of the KL divergence map in one brain, the more their connectivity profile is similar to that of vertices in the other brain. The connectivity fingerprints show the probability of the vertex, highlighted by a white sphere in the brain above, to be reached by each tract (dotted line). The solid line represents the probability of being reached by each tract in the other species, calculated as the mean over the 10 vertices with the smallest KL divergence with the initial species’ vertex of interest. (A) Blueprints established using the cbt, the fx, the or, and the unc. (B) Blueprints established with the tracts in (A) and adding the MdLF, ILF (both subcomponents combined in humans), and IFOF. (C) Blueprint established with the tracts in (B) and adding the AF. Blue, MdLF; green, IFOF; yellow, ILF (macaque) or ILFmed (human); red, ILFlat; pink, AF. (D) Distribution of minimum KL values obtained for each blueprint. From left to right for the macaque, the human, and masked with the human AF. Light gray, nonlongitudinal tracts; gray, adding MdLF, ILFs, and IFOF; black, adding AF. Human data are available from the Human Connectome Project (www.humanconnectome.org). Macaque postmortem data are available from the PRIME-DE repository (http://fcon_1000.projects.nitrc.org/indi/PRIME/oxford2.html). AF, arcuate fascicle; cbt, temporal part of the cingulum bundle; fx, fornix; IFOF, inferior fronto-occipital fascicle; ILF, inferior longitudinal fascicle; ILFlat, inferior longitudinal fascicle lateral; ILFmed, inferior longitudinal fascicle medial; KL, Kullback–Liebler; MdLF, middle longitudinal fascicle; or, optic radiation; PRIME-DE, Primate Data Exchange; unc, uncinate fascicle.
(TIF)

S7 Fig Blueprint method to determine differences between the human ILFs. **(**A) Blueprint established as in S6C Fig but with the ILFlat for humans (no ILF med). (B) Blueprint established as in (A) but with the ILFmed for humans (no ILFlat). (C) The different ILF tractograms are represented on the top row. On the bottom row is shown the KL difference between the two maps established in (A) and (B). More yellow vertices means that using ILFmed in humans as macaques’ ILF homologous results in lower KL divergence between the two species at these vertices, whereas more red applies to ILFlat. Blue, MdLF; green, IFOF; yellow, ILF (macaque) or ILFmed (human); red, ILFlat; pink, AF. (D) Distribution of minimum KL values in the macaque’s ILF territory obtained for the blueprints with the different human ILF subcomponents. Red, with human ILFlat; yellow, with human ILFmed. Human data are available from the Human Connectome Project (www.humanconnectome.org). Macaque postmortem data are available from the PRIME-DE repository (http://fcon_1000.projects.nitrc.org/indi/PRIME/oxford2.html). IFOF, inferior fronto-occipital fascicle; ILF, inferior longitudinal fascicle; ILFlat, inferior longitudinal fascicle lateral; ILFmed, inferior longitudinal fascicle medial; KL, Kullback–Liebler; MdLF, middle longitudinal fascicle; PRIME-DE, Primate Data Exchange.
(TIF)

S8 Fig Verification of cluster number reliability. (A) Illustration of the correspondence between ILF clusters in the middle (seed) ROI when defined as one ILF (three-cluster solution, top row) or the sum of the two subcomponents (four-cluster solution, bottom row), shown from the clustering results obtained across species (thresholded at two individuals’ overlap for macaques, two for chimpanzee, and four for humans). (The IFOF is not visible on the macaque left hemisphere four-cluster solution because the hypothetical ILF subcomponents are invading that space.) R denotes right hemisphere. (B) Correspondence between ILF as defined as a single cluster in the three-cluster solution and as defined by the sum of two clusters in the four-cluster solution, shown as their percentage overlap. If the ILF is reliably split into subclusters, the “correspondence” should be high. (C) Hierarchy index between the three-cluster and four-cluster solutions is represented in black squares for all species. The black lines represent the range of hierarchy index values obtained with 1,000 random permutations of cluster labeling. (D) Results from all measures investigating cluster number solutions. The y-axis represents the results of the subtraction of macaques’ ideal cluster number from the great apes’ cluster number. Each symbol corresponds to a different measure. Human data are available from the Human Connectome Project (www.humanconnectome.org). Macaque postmortem data are available from the PRIME-DE repository (http://fcon_1000.projects.nitrc.org/indi/PRIME/oxford2.html). In vivo chimpanzee data are available from the National Chimpanzee Brain Resource (www.chimpanzeebrain.org). Gorilla postmortem data are available from https://doi.org/10.5281/zenodo.3901205. IFOF, inferior fronto-occipital fascicle; ILF, inferior longitudinal fascicle; ILFlat, inferior longitudinal fascicle lateral; ILFmed, inferior longitudinal fascicle medial; PRIME-DE, Primate Data Exchange.
(TIF)

S9 Fig Voxel distributions in the three- to four-cluster solutions in the middle ROI. The lines link the clusters between the two clustering solutions according to whether they have voxels in common; the thickness of the line represents the percentage of the three-cluster voxels that have been allocated to the four clusters. In humans, the clear division of the ILF in the three-cluster solution into ILFmed and ILFlat in the four-cluster solution can be observed. This division is not apparent in macaques. Human data are available from the Human Connectome Project (www.humanconnectome.org). Macaque postmortem data are available from the PRIME-DE repository (http://fcon_1000.projects.nitrc.org/indi/PRIME/oxford2.html). IFOF, inferior fronto-occipital fascicle; ILF, inferior longitudinal fascicle; ILFlat, inferior longitudinal fascicle lateral; ILFmed, inferior longitudinal fascicle medial; MdLF, middle longitudinal fascicle; PRIME-DE, Primate Data Exchange.
(TIF)

S10 Fig FMRIB Software Library probabilistic and deterministic clustering results. The top panel shows the Dice coefficients in black dots between the clustering results obtained with probabilistic and deterministic tractography for each ROI. The light-gray lines represent the range of Dice coefficients obtained between 1,000 random permutations of cluster solutions. The bottom panels show the clustering results obtained with probabilistic (top) and deterministic (bottom) tractography for each ROI. As in Fig 10 and Fig 11, the clustering results are thresholded to show the overlap between at least two subjects out of four in macaques and four out of 10 in humans; no thresholding is applied in chimpanzees and gorilla. R denotes right hemisphere. Human data are available from the Human Connectome Project (www.humanconnectome.org). Macaque postmortem data are available from the PRIME-DE repository (http://fcon_1000.projects.nitrc.org/indi/PRIME/oxford2.html). In vivo chimpanzee data are available from the National Chimpanzee Brain Resource (www.chimpanzeebrain.org). Gorilla postmortem data are available from https://doi.org/10.5281/zenodo.3901205. ant, anterior; post, posterior; PRIME-DE, Primate Data Exchange; ROI, region of interest.
(TIF)

S11 Fig Comparison of tract reconstruction using different tractography techniques in macaques. (A) Comparison of tract anatomy obtained using FSL probabilistic, FSL deterministic, and MRtrix probabilistic techniques, as well as overlapping tracts between the three. The top panels show the 3D representation of the tracts, and the bottom ones show their organization on coronal sections. Thresholds for the tracts with FSL probabilistic and deterministic techniques are as follows: 0.7 for MDLF; 0.75 for IFOF; 0.7 for ILF; and 0.75 for AF. Thresholds for the tracts with MRtrix are as follows: 0.82 for MDLF; 0.82 for IFOF; 0.8 for ILF; and 0.8 for AF. The overlap is binarized. (B) Comparison of tract overlap when reconstructed with different techniques. We calculated the number of voxels overlapping between a tract of interest and each of the tracts obtained with FSL probabilistic divided by the total number of voxels of the tract of interest. The darker color represents the overlap of the tract of interest obtained with FSL probabilistic, and the lighter color represents the overlap of the tract of interest obtained with MRtrix and the color in between the tract of interest obtained with FSL deterministic. Macaque postmortem data are available from the PRIME-DE repository (http://fcon_1000.projects.nitrc.org/indi/PRIME/oxford2.html). AF, arcuate fascicle; FSL, FMRIB Software Library; IFOF, inferior fronto-occipital fascicle; ILF, inferior longitudinal fascicle; ILFmed, inferior longitudinal fascicle medial; MdLF, middle longitudinal fascicle; PRIME-DE, Primate Data Exchange.
(TIF)

S12 Fig Comparison of tract reconstruction using different tractography techniques in humans. (A) Comparison of tract anatomy obtained using FSL probabilistic, FSL deterministic, and MRtrix probabilistic techniques, as well as overlapping tracts between the three. The top panels show the 3D representation of the tracts, and the bottom ones show their organization on coronal sections. Thresholds for the tracts with FSL probabilistic and deterministic techniques are as follows: 0.7 for MDLF; 0.75 for IFOF; 0.7 for ILFlat and ILFmed; and 0.75 for AF. Thresholds for the tracts with MRtrix are as follows: 0.82 for MDLF; 0.82 for IFOF; 0.8 for ILFlat and ILFmed; and 0.8 for AF. The overlap is binarized. (B) Comparison of tract overlap when reconstructed with different techniques. We calculated the number of voxels overlapping between a tract of interest and each of the tracts obtained with FSL probabilistic divided by the total number of voxels of the tract of interest. The darker color represents the overlap of the tract of interest obtained with FSL probabilistic, and the lighter color represents the overlap of the tract of interest obtained with MRtrix and the color in between the tract of interest obtained with FSL deterministic. Macaque *postmortem* data are available from the PRIME-DE repository (http://fcon_1000.projects.nitrc.org/indi/PRIME/oxford2.html). AF, arcuate fascicle; FSL, FMRIB Software Library; IFOF, inferior fronto-occipital fascicle; ILFlat, inferior longitudinal fascicle lateral; ILFmed, inferior longitudinal fascicle medial; MdLF, middle longitudinal fascicle; PRIME-DE, Primate Data Exchange.
(TIF)

S13 Fig Comparison of tract reconstruction using different tractography techniques in chimpanzees in vivo. (A) Comparison of tract anatomy obtained using FSL probabilistic and FSL deterministic techniques, as well as overlapping tracts between the two. The top panels show the 3D representation of the tracts, and the bottom ones show their organization on coronal sections. Thresholds for the tracts with FSL probabilistic and deterministic techniques are as follows: 0.7 for MDLF; 0.75 for IFOF; 0.7 for ILFlat and ILFmed; and 0.75 for AF. The overlap is binarized. (B) Comparison of tract overlap when reconstructed with different techniques. We calculated the number of voxels overlapping between a tract of interest and each of the tracts obtained with FSL probabilistic divided by the total number of voxels of the tract of interest. The darker color represents the overlap of the tract of interest obtained with FSL probabilistic, and the lighter color represents the overlap of the tract of interest obtained with FSL deterministic. In vivo chimpanzee data are available from the National Chimpanzee Brain Resource (www.chimpanzeebrain.org). AF, arcuate fascicle; FSL, FMRIB Software Library; IFOF, inferior fronto-occipital fascicle; ILFlat, inferior longitudinal fascicle lateral; ILFmed, inferior longitudinal fascicle medial; MdLF, middle longitudinal fascicle.
(TIF)

S14 Fig Comparison of tract reconstruction using different tractography techniques in gorilla postmortem. (A) Comparison of tract anatomy obtained using FSL probabilistic and FSL deterministic techniques, as well as overlapping tracts between the two. The top panels show the 3D representation of the tracts, and the bottom ones show their organization on coronal sections. Thresholds for the tracts with FSL probabilistic and deterministic techniques are as follows: 0.7 for MDLF; 0.75 for IFOF; 0.7 for ILFlat and ILFmed; and 0.75 for AF. The overlap is binarized. (B) Comparison of tract overlap when reconstructed with different techniques. We calculated the number of voxels overlapping between a tract of interest and each of the tracts obtained with FSL probabilistic divided by the total number of voxels of the tract of interest. The darker color represents the overlap of the tract of interest obtained with FSL probabilistic, and the lighter color represents the overlap of the tract of interest obtained with FSL deterministic. Gorilla postmortem data are available from https://doi.org/10.5281/zenodo.3901205. AF, arcuate fascicle; FSL, FMRIB Software Library; IFOF, inferior fronto-occipital fascicle; ILFlat, inferior longitudinal fascicle lateral; ILFmed, inferior longitudinal fascicle medial; MdLF, middle longitudinal fascicle.
(TIF)

S1 Text Tractography as the best method to study white matter in a large range of species. (DOCX)

S2 Text Additional methods. (DOCX)

## Abbreviations

AF: arcuate fascicle
FFA: fusiform face area
IFOF: inferior fronto-occipital fascicle
ILF: inferior longitudinal fascicle
ILFlat: ILF lateral
ILFmed: ILF medial
KL: Kullback–Liebler
MdLF: middle longitudinal fascicle
MRI: magnetic resonance imaging
ROI: region of interest
