# Supplementary figures and images for: Longitudinal connections and the organization of the temporal cortex in macaques, great apes, and humans
Source: PLoS Biol. 2020 Jul 31;18(7):e3000810. doi: 10.1371/journal.pbio.3000810 (PMC7423156; doi:10.1371/journal.pbio.3000810)

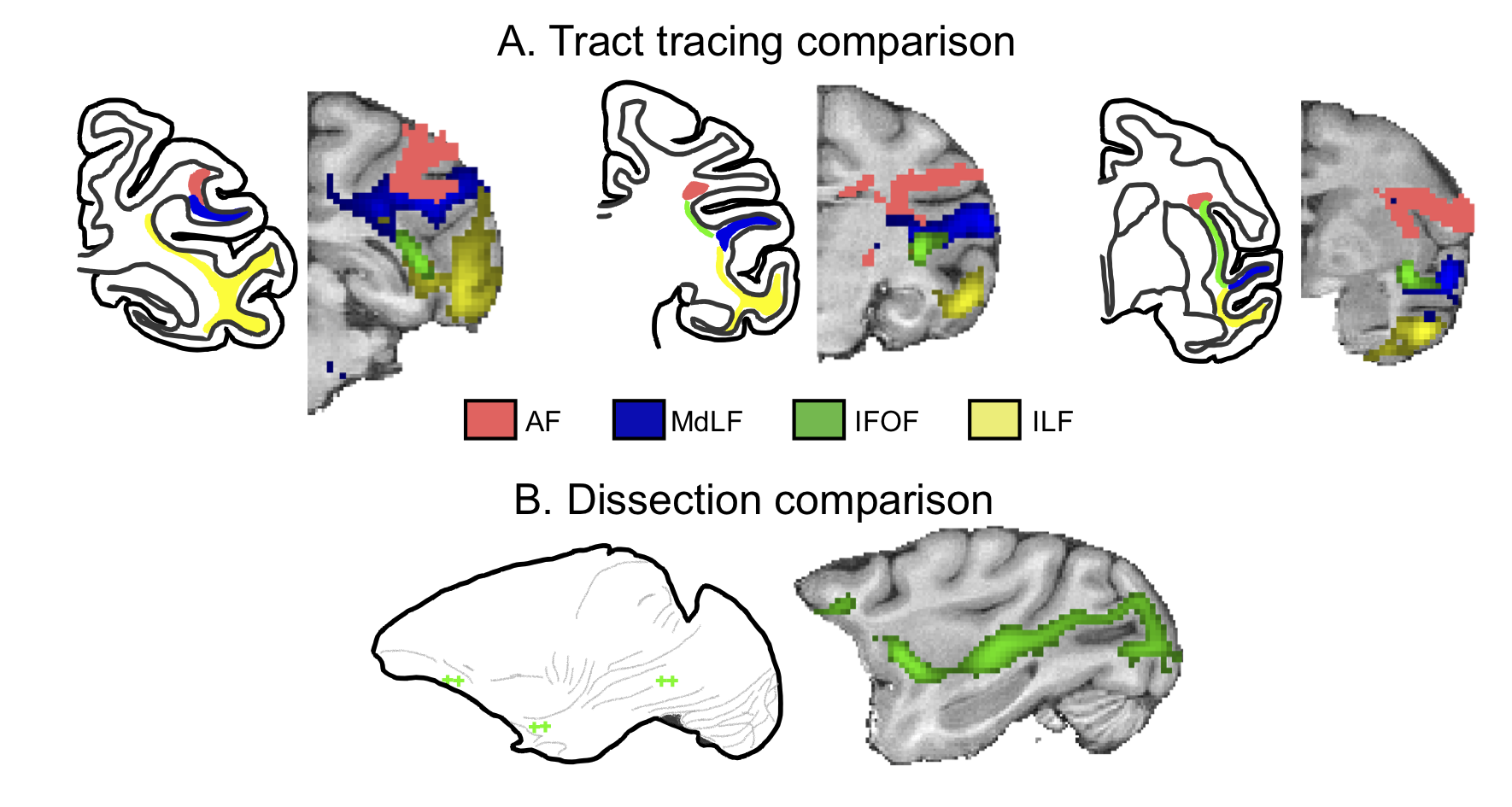

Supplement: S1 Fig — (A) Comparison with tract tracing results redrawn from Schmahmann and Pandya (2006) [29]. Tracts to be compared are highlighted in similar colors. Blue, MdLF; green, IFOF; yellow, ILF; pink, AF. (B) Comparison with the dissection results redrawn from Decramer and colleagues (2018) [23]; the green crosses illustrate the IFOF pathway. Macaque postmortem data are available from the PRIME-DE repository (http://fcon_1000.projects.nitrc.org/indi/PRIME/oxford2.html). AF, arcuate fascicle; IFOF, inferior fronto-occipital fascicle; ILF, inferior longitudinal fascicle; MdLF, middle longitudinal fascicle; PRIME-DE, Primate Data Exchange. (TIF) [file pbio.3000810.s001.tif]

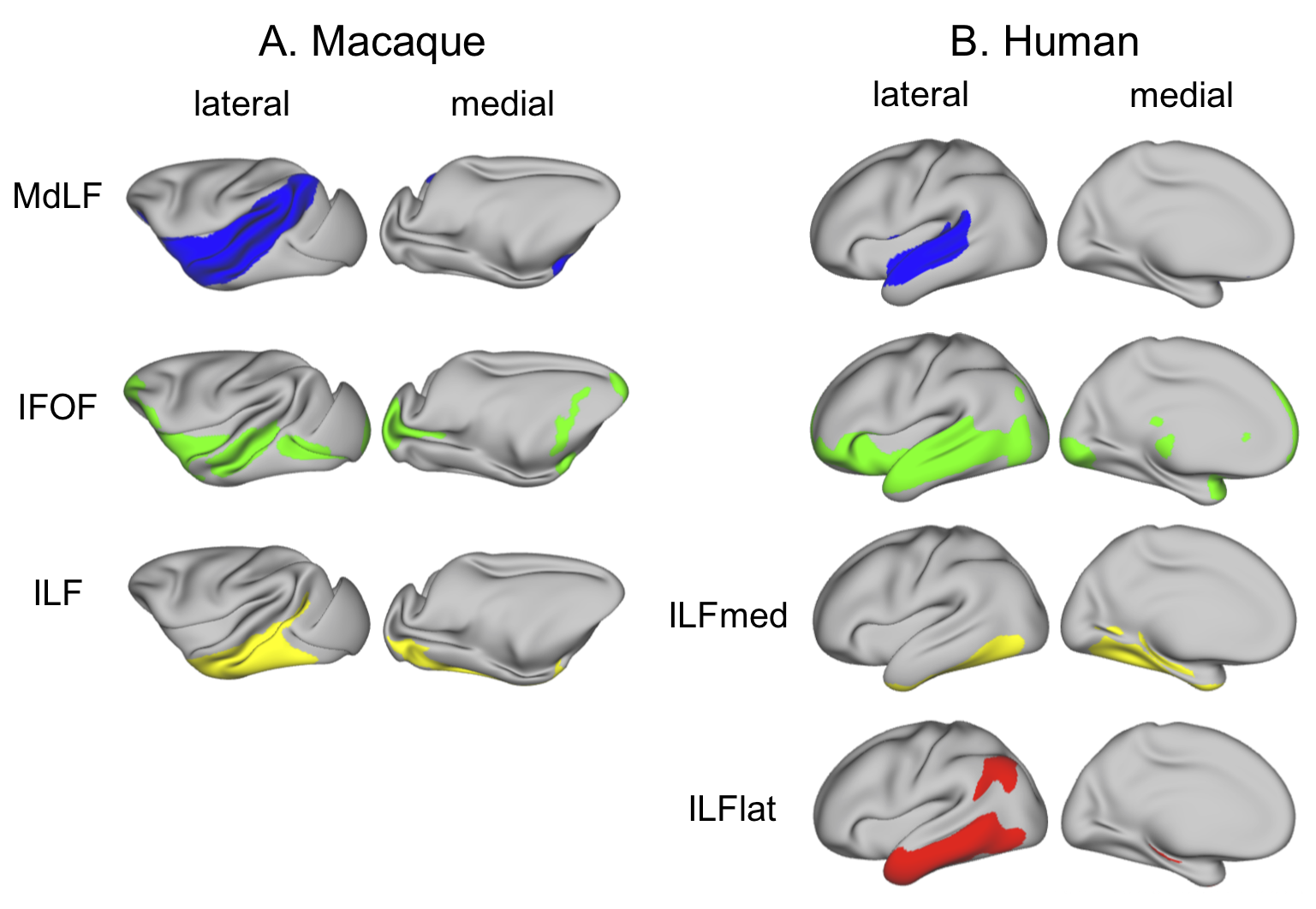

Supplement: S2 Fig — Surface projection of longitudinal temporal tracts in macaques (A) and humans (B). Shown are the group averages of the normalized, log-transformed, and thresholded tracts in the left hemisphere only (for space-saving purpose). Blue, MdLF; green, IFOF; yellow, ILF (macaque) or ILFmed (humans); red, ILFlat. Thresholds for the tracts are as follows: 0.7 for MDLF; 0.75 for IFOF; and 0.7 for ILF, ILFmed, and ILFlat. Human data are available from the Human Connectome Project (www.humanconnectome.org). Macaque postmortem data are available from the PRIME-DE repository (http://fcon_1000.projects.nitrc.org/indi/PRIME/oxford2.html). IFOF, inferior fronto-occipital fascicle; ILF, inferior longitudinal fascicle; ILFlat, inferior longitudinal fascicle lateral; ILFmed, inferior longitudinal fascicle medial; MdLF, middle longitudinal fascicle; PRIME-DE, Primate Data Exchange. (TIF) [file pbio.3000810.s002.tif]

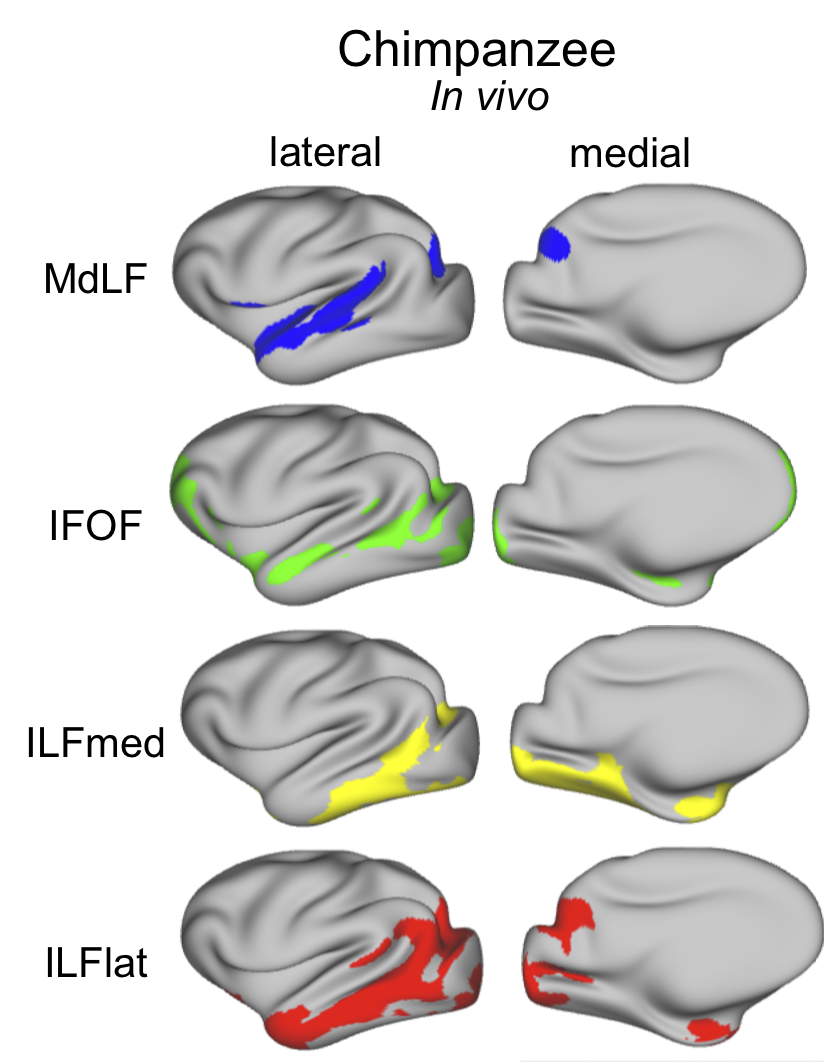

Supplement: S3 Fig — Shown are the group averages of the normalized, log-transformed, smoothed and thresholded left tracts. Blue, MdLF; green, IFOF; yellow, ILFmed; red, ILFlat. Thresholds for the tracts are as follows: 0.7 for MDLF; 0.75 for IFOF; and 0.7 for ILFmed and ILFlat. In vivo chimpanzee data are available from the National Chimpanzee Brain Resource (www.chimpanzeebrain.org). IFOF, inferior fronto-occipital fascicle; ILFlat, inferior longitudinal fascicle lateral; ILFmed, inferior longitudinal fascicle medial; MdLF, middle longitudinal fascicle. (TIF) [file pbio.3000810.s003.tif]

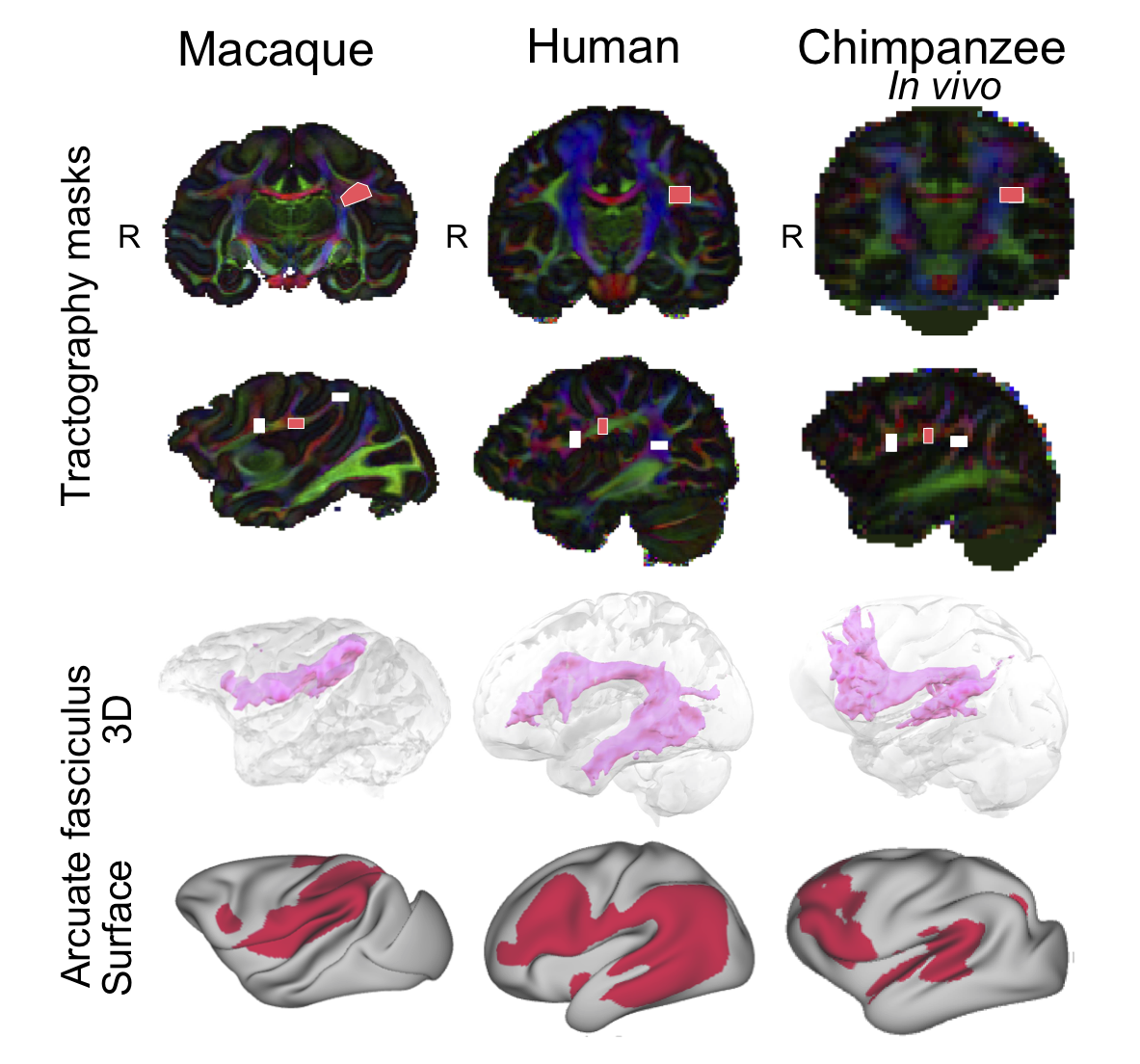

Supplement: S4 Fig — Top panel: AF tractography masks example for one individual macaque, human, and chimpanzee, represented on the principal eigenvector (V1) map weighted by the fractional anisotropy map. The light-pink mask represents the seed, and the white masks represent the anterior and posterior waypoints. Bottom panel: 3D and surface representation of the left tractogram obtained for AF. Threshold of 0.75. R denotes right hemisphere. Human data are available from the Human Connectome Project (www.humanconnectome.org). Macaque postmortem data are available from the PRIME-DE repository (http://fcon_1000.projects.nitrc.org/indi/PRIME/oxford2.html). In vivo chimpanzee data are available from the National Chimpanzee Brain Resource (www.chimpanzeebrain.org). AF, arcuate fascicle; PRIME-DE, Primate Data Exchange. (TIF) [file pbio.3000810.s004.tif]

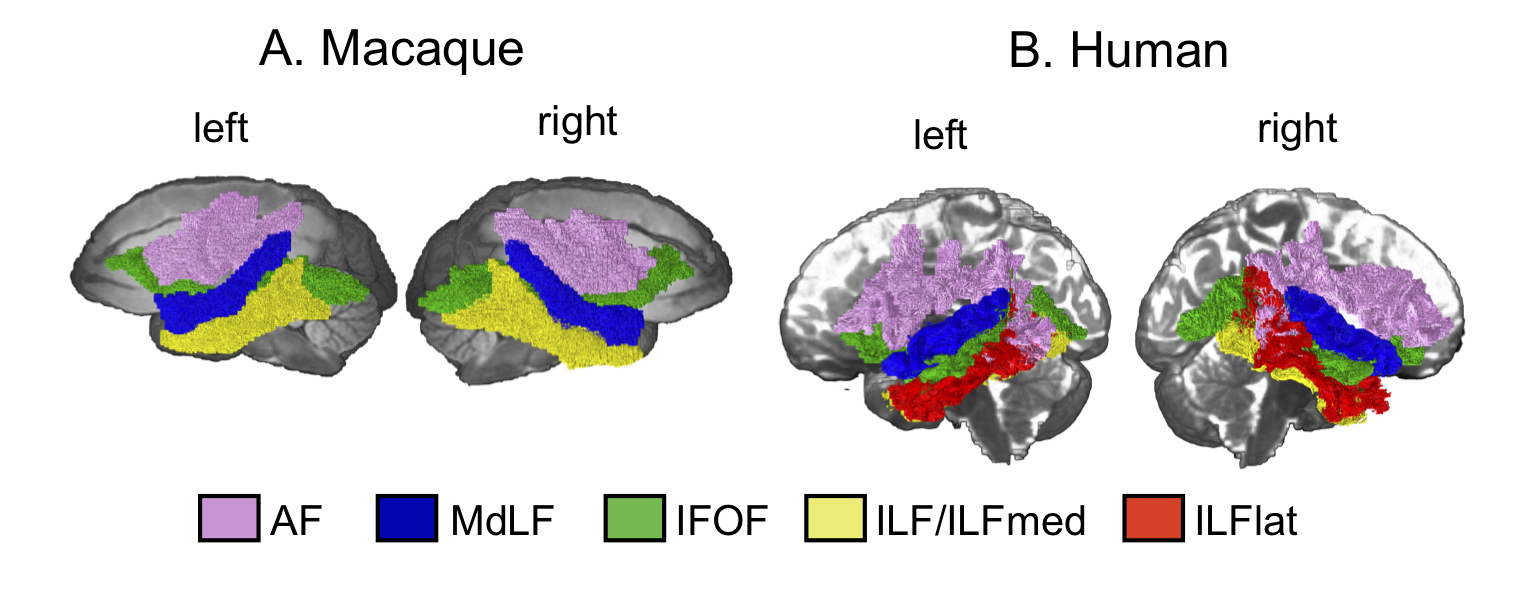

Supplement: S5 Fig — MRtrix results showing tractography streamlines for temporal lobe tracts in macaques (A) humans (B). Tractograms were log transformed and normalized for display. Blue, MdLF; green, IFOF; yellow, ILF (macaque) or ILFmed (human); red, ILFlat; pink, AF. Thresholds for the tracts are as follows: 0.82 for MDLF; 0.82 for IFOF; 0.8 for ILF, ILFmed, and ILFlat; and 0.8 for AF. Human data are available from the Human Connectome Project (www.humanconnectome.org). Macaque postmortem data are available from the PRIME-DE repository (http://fcon_1000.projects.nitrc.org/indi/PRIME/oxford2.html). AF, arcuate fascicle; IFOF, inferior fronto-occipital fascicle; ILF, inferior longitudinal fascicle; ILFlat, inferior longitudinal fascicle lateral; ILFmed, inferior longitudinal fascicle medial; MdLF, middle longitudinal fascicle; PRIME-DE, Primate Data Exchange. (TIF) [file pbio.3000810.s005.tif]

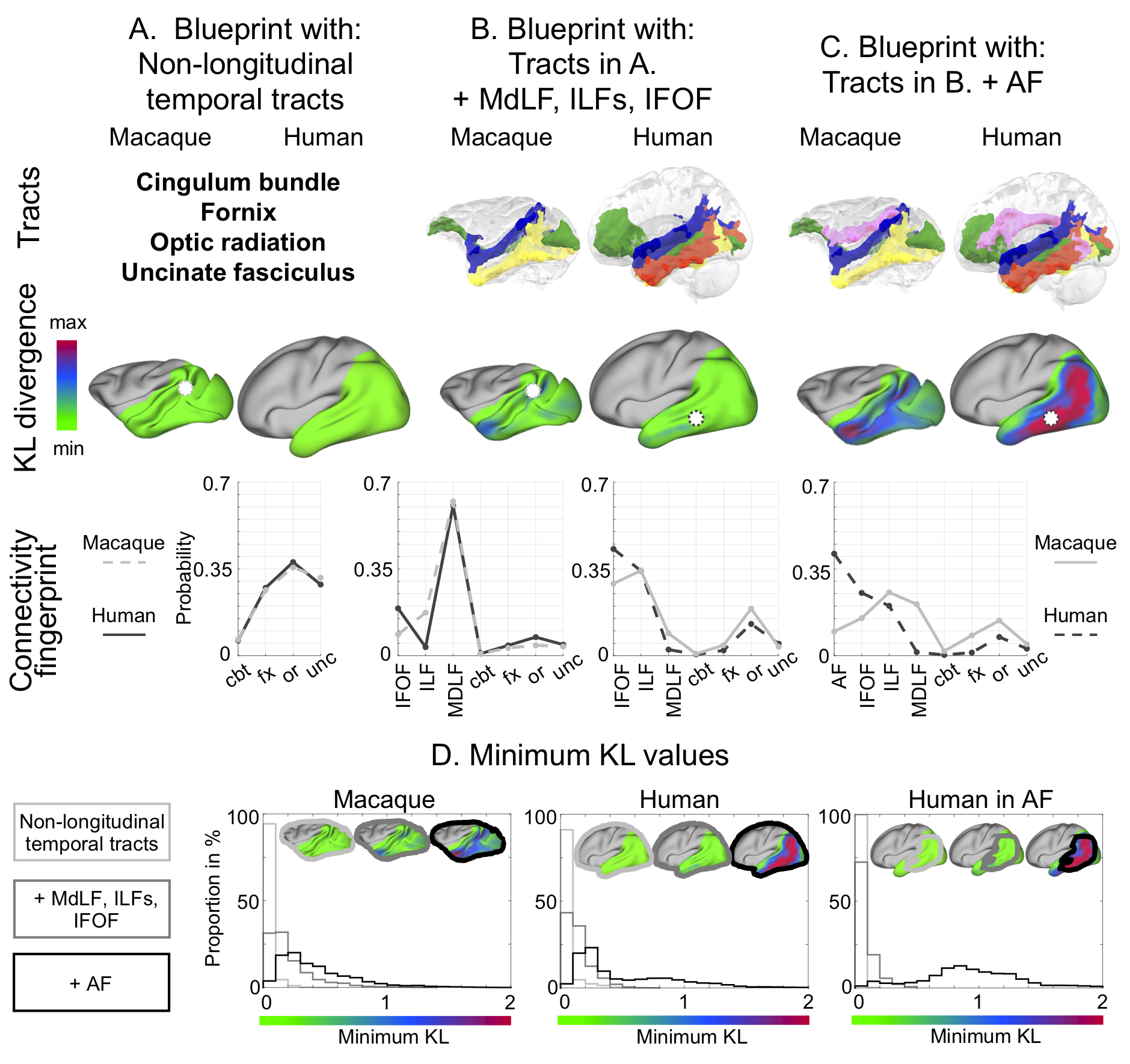

Supplement: S6 Fig — The figure reports results on the left hemisphere. Shown are the tracts used (top row), the resulting KL divergence (middle row) for human predicting macaque (left) and macaque predicting human (right), and the connectivity fingerprints (bottom row). The greener the vertices of the KL divergence map in one brain, the more their connectivity profile is similar to that of vertices in the other brain. The connectivity fingerprints show the probability of the vertex, highlighted by a white sphere in the brain above, to be reached by each tract (dotted line). The solid line represents the probability of being reached by each tract in the other species, calculated as the mean over the 10 vertices with the smallest KL divergence with the initial species’ vertex of interest. (A) Blueprints established using the cbt, the fx, the or, and the unc. (B) Blueprints established with the tracts in (A) and adding the MdLF, ILF (both subcomponents combined in humans), and IFOF. (C) Blueprint established with the tracts in (B) and adding the AF. Blue, MdLF; green, IFOF; yellow, ILF (macaque) or ILFmed (human); red, ILFlat; pink, AF. (D) Distribution of minimum KL values obtained for each blueprint. From left to right for the macaque, the human, and masked with the human AF. Light gray, nonlongitudinal tracts; gray, adding MdLF, ILFs, and IFOF; black, adding AF. Human data are available from the Human Connectome Project (www.humanconnectome.org). Macaque postmortem data are available from the PRIME-DE repository (http://fcon_1000.projects.nitrc.org/indi/PRIME/oxford2.html). AF, arcuate fascicle; cbt, temporal part of the cingulum bundle; fx, fornix; IFOF, inferior fronto-occipital fascicle; ILF, inferior longitudinal fascicle; ILFlat, inferior longitudinal fascicle lateral; ILFmed, inferior longitudinal fascicle medial; KL, Kullback–Liebler; MdLF, middle longitudinal fascicle; or, optic radiation; PRIME-DE, Primate Data Exchange; unc, uncinate fascicle. (TIF) [file pbio.3000810.s006.tif]

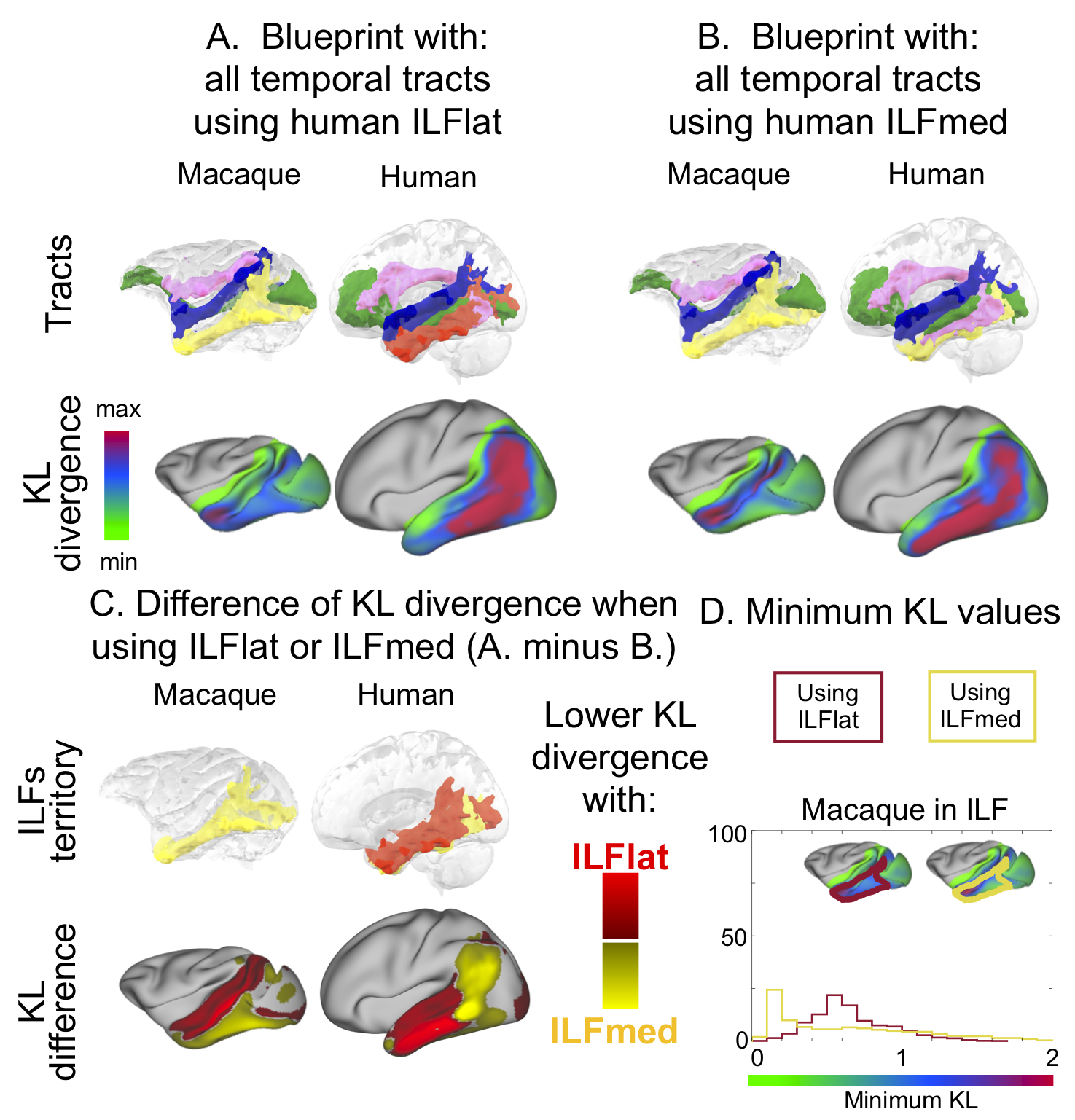

Supplement: S7 Fig — (A) Blueprint established as in S6C Fig but with the ILFlat for humans (no ILF med). (B) Blueprint established as in (A) but with the ILFmed for humans (no ILFlat). (C) The different ILF tractograms are represented on the top row. On the bottom row is shown the KL difference between the two maps established in (A) and (B). More yellow vertices means that using ILFmed in humans as macaques’ ILF homologous results in lower KL divergence between the two species at these vertices, whereas more red applies to ILFlat. Blue, MdLF; green, IFOF; yellow, ILF (macaque) or ILFmed (human); red, ILFlat; pink, AF. (D) Distribution of minimum KL values in the macaque’s ILF territory obtained for the blueprints with the different human ILF subcomponents. Red, with human ILFlat; yellow, with human ILFmed. Human data are available from the Human Connectome Project (www.humanconnectome.org). Macaque postmortem data are available from the PRIME-DE repository (http://fcon_1000.projects.nitrc.org/indi/PRIME/oxford2.html). IFOF, inferior fronto-occipital fascicle; ILF, inferior longitudinal fascicle; ILFlat, inferior longitudinal fascicle lateral; ILFmed, inferior longitudinal fascicle medial; KL, Kullback–Liebler; MdLF, middle longitudinal fascicle; PRIME-DE, Primate Data Exchange. (TIF) [file pbio.3000810.s007.tif]

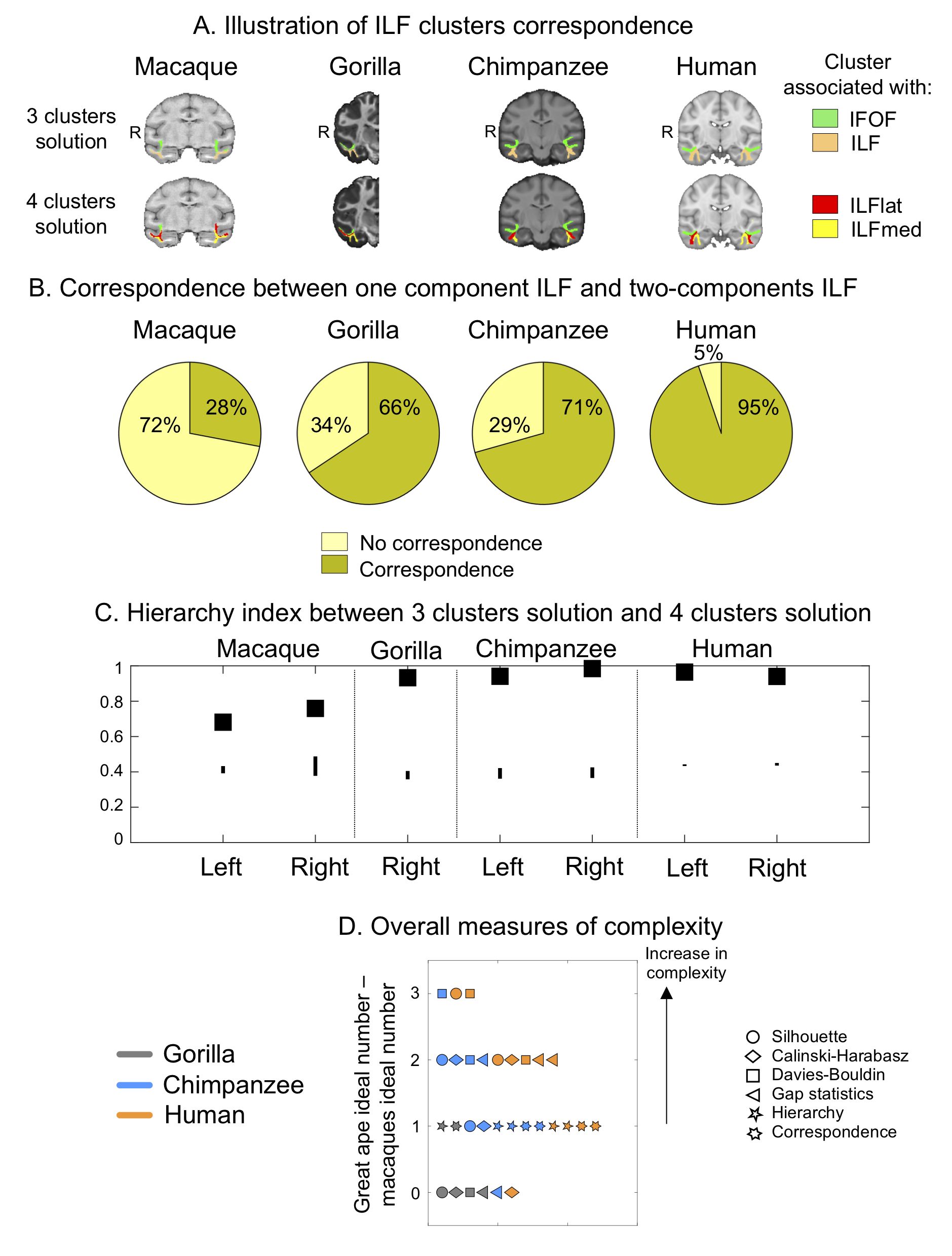

Supplement: S8 Fig — (A) Illustration of the correspondence between ILF clusters in the middle (seed) ROI when defined as one ILF (three-cluster solution, top row) or the sum of the two subcomponents (four-cluster solution, bottom row), shown from the clustering results obtained across species (thresholded at two individuals’ overlap for macaques, two for chimpanzee, and four for humans). (The IFOF is not visible on the macaque left hemisphere four-cluster solution because the hypothetical ILF subcomponents are invading that space.) R denotes right hemisphere. (B) Correspondence between ILF as defined as a single cluster in the three-cluster solution and as defined by the sum of two clusters in the four-cluster solution, shown as their percentage overlap. If the ILF is reliably split into subclusters, the “correspondence” should be high. (C) Hierarchy index between the three-cluster and four-cluster solutions is represented in black squares for all species. The black lines represent the range of hierarchy index values obtained with 1,000 random permutations of cluster labeling. (D) Results from all measures investigating cluster number solutions. The y-axis represents the results of the subtraction of macaques’ ideal cluster number from the great apes’ cluster number. Each symbol corresponds to a different measure. Human data are available from the Human Connectome Project (www.humanconnectome.org). Macaque postmortem data are available from the PRIME-DE repository (http://fcon_1000.projects.nitrc.org/indi/PRIME/oxford2.html). In vivo chimpanzee data are available from the National Chimpanzee Brain Resource (www.chimpanzeebrain.org). Gorilla postmortem data are available from https://doi.org/10.5281/zenodo.3901205. IFOF, inferior fronto-occipital fascicle; ILF, inferior longitudinal fascicle; ILFlat, inferior longitudinal fascicle lateral; ILFmed, inferior longitudinal fascicle medial; PRIME-DE, Primate Data Exchange. (TIF) [file pbio.3000810.s008.tif]

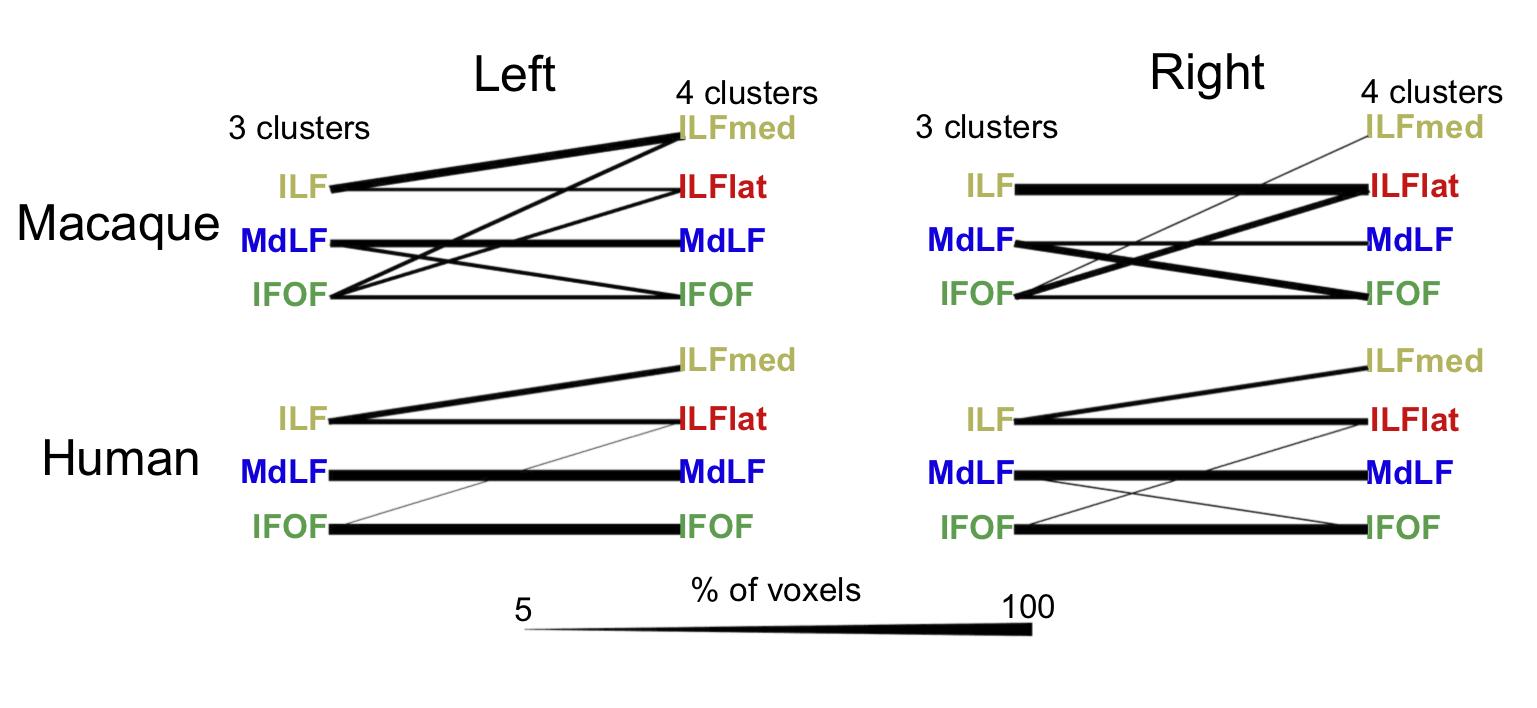

Supplement: S9 Fig — The lines link the clusters between the two clustering solutions according to whether they have voxels in common; the thickness of the line represents the percentage of the three-cluster voxels that have been allocated to the four clusters. In humans, the clear division of the ILF in the three-cluster solution into ILFmed and ILFlat in the four-cluster solution can be observed. This division is not apparent in macaques. Human data are available from the Human Connectome Project (www.humanconnectome.org). Macaque postmortem data are available from the PRIME-DE repository (http://fcon_1000.projects.nitrc.org/indi/PRIME/oxford2.html). IFOF, inferior fronto-occipital fascicle; ILF, inferior longitudinal fascicle; ILFlat, inferior longitudinal fascicle lateral; ILFmed, inferior longitudinal fascicle medial; MdLF, middle longitudinal fascicle; PRIME-DE, Primate Data Exchange. (TIF) [file pbio.3000810.s009.tif]

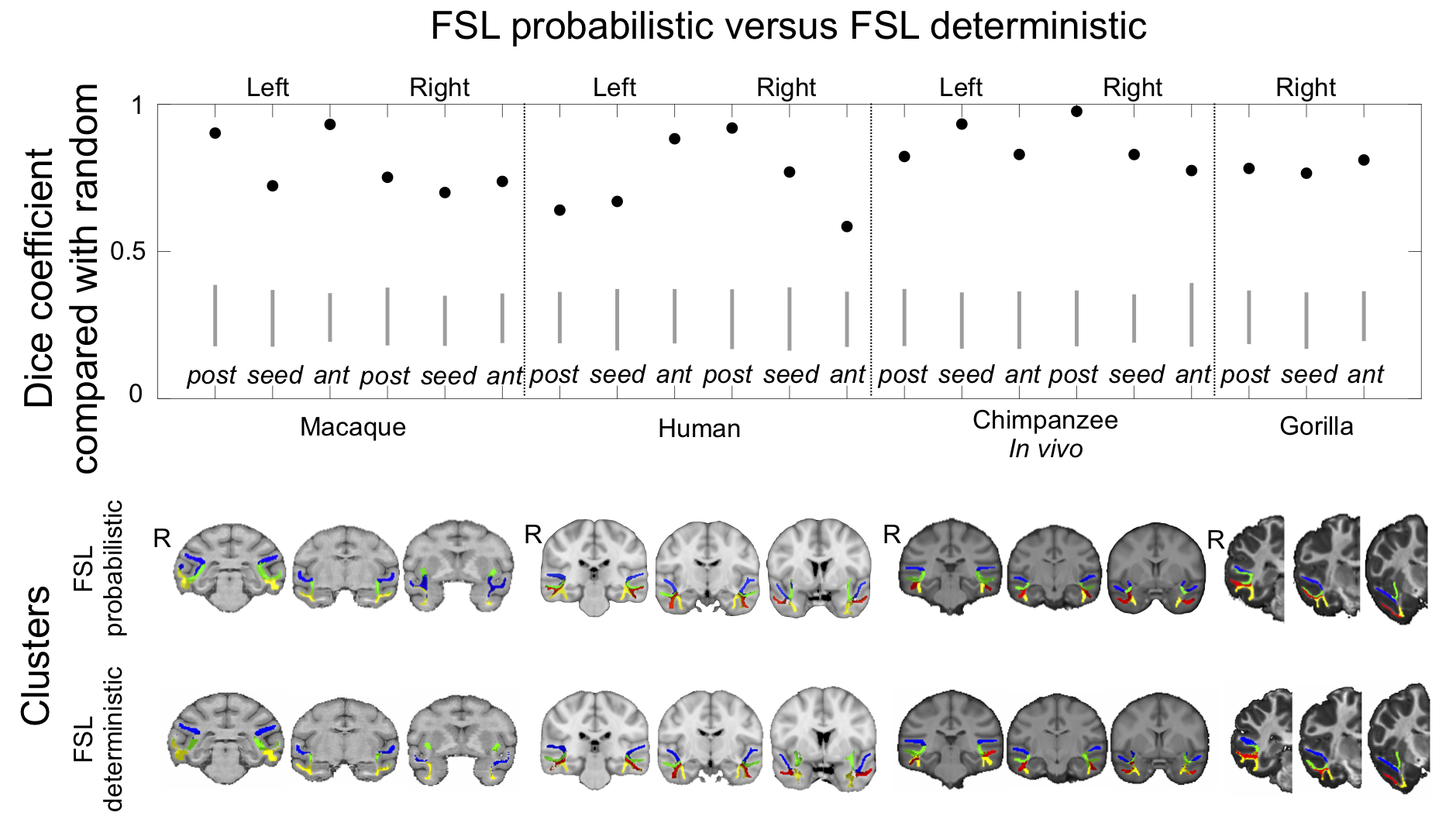

Supplement: S10 Fig — The top panel shows the Dice coefficients in black dots between the clustering results obtained with probabilistic and deterministic tractography for each ROI. The light-gray lines represent the range of Dice coefficients obtained between 1,000 random permutations of cluster solutions. The bottom panels show the clustering results obtained with probabilistic (top) and deterministic (bottom) tractography for each ROI. As in Fig 10 and Fig 11, the clustering results are thresholded to show the overlap between at least two subjects out of four in macaques and four out of 10 in humans; no thresholding is applied in chimpanzees and gorilla. R denotes right hemisphere. Human data are available from the Human Connectome Project (www.humanconnectome.org). Macaque postmortem data are available from the PRIME-DE repository (http://fcon_1000.projects.nitrc.org/indi/PRIME/oxford2.html). In vivo chimpanzee data are available from the National Chimpanzee Brain Resource (www.chimpanzeebrain.org). Gorilla postmortem data are available from https://doi.org/10.5281/zenodo.3901205. ant, anterior; post, posterior; PRIME-DE, Primate Data Exchange; ROI, region of interest. (TIF) [file pbio.3000810.s010.tif]

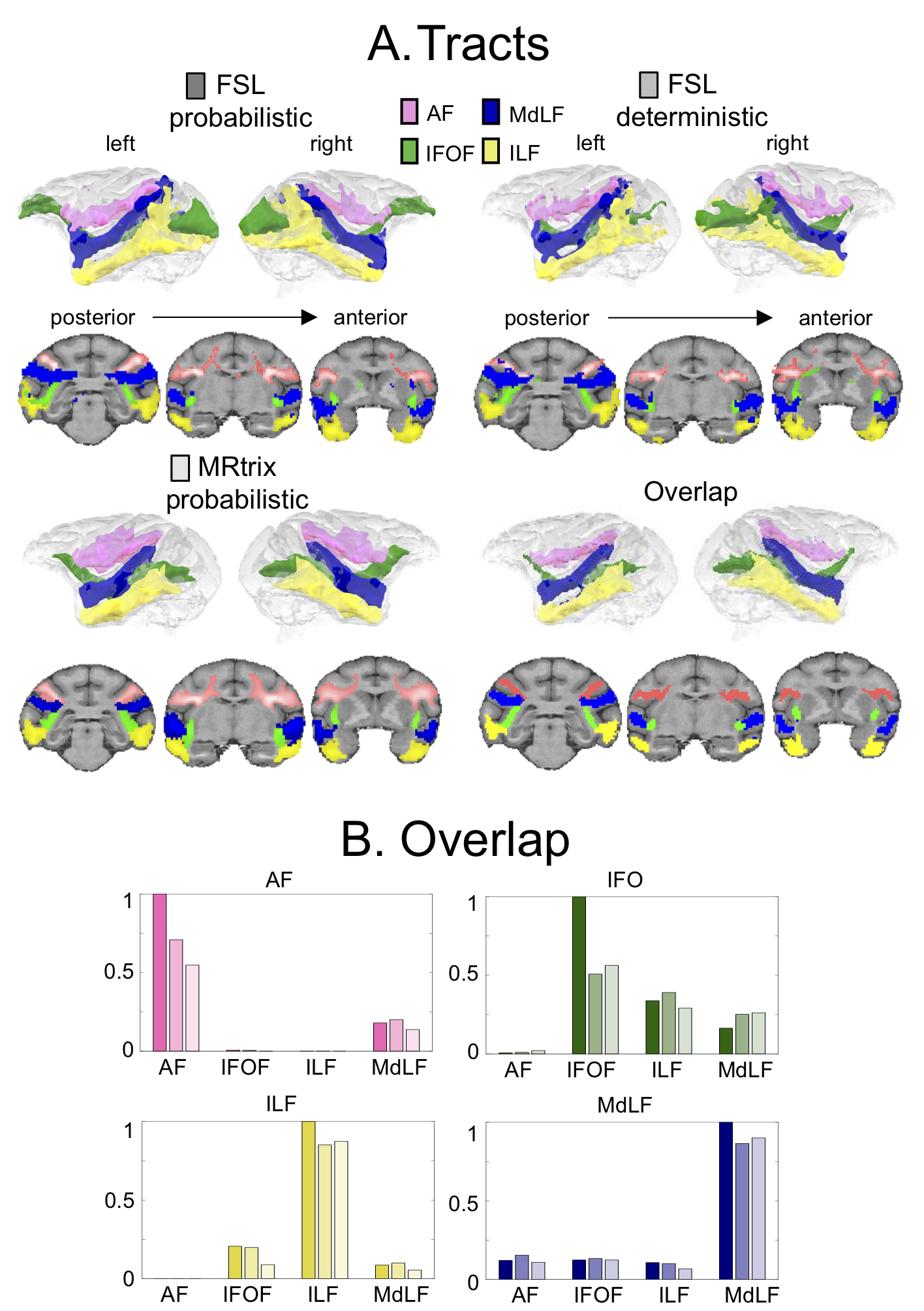

Supplement: S11 Fig — (A) Comparison of tract anatomy obtained using FSL probabilistic, FSL deterministic, and MRtrix probabilistic techniques, as well as overlapping tracts between the three. The top panels show the 3D representation of the tracts, and the bottom ones show their organization on coronal sections. Thresholds for the tracts with FSL probabilistic and deterministic techniques are as follows: 0.7 for MDLF; 0.75 for IFOF; 0.7 for ILF; and 0.75 for AF. Thresholds for the tracts with MRtrix are as follows: 0.82 for MDLF; 0.82 for IFOF; 0.8 for ILF; and 0.8 for AF. The overlap is binarized. (B) Comparison of tract overlap when reconstructed with different techniques. We calculated the number of voxels overlapping between a tract of interest and each of the tracts obtained with FSL probabilistic divided by the total number of voxels of the tract of interest. The darker color represents the overlap of the tract of interest obtained with FSL probabilistic, and the lighter color represents the overlap of the tract of interest obtained with MRtrix and the color in between the tract of interest obtained with FSL deterministic. Macaque postmortem data are available from the PRIME-DE repository (http://fcon_1000.projects.nitrc.org/indi/PRIME/oxford2.html). AF, arcuate fascicle; FSL, FMRIB Software Library; IFOF, inferior fronto-occipital fascicle; ILF, inferior longitudinal fascicle; ILFmed, inferior longitudinal fascicle medial; MdLF, middle longitudinal fascicle; PRIME-DE, Primate Data Exchange. (TIF) [file pbio.3000810.s011.tif]

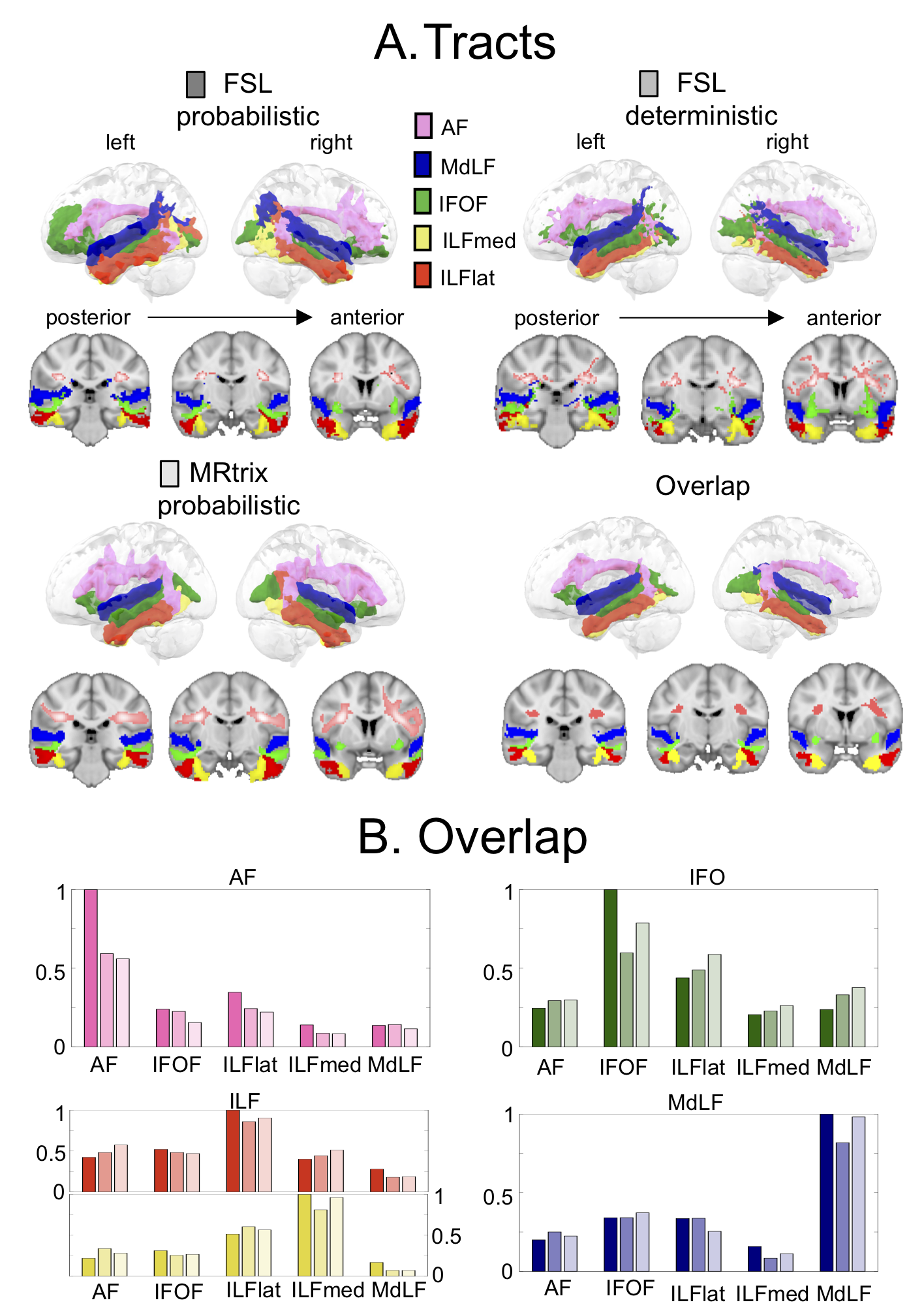

Supplement: S12 Fig — (A) Comparison of tract anatomy obtained using FSL probabilistic, FSL deterministic, and MRtrix probabilistic techniques, as well as overlapping tracts between the three. The top panels show the 3D representation of the tracts, and the bottom ones show their organization on coronal sections. Thresholds for the tracts with FSL probabilistic and deterministic techniques are as follows: 0.7 for MDLF; 0.75 for IFOF; 0.7 for ILFlat and ILFmed; and 0.75 for AF. Thresholds for the tracts with MRtrix are as follows: 0.82 for MDLF; 0.82 for IFOF; 0.8 for ILFlat and ILFmed; and 0.8 for AF. The overlap is binarized. (B) Comparison of tract overlap when reconstructed with different techniques. We calculated the number of voxels overlapping between a tract of interest and each of the tracts obtained with FSL probabilistic divided by the total number of voxels of the tract of interest. The darker color represents the overlap of the tract of interest obtained with FSL probabilistic, and the lighter color represents the overlap of the tract of interest obtained with MRtrix and the color in between the tract of interest obtained with FSL deterministic. Macaque postmortem data are available from the PRIME-DE repository (http://fcon_1000.projects.nitrc.org/indi/PRIME/oxford2.html). AF, arcuate fascicle; FSL, FMRIB Software Library; IFOF, inferior fronto-occipital fascicle; ILFlat, inferior longitudinal fascicle lateral; ILFmed, inferior longitudinal fascicle medial; MdLF, middle longitudinal fascicle; PRIME-DE, Primate Data Exchange. (TIF) [file pbio.3000810.s012.tif]

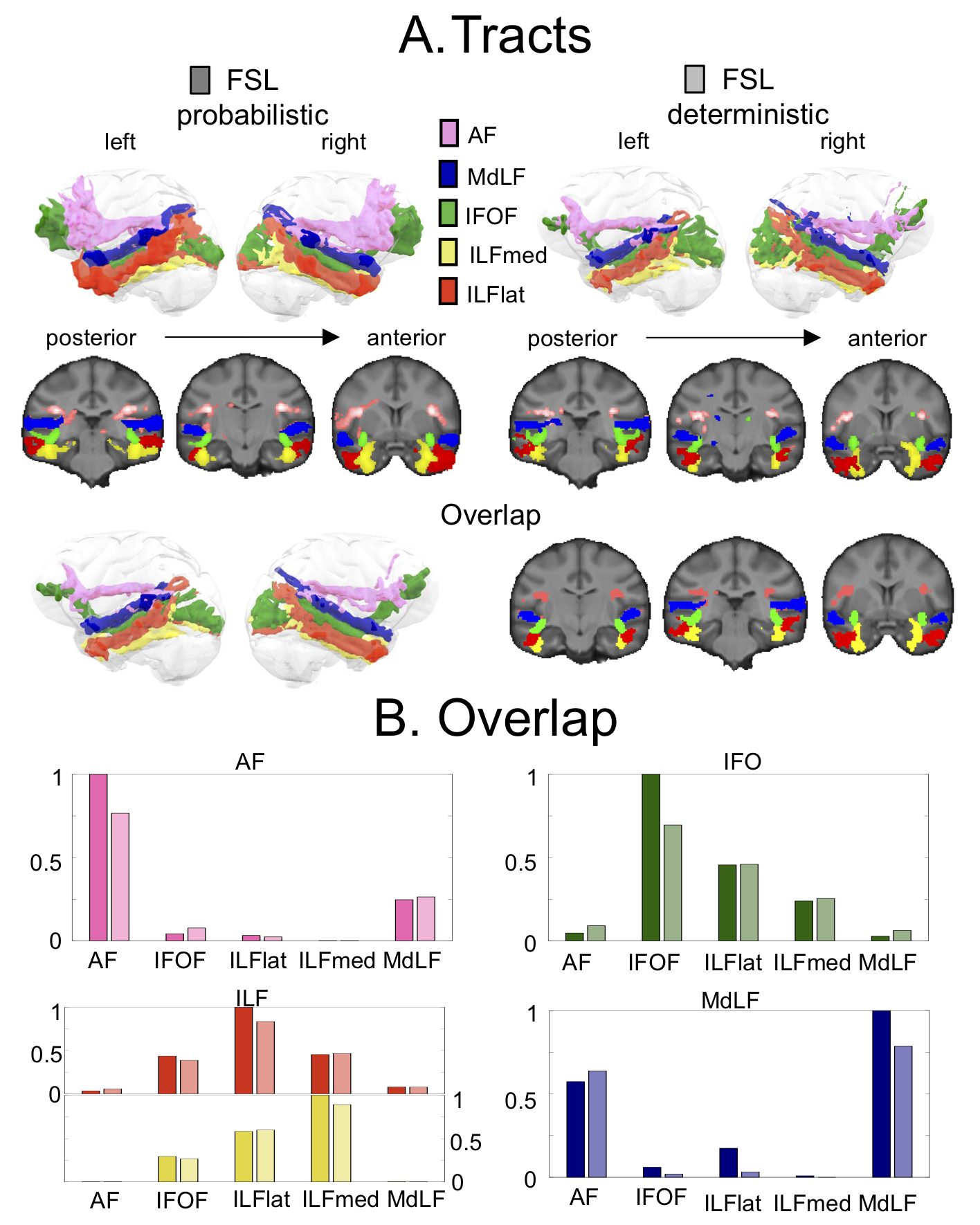

Supplement: S13 Fig — (A) Comparison of tract anatomy obtained using FSL probabilistic and FSL deterministic techniques, as well as overlapping tracts between the two. The top panels show the 3D representation of the tracts, and the bottom ones show their organization on coronal sections. Thresholds for the tracts with FSL probabilistic and deterministic techniques are as follows: 0.7 for MDLF; 0.75 for IFOF; 0.7 for ILFlat and ILFmed; and 0.75 for AF. The overlap is binarized. (B) Comparison of tract overlap when reconstructed with different techniques. We calculated the number of voxels overlapping between a tract of interest and each of the tracts obtained with FSL probabilistic divided by the total number of voxels of the tract of interest. The darker color represents the overlap of the tract of interest obtained with FSL probabilistic, and the lighter color represents the overlap of the tract of interest obtained with FSL deterministic. In vivo chimpanzee data are available from the National Chimpanzee Brain Resource (www.chimpanzeebrain.org). AF, arcuate fascicle; FSL, FMRIB Software Library; IFOF, inferior fronto-occipital fascicle; ILFlat, inferior longitudinal fascicle lateral; ILFmed, inferior longitudinal fascicle medial; MdLF, middle longitudinal fascicle. (TIF) [file pbio.3000810.s013.tif]

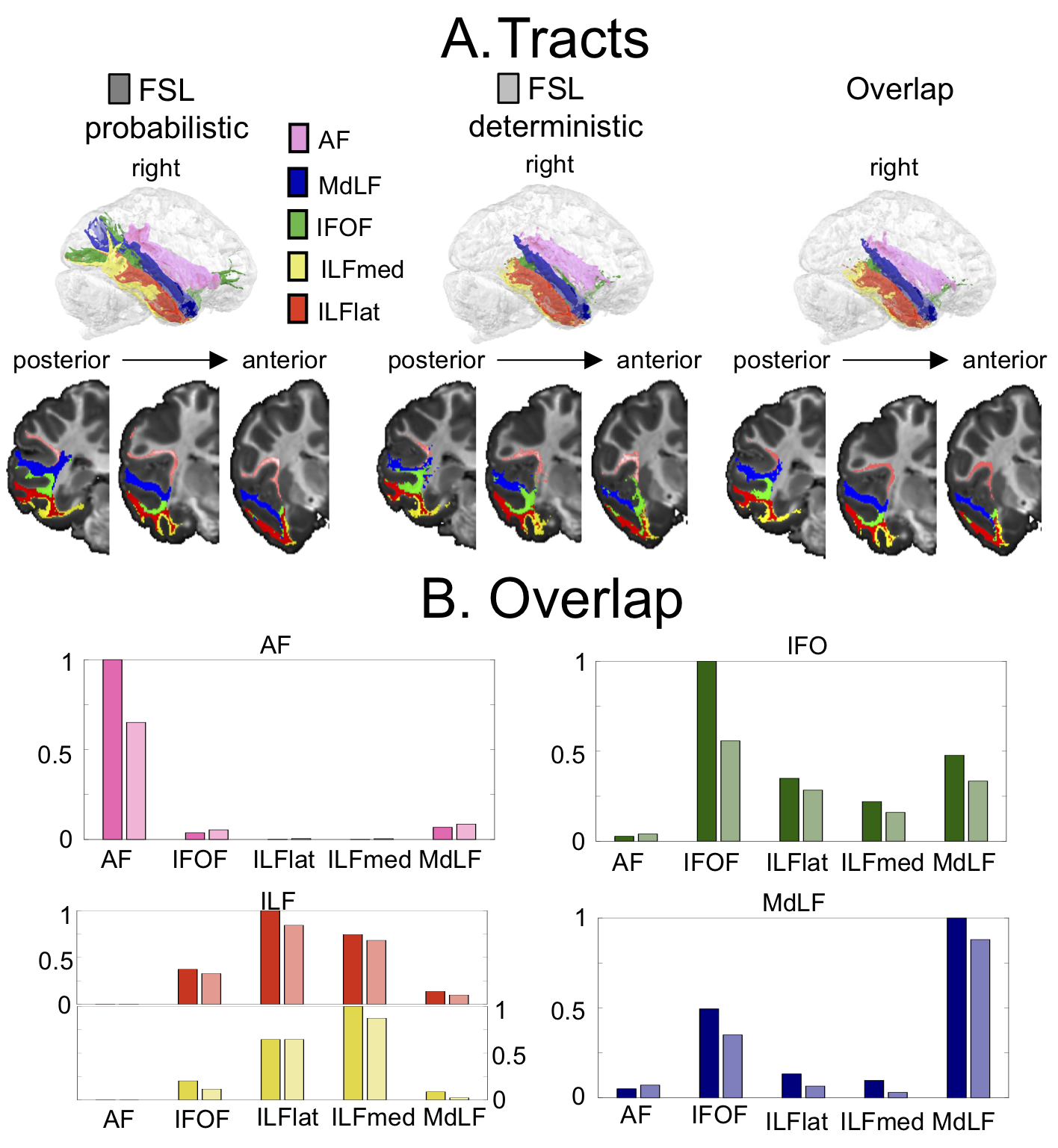

Supplement: S14 Fig — (A) Comparison of tract anatomy obtained using FSL probabilistic and FSL deterministic techniques, as well as overlapping tracts between the two. The top panels show the 3D representation of the tracts, and the bottom ones show their organization on coronal sections. Thresholds for the tracts with FSL probabilistic and deterministic techniques are as follows: 0.7 for MDLF; 0.75 for IFOF; 0.7 for ILFlat and ILFmed; and 0.75 for AF. The overlap is binarized. (B) Comparison of tract overlap when reconstructed with different techniques. We calculated the number of voxels overlapping between a tract of interest and each of the tracts obtained with FSL probabilistic divided by the total number of voxels of the tract of interest. The darker color represents the overlap of the tract of interest obtained with FSL probabilistic, and the lighter color represents the overlap of the tract of interest obtained with FSL deterministic. Gorilla postmortem data are available from https://doi.org/10.5281/zenodo.3901205. AF, arcuate fascicle; FSL, FMRIB Software Library; IFOF, inferior fronto-occipital fascicle; ILFlat, inferior longitudinal fascicle lateral; ILFmed, inferior longitudinal fascicle medial; MdLF, middle longitudinal fascicle. (TIF) [file pbio.3000810.s014.tif]
