# Supplementary material for: Longitudinal connections and the organization of the temporal cortex in macaques, great apes, and humans
Source: PLoS Biol. 2020 Jul 31;18(7):e3000810. doi: 10.1371/journal.pbio.3000810 (PMC7423156; doi:10.1371/journal.pbio.3000810)
Supplement: S1 Text — (DOCX) [file pbio.3000810.s015.docx]

The research of white matter tracts commonly relies on different methods across species. All of these methods have their advantages and inconveniences and capture different aspects of the underlying biology. Therefore, when comparing the white matter organization in different species, we should use the same technique to be able to compare like-with-like.

We argue here that tractography is the method that fits best our study aim. In the macaque monkey, tract tracing approaches have been previously widely used. They are precise techniques to follow the path of an axon, but require very invasive experiments. This is also the case for blunt dissection studies. Therefore, their use in a large range of primate species including humans is not ethically supported. Tractography, which relies on diffusion MRI data, is a non-invasive alternative and the only viable option as a method available in all species.

There are technical limitations of tractography and if used incorrectly the method can lead to high false positive and false negative rates and biases due to the definition of seeds and waypoints to guide the algorithm. We have dealt with this issue by defining our seeds and waypoints based on a data-driven method clustering together white matter voxels according to their connectivity to the rest of the brain. The underlying idea is that if voxels, around the same anatomical region, have very similar connectivity to the rest of the brain then they should be part of the same tract. This approach was restricted to the core of the tract and the result was compared with outcomes from previous tractography studies but also tract tracing and dissection. In a recent paper from our lab [1], it has been shown that using features from tract tracing studies can help guide reliable tractography results in both humans and macaques; this was illustrated for a tract that is usually particularly difficult to reconstruct with tractography, the amygdalofugal pathway.

To establish a relationship between the probabilistic tractography results and those obtained using other methods, we here also compare our tractography results with tracts obtained with other methods. When using different methods, we cannot expect to obtain exactly the same results. However, we note here that the core of the tracts resulting from our tractography protocol in macaques is very similar to what have been reconstructed using tract tracing results [2] (S1 Fig), indicating that the number of false positives and negatives is low. Specifically, the ILF and MdLF are very much the same, but we can notice some differences for the AF and IFOF (Fig S1). Regarding the AF, it seems to invade the territory of the Superior Longitudinal Fasciculus (SLF) in some parts. However, this is a known issue when using tractography for the AF, as pointed out in the pioneering study from [3] where AF and SLF III are treated as one bundle in humans, chimpanzees and macaques.

Concerning the IFOF, its extent from the frontal lobe to the occipital lobe has been controversial, as tracer data commonly shows terminations in the middle part of the temporal cortex in macaques [4]. We presume this discrepancy to be due to the nature of the IFOF as a multisynaptic pathway. Tract tracing can only reveal mono-synaptic connections and therefore might fail to reconstruct the full IFOF pathway [5]. A recent blunt dissection study showed that an IFOF similar to the one reported by our tractography results can be identified in macaques, running from the frontal lobe to the occipital lobe [6]. We are therefore confident that the fiber bundle identified in this study correspond to tracts previously shown in the literature and that it has a firm biological basis.

We also verified our approach by comparing the results obtained with two other tractography techniques, one using the same software (FSL) but with a deterministic algorithm and one using a completely different package, MRtrix3 ([www.mrtrix.org](http://www.mrtrix.org)). The three different methods yield very similar tracts and anatomical organization (S2_text, S10 Fig). We are therefore confident that the probabilistic tractography results do not only reflect false positives and false negatives.

**References**

1. Folloni D, Sallet J, Khrapitchev AA, Sibson N, Verhagen L, Mars RB. Dichotomous organization of amygdala/temporal-prefrontal bundles in both humans and monkeys. Elife. 2019;8: 1–23. doi:10.7554/eLife.47175

2. Schmahmann JD, Pandya DN, Wang R, Dai G, D’Arceuil HE, De Crespigny AJ, et al. Association fibre pathways of the brain: Parallel observations from diffusion spectrum imaging and autoradiography. Brain. 2007;130: 630–653. doi:10.1093/brain/awl359

3. Rilling JK, Glasser MF, Preuss TM, Ma X, Zhao T, Hu X, et al. The evolution of the arcuate fasciculus revealed with comparative DTI. Nat Neurosci. 2008;11: 426–428. doi:10.1038/nn2072

4. Thiebaut De Schotten M, Dell’Acqua F, Valabregue R, Catani M. Monkey to human comparative anatomy of the frontal lobe association tracts. Cortex. Elsevier Srl; 2012;48: 82–96. doi:10.1016/j.cortex.2011.10.001

5. Mars RB, Foxley S, Verhagen L, Jbabdi S, Sallet J, Noonan MP, et al. The extreme capsule fiber complex in humans and macaque monkeys: a comparative diffusion MRI tractography study. Brain Struct Funct. 2016;221: 4059–4071. doi:10.1007/s00429-015-1146-0

6. Decramer T, Swinnen S, van Loon J, Janssen P, Theys T. White matter tract anatomy in the rhesus monkey: a fiber dissection study. Brain Struct Funct. Springer Berlin Heidelberg; 2018;223: 3681–3688. doi:10.1007/s00429-018-1718-x
