# Supplementary material for: Longitudinal connections and the organization of the temporal cortex in macaques, great apes, and humans
Source: PLoS Biol. 2020 Jul 31;18(7):e3000810. doi: 10.1371/journal.pbio.3000810 (PMC7423156; doi:10.1371/journal.pbio.3000810)
Supplement: S2 Text — (DOCX) [file pbio.3000810.s016.docx]

**Data acquisition and preprocessing: diffusion MRI data**

Four rhesus macaque brains (*Macaca mulatta*, one female, between four and 14 years old at the time of death) were obtained *post mortem*. The brains were stored in formalin, rehydrated in a phosphate-buffered saline solution one week prior to scanning and placed in fomblin for the scanning procedure. *Ex vivo* diffusion-weighted MRI data were acquired from the whole brain using a 7T preclinical MRI scanner. We used a 2D diffusion-weighted spin echo multi slice protocol with single line readout (DW-SEMS; TR = 10000 ms; TE = 25 ms resolution: 0.6 x 0.6 x 0.6 mm^3^, number of slices: 128). Nine non-diffusion-weighted (b = 0 s/mm^2^) and 131 diffusion-weighted (b = 4000 s/mm^2^) volumes were acquired with diffusion encoding directions evenly distributed over the whole sphere (single shell protocol). These data have been used in previous reports [1–4].

Human *in vivo* data were provided by the Human Connectome Project (HCP), WU-Minn Consortium (Principal Investigators: David Van Essen and Kamil Ugurbil; 1U54MH091657) funded by the 16 NIH Institutes and Centers that support the NIH Blueprint for Neuroscience Research; and by the McDonnell Center for Systems Neuroscience at Washington University. We used the datasets of ten subjects from the Q2 release. Data acquisition and preprocessing methods are described in detail in Glasser et al. [5], Sotiropoulos et al. [6] and Ugurbil et al. [7]. In brief, diffusion-weighted MRI data were acquired from the whole brain using a customized 3T Siemens Skyra scanner. A slice-accelerated gradient echo EPI readout was used with a resolution of 1.25 x 1.25 x 1.25 mm^3^. Sampling in q-space included 3 shells at b= 1000, 2000, and 3000 s/mm^2^. For each shell, 90 diffusion encoding gradient directions and six non-diffusion-weighted (b = 0 s/mm^2^) were obtained twice, with the phase encoding direction reversed to enable susceptibility-related distortion correction.

*In vivo* data from three chimpanzees (*Pan troglodytes*, three females, between 13 and 36 years old) were obtained from the National Chimpanzee Brain Resource ([www.chimpanzeebrain.org](http://www.chimpanzeebrain.org)) supported by the NIH National Institute of Neurological Disorders and Stroke (grant no. R24NS092988). Data were obtained from a data archive of scans obtained prior to the 2015 implementation of U.S. Fish and Wildlife Service and National Institutes of Health regulations governing research with chimpanzees. All the scans reported in this publication were completed by the end of 2012. Diffusion-weighted MRI data were acquired at the Yerkes National Primate Research Center (YNPRC) on a 3T MRI scanner under propofol anesthesia (10 mg/kg/h), using previously described procedures [8]. All procedures were carried out in accordance with protocols approved by YNPRC and the Emory University Institutional Animal Care and Use Committee (approval no. YER-2001206). A single-shot spin-echo echo planar sequence (TR = 5900 ms; TE = 86 ms; resolution: 1.8 x 1.8 x 1.8 mm^3^, number of slices: 41) was used to acquire two diffusion-weighted images for each of 60 diffusion directions (b = 1000 s/mm^2^). Each with one of the possible left–right phase-encoding directions and four repeats, to enable susceptibility-related distortion correction. Eight averages of diffusion weighted MRI were collected and in total, 40 volumes (5 per averages) without diffusion weighting (b = 0 s/mm^2^) were also acquired with matching imaging parameters. These data were used in previous reports [8,9].

The data processing for the EPI-based protocols (*in vivo* chimpanzees and humans) consisted of correcting for susceptibility-related distortions using FSL’s TOPUP [10] and correcting for eddy-current using FSL’s eddy tool [11]. For all macaque, human and in-vivo chimpanzee data, fitting of a diffusion tensor model was done using FSL’s dtifit. Following preprocessing, bedpostX was used to fit a crossing fiber-model to the data, allowing for three fiber orientation [12]. They were also registered to their specific standard space, F99 for macaques, MNI152 for humans and Yerkes129 for chimpanzees.

*Post mortem* brains from one Western Lowland gorilla (*Gorilla gorilla gorilla*, male, 12 years old at time of death) and one chimpanzee (*Pan troglodytes*, female, 28 years old at time of death) were obtained from the London Zoological Society and the Primate Brain Bank, respectively. Both samples were stored in formalin, rehydrated using a phosphate-buffered saline solution one week prior to scanning and placed in fluorinert for the scanning procedure. As the left frontal lobe of the gorilla brain was damaged during extraction, we have only used the diffusion MRI data from the right hemisphere. The left hemisphere in the chimpanzee sample was slightly flattened in the extraction protocols, making it hard to interpret any results on this hemisphere, so we will also focus on the right hemisphere. Both samples were imaged using a 7T whole body scanner with a 28-channel knee coil (QED). Diffusion MRI data were acquired using a 3D diffusion weighted steady state free precession (DW-SSFP) pulse sequence [13,14]. DW-SSFP data comprising of 240 diffusion weighted (q = 300 cm^-1^, flip angle = 39°, TR = 28 ms, TE =21 ms, resolution = 0.6 x 0.6 x 0.6 mm^3^) and six non-diffusion weighted (q = 20 cm^-1^) imaging volumes were acquired over the whole postmortem brains. Two repeats of this protocol were performed for the gorilla, with a single repeat for the chimpanzee. To account for the T1, T2 and flip-angle dependencies of the DW-SSFP signal [15,16], T1, T2 and B1 datasets were acquired via a turbo inversion-recovery (TIR) (TIs = 30, 60, 120, 240, 480 and 939 ms, TR = 1000 ms, TE = 13 ms, resolution = 0.4 x 0.4 x 1.0 mm^3^), turbo spin-echo (TSE) (TEs = 12, 25, 37, 49, 61 and 74 ms, TR = 1000 ms, resolution = 0.4 x 0.4 x 1.0 mm^3^) and actual flip angle imaging (AFI) acquisition (flip angle = 80°, TR1 = 7 ms, TR2 = 21 ms, resolution = 1.5 x 1.5 x 1.5 mm^3^ for the gorilla, flip angle = 60°, TR1 = 17 ms, TR2 = 11 ms, TE = 1.5 ms, resolution = 2.8 x 2.8 x 3.0 mm^3^ for the chimpanzee)[17].

Prior to processing, a Gibbs ringing correction [18] was applied to the DW-SSFP, TIR and TSE datasets. Quantitative T1 and T2 maps were generated from the TIR and TSE datasets assuming mono-exponential signal evolution. A B1 map was generated from the AFI data following the methodology described in [17]. All coregistrations within and between imaging modalities were performed with FLIRT [19,20] via a six degrees of freedom (translation and rotation) transformation. The DW-SSFP data, along with the T1, T2 and B1 maps were fitted with the full DW-SSFP signal equation [15] to both a diffusion tensor model and a ball and two stick model using cuDIMOT [21].

**Data acquisition and preprocessing: structural and surface data**

For macaques, we used a model of the cortical surface in the standard space F99. Human T1- and T2-weighted images were acquired using an MPRAGE sequence at 0.7 x 0.7 x 0.7 mm^3^ resolution and aligned to the diffusion space as part of the HCP's minimum preprocessing pipeline [5]. The HCP pipeline reconstructs the pial and white/grey matter interface surface by combining T1- and T2-weighted scans. The surfaces were modelled for each individual as provided through the HCP pipeline [5], and an HCP average surface was used to display the results. Similarly, for the chimpanzee *in vivo* data, T1- and T2-weighted images were processed using a modified version of the HCP pipeline [5,22]. The surfaces were modelled for each individual and an average surface was used to display the results (Yerkes129).

Cortical surfaces for the *ex vivo* gorilla and chimpanzee specimens were generated using CARET version 5.65 [23]. For the chimpanzee, a structural scan was generated using a true fast imaging with steady free precession (TRUFI) sequence [13]. Four high resolution *ex* vivo TRUFI datasets were acquired over the entire post-mortem brain, where each dataset was obtained with a different phase increment per TR (resolution = 0.21 x 0.21 x 0.19 mm^3^, flip angle = 30°, TE = 6.78 ms, TR = 13.56 ms, phase increments = 0°, 90°, 180° and 270°). The four TRUFI datasets were averaged via root sum of squares to form a structural scan that is uncorrupted by the banding artefacts found in individual images [24]. For the gorilla, we formed a structural scan from the mean diffusion attenuation map (diffusion weighted / non-diffusion weighted) over all directions from the acquired DW-SSFP data (See Data acquisition and preprocessing: diffusion MRI data for details of this acquisition). We did not use the conventional T1-weigthed images for these *post mortem* samples because they do not have a good contrast in fixed brains due to the T1 values of grey and white matter converging. TRUFI (chimpanzee) and diffusion attenuation (gorilla) images have a good contrast but opposite to conventional T1-weighted. Therefore, we needed to include further pre-processing to approximate the T1-weighted *in vivo* intensity profile required for surface reconstruction by CARET. This intensity correction was done as follows after bias correcting these structural scans using FSL’s FAST [25]. Using the Mango MRI editing software [26] a region of interest (ROI) was carefully constructed semi-manually so that it only contained white and grey matter brain voxels. The range of voxel values within the ROI was normalized to the interval 0 to 1 and then subtracted from 1 to invert the intensities. All voxels outside the ROI were set to 0. These pre-processed data were imported into CARET 5.65 and ﻿reoriented to be approximately parallel to the anterior commissure–posterior commissure (ACPC) line. Accurate surfaces were obtained semi-automatically by iteratively generating and adjusting “Surefit” segmentations within CARET. The resulting surfaces were smoothed in the medial wall and lesioned areas. We applied smoothing in the medial wall because this non-cortical area would not be defined otherwise on the cortical surface. The lesioned areas needed to be smoothed to recover the true anatomy of these areas otherwise misunderstood by the algorithm.

**Cluster number verification**

As stated in the main text, in our initial analyses we perform a connectivity-based parcellation of coronal sections through the temporal cortex white matter (Fig. 9A) to establish the bodies of main white matter tracts that we then reconstruct (Fig. 9B). We hypothesize three main clusters for the macaque, corresponding to the MdLF, IFOF, and ILF, and four clusters for the human, corresponding to MdLF, IFOF, and two ILF subdivisions based on the work by Latini and colleagues [27].

To determine the number of clusters in data, different algorithms have been used (see [28] for a review). However, in the connectivity-based parcellation it is well-known that different algorithms tend to converge on different solutions. Therefore, previous studies have used multiple algorithms and chosen a solution that was appropriate to the point they wanted to convey [29–32]. However, the situation might be alleviated in the comparative context, where we are merely interested in whether all solutions point in the same directions between different brains, rather than in ‘the’ correct number of clusters. In this respect, our hypothesis is that great ape and human brains show a greater number of clusters in the temporal cortex white matter, which we interpret as a greater complexity of the white matter. We want to precise that we do not claim that the exact number of cluster should be three in macaques and four in apes. Rather, we want to show that by using the same approach in the different brains and finding a larger number of clusters in some brains than in others, we can make a comparative statement about the complexity of the brains.

Below, we describe various ways of assessing cluster number. We originally tested the number of clusters by means of consistency across subjects, which has been the preferred method in our lab in the past [29,33,34]. We then also employed the hierarchy index, a popular measure that relies on the hierarchical nature of biological data as a confirmatory measure. Finally, to further test the hypothesis of greater complexity in great apes, we employ a variety of other methods from the literature and, acknowledging that they are likely to give slightly different absolute numbers of clusters, investigate whether each method indicates a greater number of clusters in the great apes and humans compared to the macaque ‘baseline’ number of clusters.

We used the consistency across subjects as the main goal of the clustering was to define the masks for the subsequent tract reconstruction. These masks are defined based on probability maps from the clustering of the different subjects. Therefore, if there is no consistency across subjects in the clustering result, the maps will contain only low probability and it will not be possible to define non-overlapping masks for the tract reconstruction. When we ran the clustering analysis with one more cluster in macaques (four for the posterior and middle ROIs and five for the anterior ROI), we noticed that the clustering would then lead to divide either the MdLF, IFOF or ILF associated cluster without consistency across subjects and ROIs (S8A Fig). In contrast in humans and chimpanzees, when we ran the clustering analysis with one less cluster (three for the posterior and middle ROIs and four for the anterior ROI), we could notice that the clustering would cluster together the two ILF sub-components (S8A Fig). This consistency analysis confirmed that a higher number of clusters in the macaque did not yield a reliable parcellation of white matter. This result is also in line with the results from the hierarchy analysis.

We further verified the choice of clusters by assessing the hierarchical nature of the cluster assignment in the low versus the high-cluster solution. Many studies on cortical organization have shown a hierarchical organization, where one can define organization from networks, to clusters of areas, to areas, to patches, etc. This hierarchical organization means that when one divides a larger part (e.g., a brain network) into more constituency parts (e.g., brain areas) no aspect (e.g., voxel) will jump from one high-level unit to another. In other words, all voxels classified as belonging to the same brain areas previously belonged to the same brain network. If this is not the case, the finer level parcellation is likely to be incorrect. We tested whether our choice of ILF subdivisions in humans and great apes, but not macaques, was consistent with this notion in two way. First, as part of the consistency analysis, we added the two clusters from the two-components solution (i.e. high number of clusters solution) for all individuals and calculated its overlap with the cluster from the one ILF solution for all individuals (i.e. low number of cluster solution). This showed a larger overlap in humans compared to macaques (S8B Fig). Thus, in humans a three-cluster solution would lead to a cluster associated with ILF that we could reliably split into two sub-components in a four-cluster solution, but this was not the case in the macaque. In both gorilla and chimpanzee, the difference between a three- and four-cluster solution led to a separation of ILF into two subcomponents although less reliable than in the human (S8A Fig, S8B Fig).

We performed a more formal test by calculating the Hierarchy Index [30] between the three-clusters and four-clusters solutions in the middle ROI. This index ﻿represents the probability that a given cluster in the four-clusters solution comes from only one cluster in the three-clusters solution. A perfectly hierarchical subdivision would have a hierarchy index equal to one. We found that for all samples apart from the macaque the hierarchy index was close to one (0.93 for gorilla and 0.96 and 0.95 for chimpanzees and humans respectively when averaged over the two hemispheres, S8C Fig). In contrast, the macaque’s hierarchy index, again averaged over the two hemispheres, was only 0.72, much closer to the value observed for hierarchy based on 1000 random permutations of cluster labels (0.40, average over all species and hemispheres of S8C Fig). As the macaque was the only one demonstrating such a low hierarchy index, departing from the perfect value of one, compared to other species, this reinforces the argument in favor of a three-clusters solution in macaques and four-clusters solution in others. The difference between the human and macaque can also be represented graphically. Figure S9 demonstrates which cluster in the low-cluster solution each voxel of ILFlat and ILFmed in a high-cluster solution was assigned to. As can be seen, human ILFmed and ILFlat voxels were previously almost exclusively assigned to ILF, whereas macaque ILFmed voxels in the left hemisphere and ILFlat voxels in both hemispheres were assigned almost equally from ILF and IFOF in low-cluster solution.

Finally, to further confirm the higher complexity found in great apes than in macaques, we performed the clustering of the middle ROI in all species imposing one to five clusters and calculated four additional measures that should indicate the best number of clusters among the ones tried (Calinski-Harabasz [35], Davies-Bouldin [36], silhouette [37] and gap statistics [38]). We performed the Calinski-Harabasz, Davies-Bouldin and silhouette measure as part of the in-built Matlab function `evalclusters`. The gap statistic was performed using the Matlab file exchange function written by Alessandro Crimi (<https://www.mathworks.com/matlabcentral/fileexchange/37905-gap-statistics>) and modified to use a normal distribution as the reference distribution (as suggested in [39]). Although, as shown in previous literature, the different measures differed in their final solution [28], we report here that they still point toward a higher complexity for the humans and chimpanzees than for the macaques, with a more ambiguous result for the gorilla. To represent this result efficiently, the ideal number of clusters given for macaques were subtracted from the ideal number given for the apes by each of the four measures. The motivation behind this manipulation is to show that if this subtraction leads to positive number in general, it means that the complexity is higher for the apes. In other words, that the measures point toward a higher number of cluster for apes than macaques (S8D Fig). We also included, the results from the hierarchy and correspondence analysis in this representation, which point toward a four cluster solution as a better solutions for apes and a three cluster solution as a better solution for macaques.

**Clustering and tract reconstruction using other tractography techniques**

Tractography algorithms can be error prone [40]. This is particularly true when tractography is performed in a manner that is unconstrained by the use of anatomical priors[41]. However, these errors can be different when using different algorithms. Therefore, to mitigate the possibility that some of the results may be affected by false positive/negative tracts, we repeated our analyses using different tractography techniques. In addition to the probabilistic tractography using FSL from the main text, we performed deterministic tracking using FSL and an entirely separate implementation from a separate software suite, namely MRTrix3 ([www.mrtrix.org](http://www.mrtrix.org)).

First, we investigated whether we could replicate the first part of the analysis pipeline, the connectivity-based clustering. We performed the same unconstrained tractography as previously described from all the voxels in temporal lobe white matter ROIs to all the voxels in the brain, but using deterministic tractography instead of probabilistic. In order to do so, we transformed the outputs of the bedpostX model (macaque, chimpanzee *in vivo,* humans) and SSFP-based ball and two stick model (gorilla) to use only the peak orientations of the uncertainty distribution for subsequent deterministic tractography with FSL probtrackx2. We also turned off probabilistic interpolation [42] and instead used nearest neighbor interpolation, i.e. similar to traditional deterministic tracking. However, unlike traditional deterministic algorithms, we did not save individual streamlines, but we instead saved voxel-wise visitation counts which were directly comparable with the probabilistic tractography results. Streamline seeding was randomly jittered within each voxel. We increased this jitter for the deterministic version to allow for a sufficient amount of streamlines to be generated (0.25 for macaques, 0.3 for gorilla, 0.5 for chimpanzees and humans). All other parameters were kept the same and we were then able to follow the exact same procedure as defined for the probabilistic version. Briefly, we computed the similarity matrix representing how each voxel in the in temporal lobe white matter ROIs is connected to the rest of the brain. This similarity matrix underwent k-means clustering to reveal which voxels of the ROI share similar connectivity. With the same logic as before, based on the current knowledge of tract anatomy [27,43,44], we could determine which cluster belonged to each tract for each subject. Adding the clusters belonging to the same tracts for all subjects, we obtained probability maps of cluster position in each ROI.

We tested the similarity of these cluster positions with the ones we found using the probabilistic algorithm. For both clustering results, we obtained a winner-takes-all map from the probability maps, which assigns each voxel of the ROI to the tract cluster with the highest probability. We compared the probabilistic and deterministic winner-takes-all maps by calculating the Dice coefficient between these two maps for each ROI of each species. This coefficient assesses how two clustering results are similar to each other [45,46]. We obtained Dice coefficients ranging between 0.59 and 0.98 (mean across all ROIs and all species of 0.80). Importantly, all of these coefficients were significantly different to coefficients calculated using 1000 randomly permuted clustering results (difference tested at p < 0.01 using permutation test, mean of Dice coefficient between permuted results of 0.27, S10 Fig). The randomly permuted clustering results were obtained by assigning randomly one of the possible cluster identification to each voxels. In other words, by randomizing the order of the values in the vectors representing the cluster identification of each voxel in the ROIs obtained with the two techniques and calculating the Dice coefficient between these two randomized vectors. This result reinforces our confidence on the position of the clusters described previously. A visual inspection also shows that the clusters obtained with both techniques are very similar (S10 Fig).

Second, we investigated whether we can replicate the tract reconstruction step of our analysis pipeline. We performed the same tractography as previously described, starting from the seed masks and constrained by two waypoints which were anterior and posterior to the seed. All masks for the MdLF, IFOF and ILF had been defined in the clustering step. For AF, we used the masks as described earlier. We also used the same exclusion and termination masks described previously. However, for the tractography itself we used two different tractography techniques.

One was the deterministic tractography from FSL, which we used as described for deterministic clustering. We also changed here the jitter of the streamline random sampling from the seed (from 1mm with the probabilistic algorithm to 3mm for all species with the deterministic algorithm). We were then able to follow the exact same procedure as defined for the probabilistic version. We normalized each tract by the number of streamlines generated, averaged the tracts across species and down-sampled the results before log-transforming them.

The other technique used for this tractography step was performed using the MRtrix3 package ([www.mrtrix.org](http://www.mrtrix.org)). We followed guidelines from the developers of this package as to which fitting model to use with the different preprocessed data in our hands. As the gorilla and chimpanzee *post mortem* brains were scanned using diffusion-weighted steady-state free precession (DW-SSFP) protocols, we were not able to use MRtrix to analyse these data. This is because DW-SSFP has a different signal formation mechanism, which requires different modelling [13], compared to spin-echo data which MRtrix was designed for. The *in vivo* chimpanzee data, while being spin-echo, have a resolution that is considered too low for the type of algorithm used by MRtrix. Therefore, we could only perform this step on the ex-vivo macaque and human data. To estimate the fiber orientation distribution, MRtrix first requires to estimate a response function modelling the signal expected at a given high-anisotropy voxel and uses it as a kernel to perform constrained spherical deconvolution. In humans, we used the Dhollander algorithm to estimate the response function [47,48], because it is described as the default and best suited for multi-shell data. Then we applied a multi-shell multi-tissue constrained spherical deconvolution [49] which estimates the fiber orientation distributions for each tissue type (white matter, grey matter and cerebrospinal fluid). We performed intensity normalization to correct for global intensity differences to make the fiber orientation distributions comparable between subjects. In macaques, we estimated the response function using the Tournier algorithm [50] because it is described as being robust with a wide range of single shell data. Then, we applied the constrained spherical deconvolution to estimate the fiber orientation distributions of the white matter [51]. To finally perform the streamlines tractography, we used the tckgen tool from MRtrix with the default probabilistic algorithm iFOD2, which stands for second order integration over Fiber Orientation Distributions Where possible, similar settings to the FSL-based probabilistic tractography technique were applied. We used the same masks (only converted to MRtrix format), the step sizes were species-dependent (0.5 for humans and 0.25 for macaques), and we seeded 10000 streamlines per voxel of the seed image to match the number of samples used in FSL. Finally, MRtrix implementation of waypoint inclusion imposes that streamlines must traverse all waypoint masks to be accepted while FSL allows to traverse only one. Therefore, we conducted the MRtrix tractography once with the waypoint anterior and once with the posterior one and added the two results. As for the other tractography techniques, we transformed the results of each individual back to standard space and normalized the tracts by dividing them by the number of streamlines generated, then averaged the tracts across species and down-sampled the results before log-transforming them (S5 Fig).

Anatomically, the tracts obtained with the different techniques followed a very similar path and confirmed their characteristics described earlier (S11-S14 Fig, panels A). The tracts obtained by computing the overlap between the different techniques also demonstrates the overall conserved pattern (S11-S14 Fig, panels A Overlap). In all analyses, the main course of the tracts is preserved, with MdLF running dorsally to ILF and IFOF generally running more medially. The IFOF was the only one of these three to reach the frontal cortex in any species. Although this frontal extension was less pronounced in the deterministic tracking, the overall course of the tract was similar. The AF was also successfully reconstructed in all species using all techniques and the temporal extension in the human was apparent.

We also computed the overlap of each tract obtained with the different techniques with the tracts obtained with FSL probabilistic tractography. More precisely this overlap was calculated as the number of voxels overlapping between a tract of interest and each of the tracts obtained with FSL probabilistic divided by the total number of voxels of the tract of interest. The pattern of overlap of the tracts obtained with other techniques is similar to the one obtained with FSL probabilistic and the tracts most overlapping is always the one we were trying to reconstruct (S11-S14 Fig, panels B). Therefore, not only the tracts have a similar pathway, they also have a similar organization with respect to each other across the different techniques.

Overall, the different results (clustering and tract reconstruction) obtained using the FSL probabilistic tractography could be reproduced with other techniques, arguing for the robustness of our results and against them emanating from false positives.

**References**

1. Eichert N, Robinson EC, Bryant KL, Jbabdi S, Jenkinson M, Li L, et al. Cross-species cortical alignment identifies different types of anatomical reorganization in the primate temporal lobe. Elife. 2020;9. doi:10.7554/eLife.53232

2. Folloni D, Sallet J, Khrapitchev AA, Sibson N, Verhagen L, Mars RB. Dichotomous organization of amygdala/temporal-prefrontal bundles in both humans and monkeys. Elife. 2019;8: 1–23. doi:10.7554/eLife.47175

3. Mars RB, Sotiropoulos SN, Passingham RE, Sallet J, Verhagen L, Khrapitchev AA, et al. Whole brain comparative anatomy using connectivity blueprints. Elife. 2018;7: 245209. doi:10.7554/eLife.35237

4. Eichert N, Verhagen L, Folloni D, Jbabdi S, Khrapitchev AA, Sibson NR, et al. What is special about the human arcuate fasciculus? Lateralization, projections, and expansion. Cortex. Elsevier Ltd; 2018; 1–9. doi:10.1016/J.CORTEX.2018.05.005

5. Glasser MF, Sotiropoulos SN, Wilson JA, Coalson TS, Fischl B, Andersson JL, et al. The minimal preprocessing pipelines for the Human Connectome Project. Neuroimage. Elsevier Inc.; 2013;80: 105–124. doi:10.1016/j.neuroimage.2013.04.127

6. Sotiropoulos SN, Jbabdi S, Xu J, Andersson JL, Moeller S, Auerbach EJ, et al. Advances in diffusion MRI acquisition and processing in the Human Connectome Project. Neuroimage. Elsevier Inc.; 2013;80: 125–143. doi:10.1016/j.neuroimage.2013.05.057

7. Ugurbil K, Xu J, Auerbach EJ, Moeller S, Vu AT, Duarte-Carvajalino JM, et al. Pushing spatial and temporal resolution for functional and diffusion MRI in the Human Connectome Project. Neuroimage. 2013;80: 80–104. doi:10.1016/j.neuroimage.2013.05.012

8. Chen X, Errangi B, Li L, Glasser MF, Westlye LT, Fjell AM, et al. Brain aging in humans, chimpanzees (pan troglodytes), and rhesus macaques (macaca mulatta): Magnetic resonance imaging studies of macro- and microstructural changes. Neurobiol Aging. Elsevier Ltd; 2013;34: 2248–2260. doi:10.1016/j.neurobiolaging.2013.03.028

9. Mars RB, O’Muircheartaigh J, Folloni D, Li L, Glasser MF, Jbabdi S, et al. Concurrent analysis of white matter bundles and grey matter networks in the chimpanzee. Brain Struct Funct. Springer Berlin Heidelberg; 2018;0: 0. doi:10.1007/s00429-018-1817-8

10. Andersson JLR, Skare S, Ashburner J. How to correct susceptibility distortions in spin-echo echo-planar images: Application to diffusion tensor imaging. Neuroimage. 2003;20: 870–888. doi:10.1016/S1053-8119(03)00336-7

11. Andersson JLR, Sotiropoulos SN. An integrated approach to correction for off-resonance effects and subject movement in diffusion MR imaging. Neuroimage. 2016;125: 1063–1078. doi:10.1016/j.neuroimage.2015.10.019

12. Behrens TEJ, Berg HJ, Jbabdi S, Rushworth MFS, Woolrich MW. Probabilistic diffusion tractography with multiple fibre orientations: What can we gain? Neuroimage. 2007;34: 144–155. doi:10.1016/j.neuroimage.2006.09.018

13. Miller KL, McNab JA, Jbabdi S, Douaud G. Diffusion tractography of post-mortem human brains: Optimization and comparison of spin echo and steady-state free precession techniques. Neuroimage. 2012;59: 2284–2297. doi:10.1016/j.neuroimage.2011.09.054

14. Foxley S, Jbabdi S, Clare S, Lam W, Ansorge O, Douaud G, et al. Improving diffusion-weighted imaging of post-mortem human brains: SSFP at 7T. Neuroimage. Elsevier B.V.; 2014;102: 579–589. doi:10.1016/j.neuroimage.2014.08.014

15. Buxton RB. The diffusion sensitivity of fast steady-state free precession imaging. Magn Reson Med. 1993;29: 235–243. doi:10.1002/mrm.1910290212

16. McNab JA, Miller KL. Steady-state diffusion-weighted imaging: Theory, acquisition and analysis. NMR Biomed. 2010;23: 781–793. doi:10.1002/nbm.1509

17. Yarnykh VL. Actual flip-angle imaging in the pulsed steady state: A method for rapid three-dimensional mapping of the transmitted radiofrequency field. Magn Reson Med. 2007;57: 192–200. doi:10.1002/mrm.21120

18. Kellner E, Dhital B, Kiselev VG, Reisert M. Gibbs-ringing artifact removal based on local subvoxel-shifts. Magn Reson Med. 2016;76: 1574–1581. doi:10.1002/mrm.26054

19. Jenkinson M, Smith S. A global optimisation method for robust affine registration of brain images. Med Image Anal. 2001;5: 143–156. doi:10.1016/S1361-8415(01)00036-6

20. Jenkinson M, Bannister P, Brady M, Smith S. Improved Optimization for the Robust and Accurate Linear Registration and Motion Correction of Brain Images. Neuroimage. 2002;17: 825–841. doi:10.1006/nimg.2002.1132

21. Hernandez-Fernandez M, Reguly I, Jbabdi S, Giles M, Smith S, Sotiropoulos SN. Using GPUs to accelerate computational diffusion MRI: From microstructure estimation to tractography and connectomes. Neuroimage. Elsevier Ltd; 2019;188: 598–615. doi:10.1016/j.neuroimage.2018.12.015

22. Donahue CJ, Sotiropoulos SN, Jbabdi S, Hernandez-Fernandez M, Behrens TEJ, Dyrby TB, et al. Using Diffusion Tractography to Predict Cortical Connection Strength and Distance: A Quantitative Comparison with Tracers in the Monkey. J Neurosci. 2016;36: 6758–6770. doi:10.1523/jneurosci.0493-16.2016

23. Van Essen DC, Drury HA, Dickson J, Harwell J, Hanlon D, Anderson CH. An integrated software suite for surface-based analyses of cerebral cortex. J Am Med Informatics Assoc. 2001;8: 443–459. doi:10.1136/jamia.2001.0080443

24. Bangerter NK, Hargreaves BA, Vasanawala SS, Pauly JM, Gold GE, Nishimura DG. Analysis of Multiple-Acquisition SSFP. Magn Reson Med. 2004;51: 1038–1047. doi:10.1002/mrm.20052

25. Zhang Y, Brady M, Smith S. Segmentation of brain MR images through a hidden Markov random field model and the expectation-maximization algorithm. IEEE Trans Med Imaging. 2001;20: 45–57. doi:10.1109/42.906424

26. Lancaster JL, McKay DR, Cykowski MD, Martinez MJ, Tan X, Valaparla S, et al. Automated analysis of fundamental features of brain structures. Neuroinformatics. 2011;9: 371–380. doi:10.1007/s12021-011-9108-z

27. Latini F, Mårtensson J, Larsson E, Fredrikson M, Åhs F. Segmentation of the inferior longitudinal fasciculus in the human brain : A white matter dissection and diffusion tensor tractography study. Brain Res. Elsevier B.V.; 2017;1675: 102–115. doi:10.1016/j.brainres.2017.09.005

28. Eickhoff SB, Thirion B, Varoquaux G, Bzdok D. Connectivity‐based parcellation: Critique and implications. Hum Brain Mapp. 2015;36: 4771–4792. doi:10.1002/hbm.22933

29. Neubert FX, Mars RB, Thomas AG, Sallet J, Rushworth MFS. Comparison of Human Ventral Frontal Cortex Areas for Cognitive Control and Language with Areas in Monkey Frontal Cortex. Neuron. Elsevier Inc.; 2014;81: 700–713. doi:10.1016/j.neuron.2013.11.012

30. Kahnt T, Chang LJ, Park SQ, Heinzle J, Haynes J-D. Connectivity-Based Parcellation of the Human Orbitofrontal Cortex. J Neurosci. 2012;32: 6240–6250. doi:10.1523/JNEUROSCI.0257-12.2012

31. Kelly C, Uddin LQ, Shehzad Z, Margulies DS, Castellanos FX, Milham MP, et al. Broca’s region: linking human brain functional connectivity data and non-human primate tracing anatomy studies. Eur J Neurosci. 2010;32: 383–398. doi:10.1111/j.1460-9568.2010.07279.x

32. Clos M, Amunts K, Laird AR, Fox PT, Eickhoff SB. Tackling the multifunctional nature of Broca’s region meta-analytically: Co-activation-based parcellation of area 44. Neuroimage. Elsevier Inc.; 2013;83: 174–188. doi:10.1016/j.neuroimage.2013.06.041

33. Mars RB, Jbabdi S, Sallet J, O’Reilly JX, Croxson PL, Olivier E, et al. Diffusion-Weighted Imaging Tractography-Based Parcellation of the Human Parietal Cortex and Comparison with Human and Macaque Resting-State Functional Connectivity. J Neurosci. 2011;31: 4087–4100. doi:10.1523/JNEUROSCI.5102-10.2011

34. Sallet J, Mars RB, Noonan MP, Neubert F-X, Jbabdi S, O’Reilly JX, et al. The Organization of Dorsal Frontal Cortex in Humans and Macaques. J Neurosci. 2013;33: 12255–12274. doi:10.1523/JNEUROSCI.5108-12.2013

35. Calinski T, Harabasz J. A dendrite method for cluster analysis. Commun Stat - Theory Methods. 1974;3: 1–27. doi:10.1080/03610927408827101

36. Davies DL, Bouldin DW. A Cluster Separation Measure. IEEE Trans Pattern Anal Mach Intell. 1979;PAMI-1: 224–227. doi:10.1109/TPAMI.1979.4766909

37. Rousseeuw PJ. Silhouettes: A graphical aid to the interpretation and validation of cluster analysis. J Comput Appl Math. 1987;20: 53–65. doi:10.1016/0377-0427(87)90125-7

38. Tibshirani R, Walther G, Hastie T. Estimating the number of clusters in a data set via the gap statistic. J R Stat Soc Ser B (Statistical Methodol. 2001;63: 411–423. doi:10.1111/1467-9868.00293

39. Yin Z, Zhou X, Bakal C, Li F, Sun Y, Perrimon N, et al. Using iterative cluster merging with improved gap statistics to perform online phenotype discovery in the context of high-throughput RNAi screens. BMC Bioinformatics. 2008;9: 264. doi:10.1186/1471-2105-9-264

40. Jbabdi S, Johansen-Berg H. Tractography: Where Do We Go from Here? Brain Connect. 2011;1: 169–183. doi:10.1089/brain.2011.0033

41. Maier-Hein KH, Neher PF, Houde J-C, Côté M-A, Garyfallidis E, Zhong J, et al. The challenge of mapping the human connectome based on diffusion tractography. Nat Commun. 2017;8: 1349. doi:10.1038/s41467-017-01285-x

42. Behrens TEJ, Johansen-Berg H, Woolrich MW, Smith SM, Wheeler-Kingshott CAM, Boulby PA, et al. Non-invasive mapping of connections between human thalamus and cortex using diffusion imaging. Nat Neurosci. 2003;6: 750–757. doi:10.1038/nn1075

43. Schmahmann JD, Pandya DN, Wang R, Dai G, D’Arceuil HE, De Crespigny AJ, et al. Association fibre pathways of the brain: Parallel observations from diffusion spectrum imaging and autoradiography. Brain. 2007;130: 630–653. doi:10.1093/brain/awl359

44. Catani M, Thiebaut De Schotten M. A diffusion tensor imaging tractography atlas for virtual in vivo dissections. Cortex. 2008;44: 1105–1132. doi:10.1016/j.cortex.2008.05.004

45. O’Muircheartaigh J, Jbabdi S. Concurrent white matter bundles and grey matter networks using independent component analysis. Neuroimage. Elsevier; 2018;170: 296–306. doi:10.1016/j.neuroimage.2017.05.012

46. Parisot S, Arslan S, Passerat-Palmbach J, Wells WM, Rueckert D. Tractography-Driven Groupwise Multi-scale Parcellation of the Cortex. 2015. pp. 600–612. doi:10.1007/978-3-319-19992-4_47

47. Dhollander T, Raffelt D, Connelly A. Unsupervised 3-tissue response function estimation from single-shell or multi-shell diffusion MR data without a co-registered T1 image. ISMRM Work Break Barriers Diffus MRI. 2016; 5. doi:307863133

48. Dhollander T, Raffelt D, Connelly A. Accuracy of response function estimation algorithms for 3-tissue spherical deconvolution of diverse quality diffusion MRI data. 26th Int Soc Magn Reson Med. 2018; 3–6. Available: https://www.researchgate.net/publication/324770874_Accuracy_of_response_function_estimation_algorithms_for_3-tissue_spherical_deconvolution_of_diverse_quality_diffusion_MRI_data

49. Jeurissen B, Tournier J-D, Dhollander T, Connelly A, Sijbers J. Multi-tissue constrained spherical deconvolution for improved analysis of multi-shell diffusion MRI data. Neuroimage. Elsevier Inc.; 2014;103: 411–426. doi:10.1016/j.neuroimage.2014.07.061

50. Tournier J-D, Calamante F, Connelly A. Determination of the appropriate b value and number of gradient directions for high-angular-resolution diffusion-weighted imaging. NMR Biomed. 2013;26: 1775–1786. doi:10.1002/nbm.3017

51. Tournier J-D, Calamante F, Connelly A. Robust determination of the fibre orientation distribution in diffusion MRI: Non-negativity constrained super-resolved spherical deconvolution. Neuroimage. 2007;35: 1459–1472. doi:10.1016/j.neuroimage.2007.02.016
